# The Dynamics of Inducible Genetic Circuits*

**Published:** 2026-03-04

**Authors:** Zitao Yang, Rebecca J. Rousseau, Sara D. Mahdavi, Hernan G. Garcia, Rob Phillips

**Affiliations:** 1 Department of Physics, California Institute of Technology, Pasadena, CA 91125; 2 Division of Biology and Biological Engineering, California Institute of Technology, Pasadena, CA 91125; 3 Biophysics Graduate Group, University of California, Berkeley, CA 904720; 4 Department of Physics, University of California, Berkeley, CA 94720; 5 Institute for Quantitative Biosciences-QB3, University of California, Berkeley, CA 94720; 6 Department of Molecular and Cell Biology, University of California, Berkeley, CA 94720; 7 Chan Zuckerberg Biohub–San Francisco, San Francisco, CA 94158

## Abstract

Genes are connected in complex networks of interactions where often the product of one gene is a transcription factor that alters the expression of another. Many of these networks are based on a few fundamental motifs leading to switches and oscillators of various kinds. And yet, there is more to the story than which transcription factors control these various circuits. These transcription factors are often themselves under the control of effector molecules that bind them and alter their level of activity. Traditionally, much beautiful work has shown how to think about the stability of the different states achieved by these fundamental regulatory architectures by examining how parameters such as transcription rates, degradation rates and dissociation constants tune the circuit, giving rise to behavior such as bistability. However, such studies explore dynamics without asking how these quantities are altered in real time in living cells as opposed to at the fingertips of the synthetic biologist’s pipette or on the computational biologist’s computer screen. In this paper, we make a departure from the conventional dynamical systems view of these regulatory motifs by using statistical mechanical models to focus on endogenous signaling knobs such as effector concentrations rather than on the convenient but more experimentally remote knobs such as dissociation constants, transcription rates and degradation rates that are often considered. We also contrast the traditional use of Hill functions to describe transcription factor binding with more detailed thermodynamic models. This approach provides insights into how biological parameters are tuned to control the stability of regulatory motifs in living cells, sometimes revealing quite a different picture than is found by using Hill functions and tuning circuit parameters by hand.

## I. INTRODUCTION

The first half of the twentieth century was a time in which many of the great mysteries of nineteenth century physics were resolved [1–4]. Though perhaps less well known, the study of living organisms had enormous mysteries of its own including the molecular and cellular basis of the laws of heredity [5]. One key puzzle centered on a phenomenon known at the time as “enzymatic adaptation” [6, 7]. Those words refer to the apparent induction of enzyme action as a result of changes in the metabolic or physiological state of cells, such as those that occur upon shifting from one carbon source to another [7]. In the nineteenth century, Louis Pasteur had noted that yeasts behave differently under different growth conditions. Frédéric Dienert followed up on those observations with great foresight by doing experiments that quantitatively characterized the phenomenon [7]. Jacques Monod made these studies a fine art through the use of bacterial growth curves “as a method for the study of bacterial physiology and biochemistry” [8] (see Figure 9 of Monod’s paper for a compelling example of the induction phenomenon).

**Figure 1:**
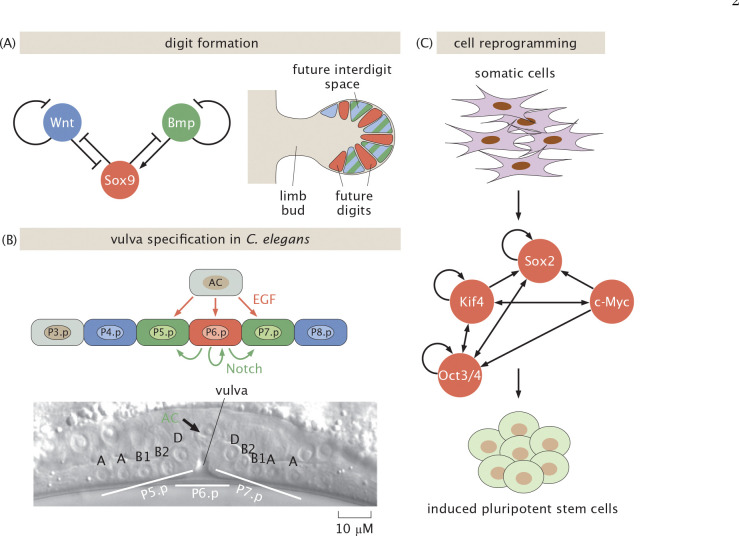
Gallery of examples of regulatory circuits participating in the genetic decisions of animal development. (A) A three-node network thought to be relevant to the control of digit formation, adapted from [17]. (B) An example involving vulval development in *C. elegans*, where epidermal growth factor (EGF) and Notch induce cells toward one of three possible fates, adapted from [18] and [19]. (C) Transcription factors compete and maintain cell pluripotency unless sufficiently induced to reprogram a cell to a differentiated fate, adapted from [20].

As a result of studies like these, in the early 1960s Jacob and Monod shook the world of biology by showing that there are genes whose job it is to control other genes [5, 9], culminating in their repressor-operator model which showed how proteins could bind to DNA and repress the expression of nearby genes [5, 9–11]. Their original work was extended and amplified through the discovery of architectures that were mediated not only by repression, but by activation as well [12], and even by combinations of activators and repressors [13]. In the late 1960s, the vision was considerably broadened through the generalization of these ideas from their first context in bacteria to the much broader set of regulatory problems associated with animal development such as those schematized in Fig. 1 [14]. The study of the lysis-lysogeny decision in bacteriophage lambda became a paradigm for the genetic switch [15, 16], and in the time since then those ideas have been generalized, realized, and exploited across biology.

The repressor-operator model of Jacob and Monod provided not only a successful conceptual vision for gene expression writ large, but also served as the basis of mathematical models of transcription based upon the precepts of statistical mechanics [21–23]. These models provided a quantitative description of a variety of different regulatory contexts in which the strengths of binding sites, the repressor copy number and DNA loop length were altered, illustrating how genetic circuits could be tuned directly and quantitatively [24–31]. Interestingly, these pioneering studies became a jumping off point for the construction of a number of synthetic variants that when combined with fluorescent reporters made it possible to watch synthetic switches and oscillators in real time in single cells [32–34].

In addition to the seminal discoveries of the existence of gene circuits themselves, a parallel set of discoveries unfolded which added a second layer of regulatory control to the original repressor-operator model and its subsequent generalizations and elaborations. Specifically, the mystery of induction required another insight into biological feedback and control. Enzymatic adaptation, the idea that somehow enzymes that were latent would become active in the presence of the right substrate [6], led to the discovery of allostery, a concept that Monod himself referred to as the “second secret of life” [35]. In the context of gene regulation, this idea implies that transcription factors themselves are subject to control through the binding of effector molecules that alter their activity [36–45]. Writ large, these insights now fall under the general heading of allosteric transitions, a phenomenon in which proteins of all types undergo conformational changes that alter their activity. This idea applies broadly to ion channels, enzymes, the respiratory protein hemoglobin, membrane receptors mediating chemotaxis and quorum sensing, and of course, to the main subject of our paper, transcription factors [43].

**Figure 2:**
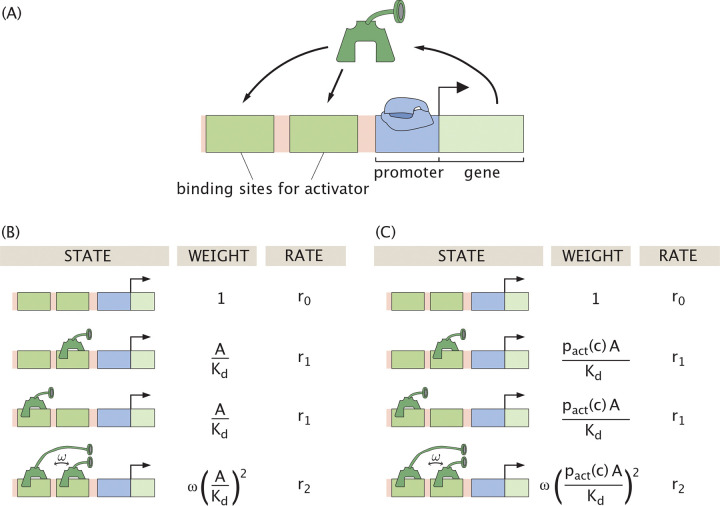
The auto-activation regulatory circuit. (A) Schematic of the operation of the circuit. Polymerase binding at the promoter (blue) transcribes the gene (encoded in the light green region), producing a protein that can activate its own expression at a sufficient concentration. In our model, an activator can bind at one of two possible sites to enhance gene transcription. (B) Thermodynamic states, weights, and rates for the circuit in the traditional model without induction. The parameter ω denotes the cooperative strength of two activators binding. (C) Thermodynamic states, weights and rates for the case in which the effector tunes the fraction of active activators. Note that in both of these cases the parameters $K_{d}$, ω, $r_{0}$, $r_{1}$ and $r_{2}$ are *effective* parameters that have hidden dependence upon the number of polymerases and the strength with which it binds the promoter. The explicit definitions of these effective parameters are worked out in Appendix A.

The mathematical analysis of genetic circuits is its own fascinating enterprise, using the tools of dynamical systems to explore the stability of switches and oscillations [46–50]. The idea for describing some circuit involving n different proteins is to write dynamical equations of the form

(1) $$\frac{d\left[TF_{i}\right]}{dt}=f_{i}\left(\left\{\left[TF_{j}\right]\right\}\right),$$

where there is one such equation for each transcription factor (for which we will often use the shorthand notation). The function on the right side acknowledges that the dynamics of the *i*^*th*^ TF can depend upon the concentrations of all the others, represented here by the notation $\left\{TF_{j}\right\}$ signifying “the set of all n TFs.” Perhaps the simplest such example we will discuss as our first case study is the auto-activation switch as shown in Fig. 2 and described by an equation of the form

(2) $$\frac{dA}{dt}=-\gamma A+\frac{r_{0}\;+\;r_{1}2\frac{A}{K_{d}}\;+\;r_{2}\omega {\left(\frac{A}{K_{d}}\right)}^{2}}{1\;+\;2\frac{A}{K_{d}}\;+\;\omega {\left(\frac{A}{K_{d}}\right)}^{2}},$$

where A is the number of activators, $K_{d}$ is the dissociation constant for A in its interaction with DNA, and ω is the cooperativity between two activators bound to the gene promoter along the DNA. The first term on the right captures the degradation of activator at rate γ, and the second term characterizes protein production with a basal level of production $r_{0}$ and a saturating level $r_{2}$.

The production rate in Eqn. 2, and throughout this work, is modeled using a thermodynamic framework relating promoter occupancy to output [23, 27, 51–55]. We note that often instead of adopting the full thermodynamic model to treat promoter occupancy, it is convenient to use Hill functions as an approximation to describe the probability of promoter binding [47–50, 56–60]. The auto-activation example will serve as our first foray into the problem of induction of genetic circuits by effector molecules as well as an opportunity to bring some critical scrutiny to the use of Hill functions to describe the physics of occupancy.

**Figure 3:**
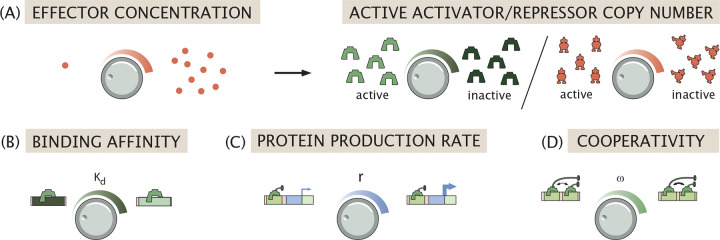
Tuning genetic circuits. The schematic shows different knobs which are available to the cell, the theorist and the experimentalist, namely (A) effector concentration (and by consequence the number of active activator or repressor molecules), (B) binding affinity $K_{d}$, (C) protein production rate r, and (D) cooperativity ω.

Typically, the exploration of the stability behavior of these circuits is based upon varying theoretically accessible parameters such as dissociation constants $\left(K_{d}\right)$, transcription rates $\left(r_{i}\right)$ and degradation rates $\left(\gamma \right)$ as featured in Eqn. 2 and in Fig. 3(B–D), without reference to how such parameters are themselves controlled by living cells [43]. In fact, often the underlying response is dictated by the presence and absence of experimentally accessible effector molecules that alter the balance between inactive and active forms of key regulatory molecules such as transcription factors, as shown in Fig. 3(A). Thus, while all of the tunable parameters in Fig. 3 make it possible to systematically tune the level of gene expression, some of these parameters are more conveniently accessible to the experimentalist and to the cell itself as it rapidly tunes its behavior in response to stimuli. Our goal is to use explicit statistical mechanical models of the induction phenomenon to explore the behavior of genetic circuits as a function of the presence or absence of effectors.

In the next few sections, we work our way through an array of increasingly sophisticated gene regulatory circuits and leverage the statistical mechanical framework to uncover how the presence of effectors dictates complex gene expression dynamics. We envision that the predictions and systematic analysis stemming from our work will make it possible to better understand how cells exploit these genetic circuits to regulate their decision making processes, as well as enable the predictive design of synthetic circuits with prescribed functions in response to input effector dynamics.

## II. THE STATISTICAL MECHANICS OF INDUCTION

We now undertake a systematic analysis of a number of different regulatory circuits from an allosteric perspective, building upon earlier work in which biological parameters are tuned by hand rather than by effectors [32, 34, 47, 48, 61, 62]. Although some previous studies also vary inducer concentration, they typically either address different regulatory contexts than ours, map inducer concentrations to protein activity without modeling the allosteric transition explicitly, or consider different types of dynamical systems analyses than we do [6, 10, 29, 30, 34, 57, 60, 63–68].

In particular, we argue that often the number of active transcription factors $TF_{\text{act}}$ is given by

(3) $$TF_{\text{act}}=p_{\text{act}}(c)TF_{\text{tot}},$$

where $TF_{\text{tot}}$ is the total number of transcription factors and $p_{\text{act}}(c)$ is the probability that the transcription factor is active as a function of the effector concentration c. For example, effector binding can render a protein inactive, decreasing $p_{\text{act}}$ as effector concentration c increases. We will show that the fraction of transcription factors that are active can be given by the Monod-Wyman-Changeux (MWC) model, which can be used to compute $p_{\text{act}}(c)$ using statistical mechanics [36–44]. Note that while we invoke the MWC model to describe allostery, we could just as well use the KNF model or even the phenomenological Hill functions to capture the role of the effector [43, 44]. The KNF model, for example, would describe effector binding as sequential and local. In this case, transcription factor structure would change at the subunit level, rather than through a single coordinated change in the protein’s quartenary conformation as described by the MWC interpretation [61, 69].

**Figure 4:**
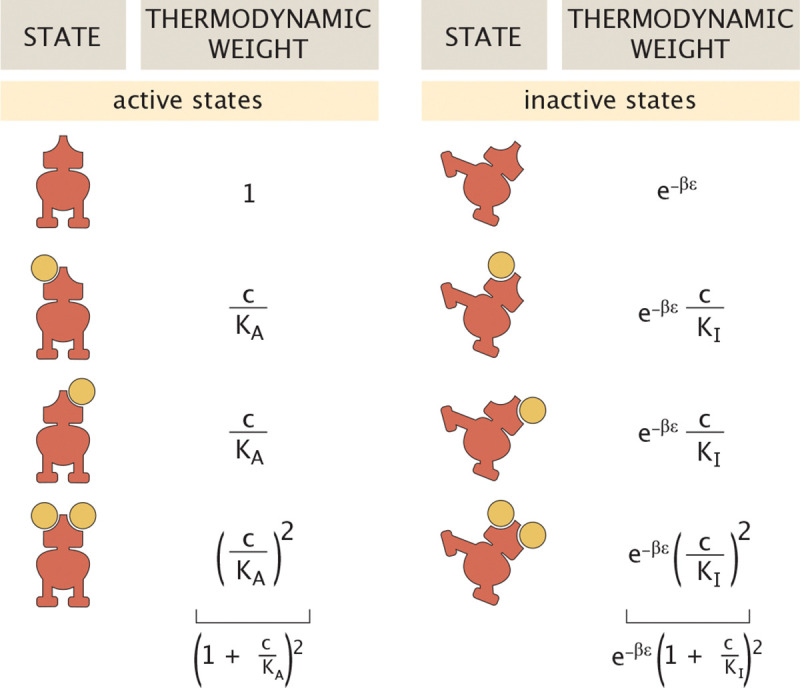
States and weights for an allosteric transcription factor with an effector that can bind at two sites on the protein. Effectors can bind to both the active and inactive forms of the transcription factor with different dissociation constants $K_{A}$ and $K_{I}$, which determine whether the effector stabilizes the protein more strongly in its active or inactive configuration. The sum of the thermodynamic weights for the active and inactive conformations are shown at the bottom.

In the MWC model, we consider an inactive state and an active state of the transcription factor with energy difference $\varepsilon =\varepsilon_{i}-\varepsilon_{a}$. In this setting, an effector can bind to a transcription factor in both its active and inactive forms with different dissociation coefficients. The values of these constants then determine whether increasing effector concentration stabilizes the active or inactive form of the transcription factor. The states and weights for such an allosteric transcription factor with two binding sites is shown in Fig. 4. Appealing to these states and weights, the probability of a transcription factor being active is then of the form

(4) $$p_{\text{act}}(c)=\frac{{\left(1\;+\;\frac{c}{K_{A}}\right)}^{2}}{{\left(1\;+\;\frac{c}{K_{A}}\right)}^{2}\;+\;e^{-\beta \varepsilon }{\left(1\;+\;\frac{c}{K_{I}}\right)}^{2}},$$

where c is the concentration of effector molecules. Here we define $\beta =1/k_{B}T$, and $K_{A}$ and $K_{I}$ as the dissociation constants for the transcription factor in its active and inactive states, respectively.

**Figure 5:**
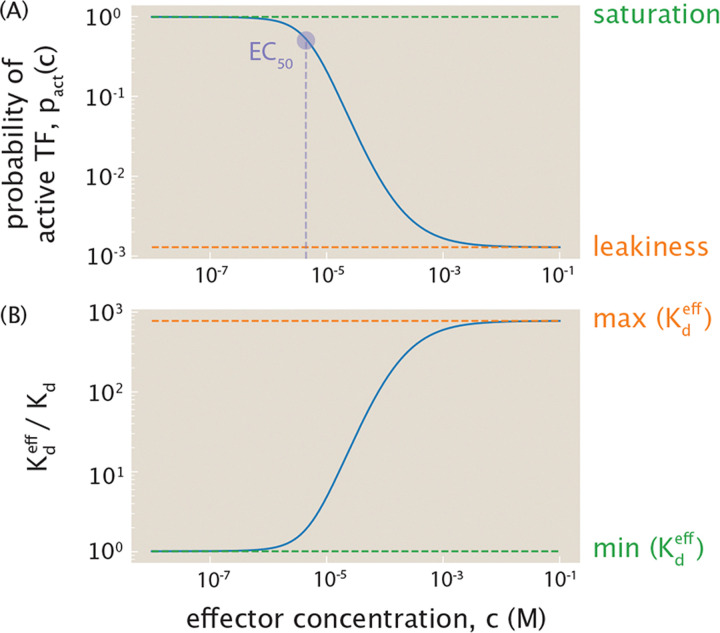
Activity of a transcription factor as a function of effector concentration c. Parameters used throughout the paper, unless otherwise stated, are: $K_{A}=140\;\mu M$, $K_{I}=530\;nM$, $\varepsilon =4.5\;k_{B}T$. (A) Probability of active transcription factor as a function of effector concentration, defined by Eqn. 4. The half maximal effective concentration EC_50_, defined as the effector concentration $c^{*}$ such that $p_{\text{act}}\left(c^{*}\right)=\left(p_{\text{act}}^{max}+p_{\text{act}}^{min}\right)/2$, is plotted in purple. (B) The effective dissociation constant $K_{d}^{\text{eff}}$ (dimensionless with respect to $K_{d}$) as a function of effector concentration. Saturation of $p_{\text{act}}(c)$ corresponds to minimal $K_{d}^{\text{eff}}$, and leakiness of $p_{\text{act}}(c)$ corresponds to maximal $K_{d}^{\text{eff}}$.

An alternative way of thinking about this approach is to express an effective dissociation constant $K_{d}^{\text{eff}}$ between transcription factors and DNA as

(5) $$K_{d}^{\text{eff}}=K_{d}/p_{\text{act}}(c),$$

where $K_{d}$ is the fixed physical dissociation constant and $p_{\text{act}}(c)$ modulates the activity of that transcription factor in an effector-concentration-dependent way. For a transcription factor with two effector binding sites, we can write the probability of being in the active state in the form given by Eqn. 4. The activity of the transcription factor as a function of effector concentration is shown in Fig. 5(A). This input-output function has the typical sigmoidal behavior of $p_{\text{act}}(c)$. It is worth noting that here we show the behavior of a transcription factor for which the effector renders the proteins inactive. By tuning the relative values of the active and inactive dissociation constants, however, we can also generate situations in which the activity of the transcription factor increases with effector concentration.

Writing $K_{d}^{\text{eff}}=K_{d}/p_{\text{act}}(c)$ in gene regulatory network motifs is useful and informative in many ways. First, traditionally, theoretical models of the behavior of network motifs are studied by tuning $K_{d}^{\text{eff}}$, while experiments generally tune the effector concentration c. Incorporating $p_{\text{act}}(c)$ bridges theory and experiments by providing experimentally accessible “knobs” to control the behavior of the genetic circuit of interest. Second, since $p_{\text{act}}(c)$ is highly nonlinear in c, other model variables might react differently to varying c than to varying $K_{d}^{\text{eff}}$. Third, $p_{\text{act}}(c)$ constrains the range of accessible $K_{d}^{\text{eff}}$ values. When discussing input-output curves, there are a variety of summary parameters that help us understand their character qualitatively. For example, in the case where effector binding renders the protein inactive, the leakiness is the amount of activity at saturating concentrations of effector, namely, $p_{\text{act}}(\infty )$. Similarly, the maximal activity known as the saturation occurs in the zero-effector limit, namely, $p_{\text{act}}(0)$. These important summary variables can be calculated as

(6) $$p_{\text{act}}^{\max}=\underset{c\to 0}{\lim}\;p_{\text{act}}=\frac{1}{1\;+\;e^{-\beta \varepsilon }}$$

and

(7) $$p_{\text{act}}^{\min}=\underset{c\to \infty }{\lim}\;p_{\text{act}}=\frac{1}{1\;+\;e^{-\beta \varepsilon }{\bar{K}}_{c}^{2}},$$

where ${\bar{K}}_{c}=K_{A}/K_{I}$. With the parameters used in Fig. 5(A), $p_{\text{act}}^{\min}$ and $p_{\text{act}}^{\max}$ are separated by about three orders of magnitude, and thus $K_{d}^{\text{eff}}$ is also only tunable across three orders of magnitude, as shown in Fig. 5(B).

**Figure 6:**
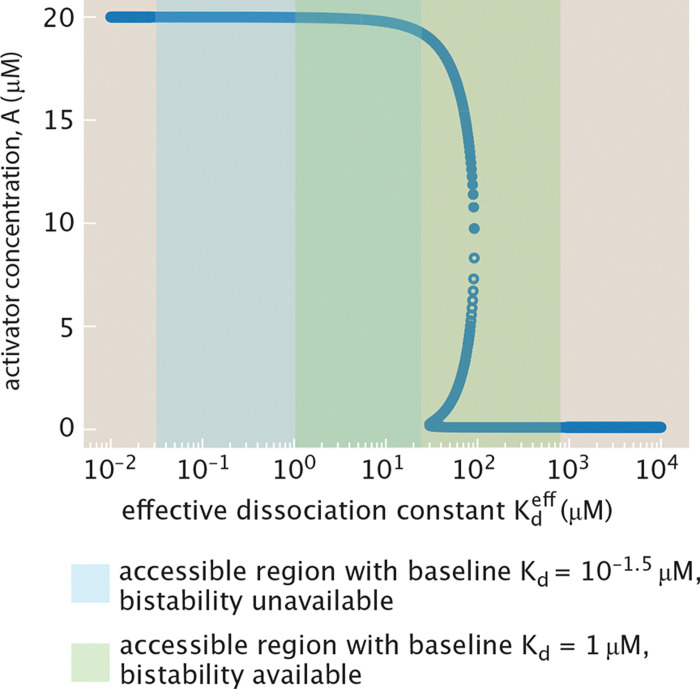
Allosteric tuning restricts the range of accessible $K_{d}^{\text{eff}}$. The dark blue curve shows the steady-state values of activator concentration A, with filled circles indicating stable fixed points and open circles indicating unstable ones. The light blue and light green shaded regions represent two example ranges of accessible $K_{d}^{\text{eff}}=K_{d}/p_{\text{act}}(c)$ values, obtained by varying the effector concentration c for two proteins with different DNA binding affinities (and thus different $K_{d}$ values). For the blue region, $K_{d}=3.2\times 10^{-2}\;\mu \text{M}=10^{-1.5}\;\mu \text{M}$; for the green region, $K_{d}=1\;\mu \text{M}$. The parameters for the auto-activation stability curve are: γ=1/min, $r_{0}=0.1\;\mu \text{M}/\min$, $r_{1}=1\;\mu \text{M}/\min$, $r_{2}=20\;\mu \text{M}/\min$, ω=100.

The restricted $K_{d}^{\text{eff}}$ range can have important consequences. For example, consider a bistable system with a stability curve such as that shown in Fig. 6, where the x-axis is $K_{d}^{\text{eff}}$ and the y-axis tracks the steady state concentration of some protein A. Tuning the parameter c imposes constraints on the range of achievable $K_{d}^{\text{eff}}$ for a given $K_{d}$. The light blue range shown does not intersect the bistable regime, while the light green range does, illustrating that bistability may not be fully accessible due to the functional dependence of $K_{d}^{\text{eff}}$ on $p_{\text{act}}(c)$. This restriction on $K_{d}$ follows a tradition of relating thermodynamic binding interactions to experimentally measured biochemical parameters [70].

**Figure 7:**
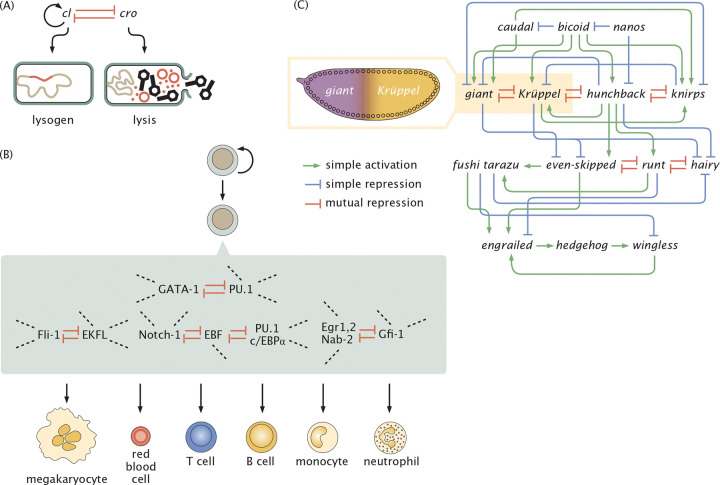
Mutual repression is ubiquitous in cellular decision making. (A) The bacteriophage lambda switch that mediates the phage decision to become a lysogen in the bacterium or engage in lysis through mutual repression of cI and cro (and some auto-activation of cI as well). (B) In hematopoietic development, mutual repression between different genes have been suggested to ensure the switch-like adoption of alternate cellular fates. Diagram adapted from [74] and [75]. (C) Mutual repression in the early fruit fly gene network has been associated with the emergence of discrete domains of gene expression. Diagram adapted from [76] and [77].

Our fundamental goal is to reconsider the classic stability analysis for a broad array of different regulatory architectures in light of the MWC model for transcription factor activity described above. For example, as seen in Fig. 6, tuning the $K_{d}$ for the simple auto-activation circuit yields two stable fixed points. In the first section, we examine this circuit by modulating the concentration of effector molecules, which tunes the concentration of active transcription factors. From this simple genetic circuit, we then turn to the ubiquitous mutual repression switch, well known not only as a key part of the repertoire of natural genetic circuits as shown in Fig. 7, but also as one of the classic examples of synthetic biology [32]. Both auto-activation and mutual repression can exhibit bistable dynamics [32, 47, 61, 62]. We examine the conditions for bistability as well as the relaxation dynamics in each. Finally, we turn to three-gene feed-forward loops, in which an input gene regulates expression of another both directly and indirectly through regulation of an intermediary. Depending on the precise architecture, these circuits exhibit unique time-varying behavior in response to pulsing effector signals [71–73].

## iii. BISTABILITY IN GENETIC CIRCUITS

Different gene networks serve different biological functions. Among the most important classes of networks are those that yield bistability. Bistability refers to the situation in which, for a given set of parameters, a system can exist in one of two possible stable steady states. Such a feature is biologically significant. For example, often the expression level of one protein determines a cell’s fate. To obtain cells with different functions, there might be some cells with high concentrations of the protein of interest and some cells with low concentrations, requiring a bistable system regulating the protein in question. Broad computational surveys have shown that switch-like, bistable behavior can emerge from a wide range of simple biochemical network architectures [78]. Here, we analyze the two simplest and most ubiquitous gene circuits that produce bistability [49], auto-activation and mutual repression, now through the new lens of how effector molecules modulate the dissociation constants of regulatory proteins binding to DNA. Although there are examples in the literature that also use this knob in other regulatory or modeling contexts (as noted in Section II), the approach considered here offers a contrast to the conventional setting often employed in studying auto-activation and mutual repression. In such cases, the tuning strategy is typically to modulate the binding parameters within thermodynamic models [47–49, 61], rather than those parameters being naturally tuned through the action of signaling molecules. For simplicity, the following sections do not consider transcription factor oligomerization, either in solution or when bound to DNA, which could give rise to more complex configurations such as DNA looping. We discuss modest generalizations to this effect in Appendix G.

### A. The auto-activation regulatory motif

Auto-activation circuits, in which a gene product enhances its own transcription, are among the simplest genetic regulatory motifs and are capable of generating bistable behavior [47]. Such motifs have been studied extensively in both synthetic and natural biological systems. In vitro synthetic networks have demonstrated robust switching between high and low expression states under controlled biochemical conditions [79], and positive feedback loops have been implicated in natural processes like cell differentiation, where they convert graded input signals into binary gene expression responses [80]. The inducible auto-activation circuit has also been explored with Hill function formulations and with $p_{act}$ as an input parameter [81]. Motivated by this work, we now present our theoretical analysis of the auto-activation switch that incorporates a full thermodynamic model and explicit treatment of induction. The architecture we study is shown schematically in Fig. 2, where a transcription factor activates its own production, forming a feedback loop. Given the states, weights, and rates in Fig. 2, we can now write the kinetic equation governing the dynamics of the auto-activation system as

(8) $$\frac{dA}{dt}=-\gamma A\;+\;\frac{r_{0}\;+\;r_{1}(2p_{\text{act}}(c)\frac{A}{K_{d}})\;+\;r_{2}\omega {\left(p_{\text{act}}(c)\frac{A}{K_{d}}\right)}^{2}}{1\;+\;2p_{\text{act}}(c)\frac{A}{K_{d}}\;+\;\omega {\left(p_{\text{act}}(c)\frac{A}{K_{d}}\right)}^{2}},$$

where γ is the degradation rate of protein A, $r_{i}$ is the production rate when i activators are bound, ω is the cooperativity of activator binding, and $K_{d}$ is the biophysical dissociation constant specific to a gene and a transcription factor. The effect of allosteric regulation is included in $p_{\text{act}}(c)$, as we introduced earlier. This probability modifies the active transcription factor concentration from A to $p_{\text{act}}(c)\;A$.

Note that we could alternatively describe this auto-activation switch by explicitly considering all possible regulatory states with bound and unbound RNA polymerase (RNAP), and explicitly defining an energy for interaction between activators and RNAP. Appendix A1 demonstrates, however, that this representation is equivalent to Eqn. 8, with the rates $r_{i}$, dissociation constant $K_{d}$, and cooperativity ω implicitly dependent on polymerase concentration, the strength of polymerase binding to the DNA, and the strength of interaction between polymerase and activator. The discussion throughout this paper will thus remain in the equivalent coarse-grained realm, as depicted in Fig. 2, and effectively focus on polymerase-bound states.

It is helpful to write our dynamical equation in dimensionless form. To do so, we non-dimensionalize the system by using 1/γ as the unit of time and $K_{d}$ as the unit of concentration. Within the $p_{\text{act}}$ paradigm, we can write the dynamical equation in dimensionless form as

(9) $$\frac{d\bar{A}}{d\bar{t}}=-\bar{A}\;+\;\frac{{\bar{r}}_{0}\;+\;{\bar{r}}_{1}\left(2p_{\text{act}}\bar{A}\right)\;+\;{\bar{r}}_{2}\omega {\left(p_{\text{act}}\bar{A}\right)}^{2}}{1\;+\;2p_{\text{act}}\bar{A}\;+\;\omega {\left(p_{\text{act}}\bar{A}\right)}^{2}},$$

where $\bar{t}=\gamma t$, $\bar{A}=A/K_{d}$ and ${\bar{r}}_{i}=r_{i}/\gamma K_{d}$.

**Figure 8:**
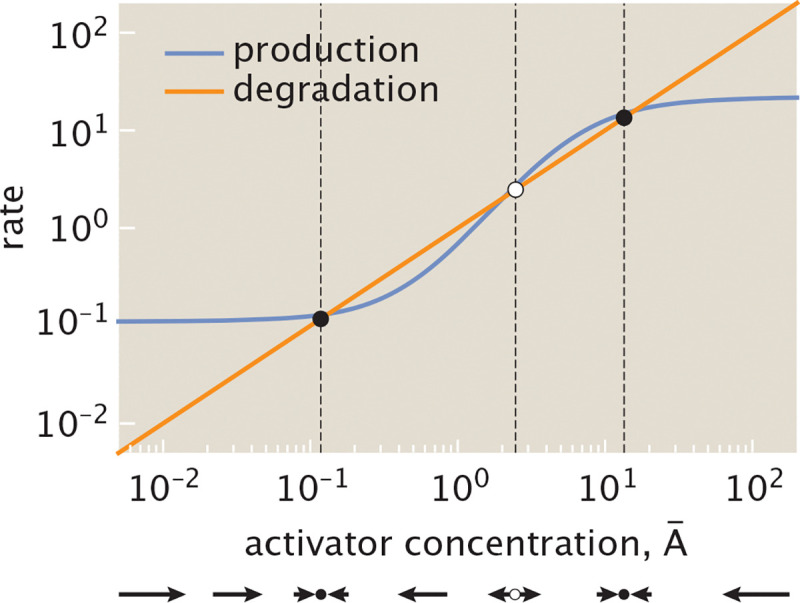
Plot of production and degradation rates for an auto-activation switch as a function of activator concentration. This figure illustrates the competition between the production and degradation terms for a system with rate constants ${\bar{r}}_{0}=0.1$, ${\bar{r}}_{1}=1$, ${\bar{r}}_{2}=20$, and cooperativity ω=10 at effector concentration c=25μM. Intersections of the curve denote stable (filled nodes at low concentration ${\bar{A}}_{\text{low}}$ and high concentration ${\bar{A}}_{\text{high}}$) and unstable (unfilled node ${\bar{A}}_{\text{unstable}}$) fixed points. The vectors denote a phase portrait representing the direction and magnitude of change in activator concentration as a function of activator concentration itself.

At a given effector concentration, we can represent the gene expression dynamics that unfold through a phase portrait as in Fig. 8. The points of intersection of the production and degradation curves correspond to steady state ac**t**ivator concentrations. Fig. 8 highlights a system that can stabilize to one of two possible states with a high $\left({\bar{A}}_{\text{high}}\right)$ or low $\left({\bar{A}}_{\text{low}}\right)$ activator concentration. Depending on the initial concentration of activator protein, the system will converge to one of these stable points. At the unstable steady state ${\bar{A}}_{\text{unstable}}$, only a small perturbation is needed for the system to evolve toward one of the two stable steady states.

**Figure 9:**
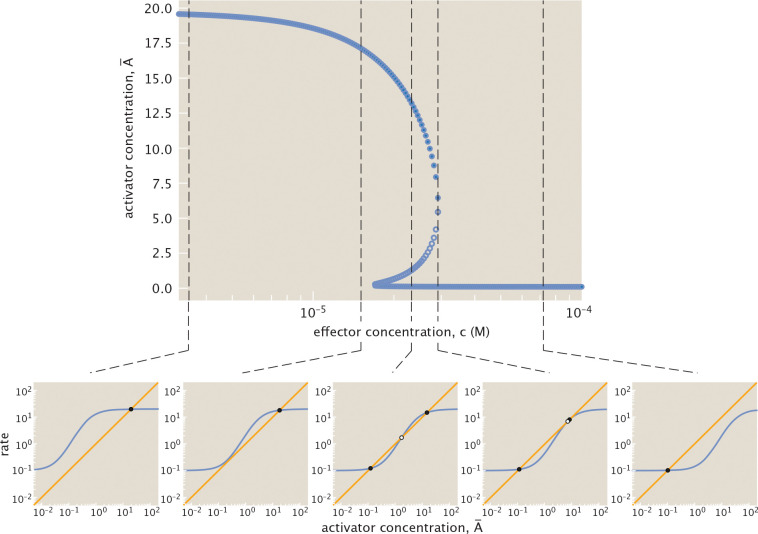
Bifurcation diagram for auto-activation system with rate constants ${\bar{r}}_{0}=0.1$, ${\bar{r}}_{1}=1$, ${\bar{r}}_{2}=20$, and cooperativity ω=10. This curve plots all stable (filled nodes) and unstable (unfilled nodes) fixed points, demonstrating a shift at intermediate effector concentrations from one to three fixed points. Each black dashed line denotes a specific effector concentration, pointing to a plot of the corresponding production (blue) and degradation (orange) rates as a function of activator concentration. Curve intersections mark the stable (filled nodes) and unstable (unfilled nodes) fixed points found at the given effector concentration.

We can now qualitatively visualize how the dynamics of auto-activation transform at different effector concentrations. Specifically, as we will see explicitly in the next section, for each effector concentration c we generate a phase portrait analogous to that shown in Fig. 8. We then determine the number of stable and unstable fixed points and their corresponding activator concentrations. Performing this analysis as we tune effector concentration yields the bifurcation curve shown in Fig. 9. At a low effector concentration, activators are more likely to be found in their active configurations, enhancing gene expression such that the system always stabilizes to a state with a high concentration of activator. The magnitude of this concentration approaches a maximal value defined by the rate ${\bar{r}}_{2}$ for activated protein production. As the effector concentration increases, the system becomes bistable, allowing a bimodal distribution in protein concentrations for an ensemble of cells [62].

Ultimately, at sufficiently high effector concentration, activators are sequestered in their inactive configuration such that the system can only stabilize to a state with low activator concentration. The magnitude of activator expressed is then largely defined by the basal rate of production without bound activator, i.e., ${\bar{r}}_{0}$. Tracking the system’s corresponding production and degradation curves through a series of snapshots in Fig. 9, we observe that these shifts between bistable and monostable dynamics emerge because the increasing effector concentration shifts the system production curve toward higher activator concentration. Expressed differently, as effector concentration increases, a higher activator concentration is necessary to achieve a given production rate.

**Figure 10:**
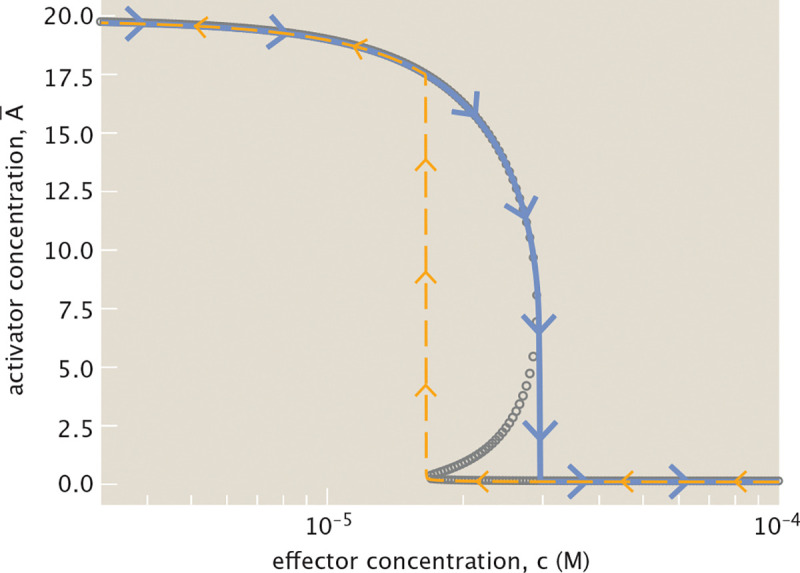
Plot of stable state evolution, exhibiting hysteresis under different trends for effector concentration. The blue curve plots how the stable state evolves with increasing effector concentration from an initial high concentration of activator. The orange dashed curve plots the change in stable activator concentration starting from an initially low level as effector concentration decreases. Comparing to the bifurcation diagram (gray) previously shown in Fig. 9, the history of steady-state evolution determines the threshold concentration of effector at which the system switches state, highlighting hysteretic behavior.

Note that the threshold at which the system switches from one stable state to another differs when increasing and decreasing the effector concentration. If the system initially contains a high concentration of activator before tuning, a higher concentration of effector is necessary to switch to the low activator state than is required when decreasing effector concentration for a system with initially low activator concentration. Considering Fig. 9 again, the higher threshold corresponds to the maximum effector concentration at which the system is bistable, and the lower threshold to the minimum concentration at which the system is bistable. Fig. 10 illustrates this phenomenon of hysteresis more explicitly, overlaying the previously discussed bifurcation diagram, and showing how the threshold at which the blue stable equilibrium trajectory switches from high to low activator concentration differs from the threshold for the orange trajectory tracing the switch in reverse.

We are particularly interested in characterizing the conditions for bistability to occur at effector concentrations c, as well as how the bistable regime (if it exists) responds to changes in parameters such as production rates and cooperativity. While previous studies have analyzed bistability in auto-activation circuits using thermodynamic models without effectors [47, 82], our aim is to extend this to include effector dependence explicitly and systematically explore how the bistable region evolves across a broader parameter space.

#### 1. Regimes of bistability

We now analyze the ways in which bistability emerges in the context of effector-mediated genes. One could begin with simply a fixed set of parameters and asking whether the system is bistable. In other words, can the concentration of gene product A settle at either a high or low steady state depending on the initial condition, thereby functioning as a binary switch?

The question becomes more nuanced, however, when taking the role of effector molecules into account. Some parameters are more intrinsic and less readily tunable than others. For instance, molecular constants such as binding affinities or cooperativity are typically encoded in the system’s molecular architecture. In contrast, cells can regulate the concentration of effector molecules relatively easily and rapidly, either through controlled expression, import/export mechanisms, or degradation pathways. Given this, the most relevant questions to ask are (*i*) under what constraints on the system’s intrinsic parameters can bistability be achieved for at least some range of effector concentration c, and (*ii*) how wide is that range? Indeed, even if the effector concentration is a more accessible control knob, the cell is unlikely to operate at a single precise value, if only due to molecular noise and environmental fluctuations. It is therefore biologically relevant to assess not just whether bistability is possible, but whether it is robust to such fluctuations in effector levels.

**Figure 11:**
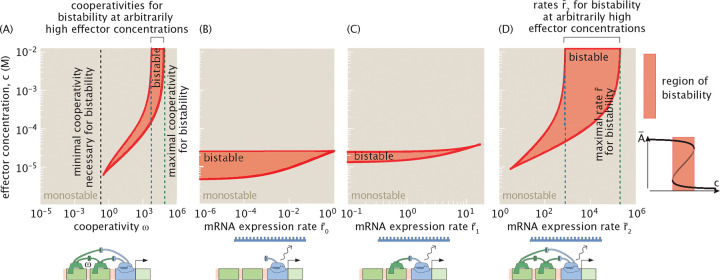
Range of effector concentrations for which the system exhibits bistability. The red shaded region indicates bistability. Outside this region, the system is monostable. Unless otherwise specified, parameters are fixed at: ω=7.5, ${\bar{r}}_{0}=0.1$, ${\bar{r}}_{1}=1$, and ${\bar{r}}_{2}=20$. (A) Bistable concentration range as a function of cooperativity ω, varied over the interval $\omega \in \left[10^{-6},10^{6}\right]$, corresponding to interaction energies between the two activators from approximately +14, $k_{B}T$ to −14, $k_{B}T$, since $\omega =e^{{-\beta \varepsilon }_{\text{int}}}$. The dotted line shows the analytical lower bound on the minimal cooperativity required for bistability. (B) Bistable concentration range as a function of ${\bar{r}}_{0}$, sampled over the interval ${\bar{r}}_{0}\in \left[10^{-6},{\bar{r}}_{1}\right]$. (C) Bistable concentration range as a function of ${\bar{r}}_{1}$, sampled over the interval ${\bar{r}}_{1}\in \left[{\bar{r}}_{0},{\bar{r}}_{2}\right]$. (D) Bistable concentration range as a function of ${\bar{r}}_{2}$, sampled over the interval ${\bar{r}}_{2}\in \left[{\bar{r}}_{1},10^{6}\right]$. The rate parameters are varied under the constraint of the auto-activation condition ${\bar{r}}_{0}\leq {\bar{r}}_{1}\leq {\bar{r}}_{2}$, which ensures that the production rate increases with the number of bound activators.

We observe that even under the idealized assumption that the cell could freely choose its effector concentration, bistable behavior arises only within a restricted range of parameter values. This is shown numerically in the red regions of Fig. 11. To investigate the conditions under which multiple steady states are possible, we also derive analytical bounds in parameter space in Appendix B 1. By setting $d\bar{A}/d\bar{t}=0$ and rewriting the resulting expression in standard polynomial form, we can infer the number of positive real roots using classical results (i.e., Descartes’ rule of signs) that relate sign changes in the coefficients of a polynomial to the number of positive roots. This leads to a necessary condition on the parameters of the system: for bistability to be possible for at least one value of the effector concentration, the system must satisfy the inequalities

(10) $$\frac{\omega {\bar{r}}_{2}}{2}>1\;+\;e^{-\beta \varepsilon },$$

(11) $$2{\bar{r}}_{1}<1\;+\;e^{-\beta \varepsilon }K_{c}^{2},$$

(12) $$\omega {\bar{r}}_{2}>4{\bar{r}}_{1}.$$

Notably, these conditions do not depend on ${\bar{r}}_{0}$.

In Fig. 11, we systematically modulate the four parameters of the auto-activation system: the strength of cooperative binding ω, the protein production rate ${\bar{r}}_{0}$ in the absence of activator binding, the protein production rate ${\bar{r}}_{1}$ when one activator is bound to the DNA, and the protein production rate ${\bar{r}}_{2}$ when two activators are bound. Fig. 11(A) shows the consequences of varying the cooperativity parameter ω, sampling values from 10^−6^ to 10^6^ to represent systems with positive cooperativity (ω>1), systems that are non cooperative (ω=1), and systems with negative cooperativity (ω<1). Recall that cooperativity describes the energy of interaction between two bound activators, $\varepsilon_{\text{int}}$, and therefore can be written as $\omega =e^{-\beta \varepsilon_{\text{int}}}$. The range of cooperativity shown in the figure thus corresponds to interaction energies ranging from $\varepsilon_{\text{int}}\approx -14k_{B}T$ to $\varepsilon_{\text{int}}\approx 14k_{B}T$, encompassing a broad and biologically relevant spectrum of interaction strengths [53, 54, 61].

Fig. 11(B), (C), and (D) explore the effects of varying the rates ${\bar{r}}_{0}$, ${\bar{r}}_{1}$ and ${\bar{r}}_{2}$, respectively. To do so, we impose constraints on these parameters to ensure that the system remains within the auto-activation regime, as defined in Appendix E. Specifically, we require that the production term in Eqn. 9 remains a monotonically increasing function of $\bar{A}$, such that $\bar{A}$ consistently acts as an activator across all concentrations. This condition imposes the inequality ${\bar{r}}_{0}\leq {\bar{r}}_{1}\leq {\bar{r}}_{2}$, which we enforce throughout our analysis when varying ${\bar{r}}_{0}$, ${\bar{r}}_{1}$ and ${\bar{r}}_{2}$.

We note that the necessary criteria for bistability derived in Eqns. 10 – 12 do not impose any effective constraint when tuning either ${\bar{r}}_{0}$, ${\bar{r}}_{1}$, or ${\bar{r}}_{2}$ for the set of parameters chosen in Fig. 11. Specifically, the predicted lower bound on ${\bar{r}}_{1}$ exceeds the upper limit on ${\bar{r}}_{1}$ allowed by the auto-activation constraint (i.e., ${\bar{r}}_{1}\leq {\bar{r}}_{2}$). Similarly, the threshold for ${\bar{r}}_{2}$ above which bistability is possible lies below ${\bar{r}}_{1}$, meaning the system no longer behaves as a strictly auto-activating unit. Indeed, we observe that for parameter values consistent with the auto-activation regime, when either $\bar{r}$ or ${\bar{r}}_{2}$ is varied individually in Fig. 11(C) and (D), the system fails to exhibit bistability only in a narrow region where ${\bar{r}}_{1}$ approaches ${\bar{r}}_{2}$. Note, however, that this behavior is not universal. For different parameter values, the relative positioning of these thresholds may change, and the necessary conditions specified by Eqns. 10 – 12 could become more explicitly informative. The conclusions drawn here are therefore specific to the parameter set used in this analysis.

We show that the parameters ${\bar{r}}_{2}$ and ω play similar roles in shaping the system’s ability to exhibit bistability. As seen in Fig. 11(A), there exists a critical threshold of cooperativity ω below which the system is strictly monostable, indicating that a minimal level of nonlinearity is required for bistability. The analytical bounds derived in Eqns. 10 and 12 accurately capture this threshold. Likewise, Fig. 11(D) demonstrates that ${\bar{r}}_{2}$ must exceed a minimum value to support bistability; below this threshold, the system remains monostable for all effector concentrations.

Increasing ${\bar{r}}_{2}$ strengthens the contrast between states with high and low activator steady state concentrations in a positively cooperative system (ω>1), and as a result, the system becomes unstable for a broader range of effector concentrations. Therefore, as either ω or ${\bar{r}}_{2}$ increases, the region of effector concentrations that supports bistability broadens. This leads to a wider hysteresis zone and expands the range over which the system can toggle between high and low steady states under a fixed set of parameters. The region of bistability in effector concentration space is displaced toward higher concentrations, where the activation probability $p_{\text{act}}(c)$ approaches the leakiness limit. This reflects the fact that the effector destabilizes the activator by decreasing its effective DNA binding affinity. In this sense, increasing effector concentration counteracts the effect of high ω and ${\bar{r}}_{2}$, which tend to promote high expression levels of A. These two opposing effects—activation-driven amplification and effector-driven destabilization—create a balance that enables bistability.

Due to the system’s leakiness, complete inactivation of the activator is never achieved, even at high effector concentrations. As a result, for sufficiently large values of ω and ${\bar{r}}_{2}$, bistability can occur for all concentrations above a finite lower bound $c_{\min}(\omega ,{\bar{r}}_{0},{\bar{r}}_{1},{\bar{r}}_{2})$. This behavior corresponds to a limited region in parameter space where the system remains bistable at large c, as illustrated by the blue and green dotted lines in Fig. 11(A) and (C). In Appendices B 2 and D, we derive analytical bounds that predict the onset and breakdown of this upper-unbounded bistable regime.

Beyond a certain point and with fixed values of effector concentrations, further increases in either parameter have the opposite effect. When ω becomes too large, activators bind excessively tightly to the DNA, effectively locking the system into a high-expression state. Similarly, if ${\bar{r}}_{2}$ becomes too large, the system favors high levels of gene expression, and bistability is again lost (we analyze this transition using two-dimensional numerical sweeps and supporting analytical arguments in Appendix D). This behavior is a direct consequence of how the effector enters the model. If, instead of varying the effector concentration, we varied an effective dissociation constant $K_{d}^{\text{eff}}$, the bistable region would always remain bounded within a finite range of values.

This behavior contrasts with the effect of increasing ${\bar{r}}_{0}$ or ${\bar{r}}_{1}$. As shown in Fig. 11(B) and (C), higher values of either parameter reduce the width of the bistable region, and beyond a critical threshold, bistability disappears entirely. We can therefore infer that, in a positively cooperative system (ω>1), elevated values of ${\bar{r}}_{0}$ and ${\bar{r}}_{1}$ undermine the system’s ability to function as a bistable switch.

Interestingly, we observe bistability for values of the cooperativity parameter ω that are less than one, as seen in Fig. 11(A). This can be reconciled in two complementary ways. First, we can define an effective cooperativity for the system, given by $\omega_{\text{eff}}=\omega \cdot {\bar{r}}_{2}/2$. From the necessary conditions for bistability derived in Eqns. 10 and 12 we find that $\omega_{\text{eff}}>1$ is required for bistability. While this condition is necessary but not sufficient, it suggests that $\omega_{\text{eff}}$ captures the functional cooperativity of the system more accurately than ω alone, as it incorporates both the interaction between bound activators and the maximal rate of activator production. We can also reconcile our bistable results containing values of ω less than one by examining the effective Hill coefficient of the steady-state input–output function. As shown in Appendix H, bistability is observed only when the effective Hill coefficient exceeds one, as anticipated from theoretical considerations [83]. This reinforces the idea that the system can exhibit bistability even when ω<1, provided the overall nonlinearity—quantified either by $\omega_{\text{eff}}$ or the effective Hill coefficient—is sufficiently strong.

#### 2. Comparing Hill function and thermodynamic formulations

Thus far our paper has employed a thermodynamic formulation of the auto-activation switch, rooted in the principles of statistical mechanics. In previous discussions of gene circuits, however, a phenomenological Hill function is the predominant method to model these dynamics (e.g., [32, 33]). We note the fascinating origins of the Hill function in the work of Archibald Hill. More than 100 years ago, Hill wrote down a description of the binding between oxygen and hemoglobin that we now know as the Hill function, which he wrote as

(13) $$p_{\text{bound}}(x)=\frac{{\left(\frac{x}{K}\right)}^{n}}{1\;+\;{\left(\frac{x}{K}\right)}^{n}},$$

where x is the concentration of $O_{2}$ and K is its allied dissociation constant.

As Hill himself tells us, this functional form provides a summary of the occupancy of hemoglobin (the example he used, though it has been applied much more broadly). If we think of the huge topic of input-output functions in biology, then the kind of characteristics embodied in the Hill approach include a representation of leakiness (the amount of output even in the absence of input, $p_{\text{bound}}\;(0)$), dynamic range, $EC_{50}$ (the input concentration at which the output reaches half its maximum, $EC_{50}=K$) and the sensitivity as measured by the slope of the input-output curve (usually in logarithmic variables) at the midpoint. It is instructive to hear Hill commenting on his thinking: “My object was rather to see whether an equation *of this type* can satisfy all the observations, than to base any direct physical meaning on n and K [84].” He goes further in his 1913 paper noting [85] “In point of fact n does not turn out to be a whole number, but this is due simply to the fact that aggregation is not into one particular type of molecule, but rather into a whole series of different molecules: so that equation (1) (our Eqn. 13) is a rough mathematical expression for the sum of several similar quantities with n equal to 1, 2, 3, 4 and possibly higher integers.” We think it is important to remember that the Hill function is a phenomenological description of equilibrium binding that assumes certain states of occupancy are irrelevant, or alternatively, that bunches all of the states of occupancy into one effective non-integer state of occupancy [55].

In comparing and contrasting the two treatments of transcription factor binding, we find that they can yield different results. Hill functions approximate away some thermodynamic states and describe the dynamics as

(14) $$\frac{dA}{dt}=-\gamma A\;+\;\frac{r_{0}\;+\;r_{2}{\left(p_{\text{act}}(c)\frac{A}{K_{d}^{\text{eff}}}\right)}^{n}}{1\;+\;{\left(p_{\text{act}}(c)\frac{A}{K_{d}^{\text{eff}}}\right)}^{n}},$$

where n is the Hill coefficient and $K_{d}^{\text{eff}}$ is an effective dissociation constant. Appendix F demonstrates explicitly how this Hill function form derives from the high cooperativity limit of the thermodynamic model, removing the single activator-bound state as has been reported previously in the literature [55].

Reconciling the Hill and thermodynamic models for n=2 in the limit of large ω requires an effective dissociation constant of $K_{d}^{\text{eff}}=K_{d}/\sqrt{\omega }$, as discussed in Appendix F. Since this means that $K_{d}^{\text{eff}}\neq K_{d}$ when ω≠1, the natural concentration scales differ in the Hill and thermodynamic settings, and we must thus exercise caution in comparison. The remaining analysis of this subsection therefore employs the dimensional forms of each model to allow direct comparison.

**Figure 12:**
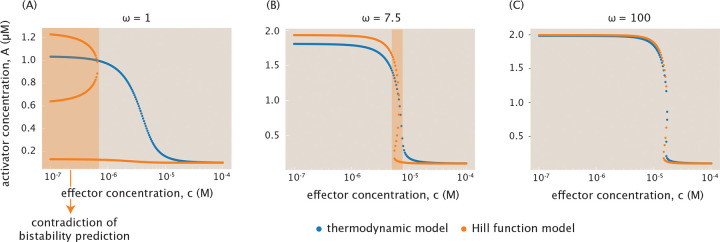
Bifurcation diagrams generated by the Hill function model and the thermodynamic model differ at small to intermediate cooperativity but converge at large cooperativity. The blue dots are fixed points in the thermodynamic model, and the orange dots are the fixed points in the Hill function model. The orange shaded region tracks where conflicting bistability predictions are made between the two models. The shared parameters are $r_{0}=0.1\mu \text{M}/\min$, $r_{2}=2\mu \text{M}/\min$, γ=1/min, $K_{d}=1\mu \text{M}$. The thermodynamic model has $r_{1}=1\mu \text{M}/\text{min}$ across the three panels. The cooperativities are (A) ω=1, (B) ω=7.5, (C) ω=100.

When cooperativity ω is small or moderate, the Hill function model can provide different predictions from the thermodynamic model. There are two ways to compare the models. One compares only the probability of state occupancy, as done in Appendix F, and the other compares the bifurcation curves of the full dynamical system when each production term is used. In the first case, the two models often yield similar end results, with their state occupancy probabilities agreeing to some degree even for small ω. In contrast, when the production terms are embedded in the dynamical system, the models can diverge substantially. Figure 12(A) shows this for the low cooperativity case with ω=1. Most notably, at low effector concentrations the Hill model shows bistability while the thermodynamic model does not. In the intermediate cooperativity regime of Fig. 12(B), the bifurcation diagrams resemble each other more closely. Nevertheless, they still make conflicting predictions for bistability in a range of effector concentration between 10^−6^ M to 10^−5^ M. In the high cooperativity regime of Fig. 12(C), the two models ultimately agree and produce identical bifurcation profiles.

Given the relatively light computational requirements for the model systems discussed here, as opposed to more complex and high-dimensional gene-interaction networks [86, 87], we will proceed with the thermodynamic formulation at high cooperativity for all other gene circuits considered in the paper. Since our analysis addresses the existence of bistability across all three cooperativity regimes, using the full thermodynamic model also ensures that the discussion captures all essential dynamical features across varying cooperativities.

#### 3. Timescale for stabilization

In addition to the question of steady states, it is interesting to examine the timescale for an inducible genetic circuit such as the auto-activation switch to reach steady state. In doing this analysis we remind the reader that our treatment assumes separation of time scales between the dynamics of effector binding and allosteric transitions, and the dynamics of the relaxation to steady state. As a result, the binding of effector and the allosteric state of the activator rapidly equilibrate as the activator concentration changes dynamically.

**Figure 13:**
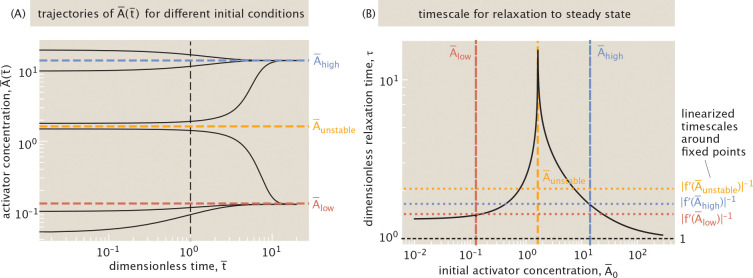
Dynamics of the auto-activation switch. The parameters of the system are fixed at: ω=7.5, ${\bar{r}}_{0}=0.1$, ${\bar{r}}_{1}=1$, ${\bar{r}}_{2}=20$, and $c=2\cdot 10^{-5}\;\text{M}$. (A) Time evolution of the activator concentration for various initial conditions. Each black curve represents a trajectory $\bar{A}(\bar{t})$ starting from a different initial condition ${\bar{A}}_{0}$. Dashed horizontal lines indicate the stable (${\bar{A}}_{\text{high}}$ and ${\bar{A}}_{\text{low}}$) and unstable $\left({\bar{A}}_{\text{unstable}}\right)$ fixed points. (B) Relaxation timescale obtained from exponential fits to the trajectory $\bar{A}(\bar{t})$ as a function of the initial concentration ${\bar{A}}_{0}$. Vertical lines indicate the positions of the steady states, while horizontal dashed lines mark the timescales associated with small perturbations around each fixed point, computed from the inverse slope ${\left|f^{\prime }(\bar{A})\right|}^{-1}$ of the function f defined in Eqn. 16. The solid horizontal black line corresponds to the reference timescale $\bar{t}=1$.

As shown in Fig. 13(A), the relaxation timescale depends strongly on the initial activator concentration ${\bar{A}}_{0}$. In particular, as seen in the figure, the relaxation timescale to steady state increases as the initial value of activator concentration, ${\bar{A}}_{0}$, gets closer to the unstable fixed point. The increased time to reach steady state near the unstable point reflects the typical behavior of positive feedback systems near bifurcations, where the system lingers near the threshold before switching [88]. However, for large initial concentrations of A, the dimensionless relaxation timescale approaches 1, as shown by the line at $\bar{t}=1$ in Fig. 13(A). This corresponds to the protein degradation timescale 1/γ, which serves as the unit of time in our non-dimensionalization. This also corresponds to the relaxation timescale of a simply regulated gene, activated solely by an upstream transcription factor. We find that auto-activation consistently delays the response compared to simple regulation, although the two converge in the high-A limit. Within the Hill-function framework, positive autoregulation has like-wise been shown to slow gene circuit response times [89], in contrast to the accelerating effect of negative feedback [90].

To further interpret these trends, we apply linear stability analysis to Eqn. 9, as is commonly done in the study of dynamical systems [91]. Linearizing around a point ${\bar{A}}_{i}$ with $\bar{A}(\bar{t})={\bar{A}}_{i}+\delta \bar{A}(\bar{t})$ and expanding the right-hand side, that we denote $f(\bar{A})$, to first order yields

(15) $$f(\bar{A}(t))\approx f\left({\bar{A}}_{i}\right)\;+\;f^{\prime }\left({\bar{A}}_{i}\right)\delta \bar{A}(t)$$

for sufficiently small $\delta \bar{A}(\bar{t})$, where $f^{\prime }(\bar{A})$ is a derivative with respect to $\bar{A}$ rather than a time derivative. The function f is given by

(16) $$f(\bar{A})=-\bar{A}\;+\;\frac{{\bar{r}}_{0}\;+\;{\bar{r}}_{1}\left(2p_{\text{act}}\bar{A}\right)\;+\;{\bar{r}}_{2}\omega {\left(p_{\text{act}}\bar{A}\right)}^{2}}{1\;+\;\left(2p_{\text{act}}\bar{A}\right)\;+\;\omega {\left(p_{\text{act}}\bar{A}\right)}^{2}}.$$

Therefore, the linearized equation governing the evolution of a small perturbation $\delta \bar{A}(t)$ around the point ${\bar{A}}_{i}$ becomes

(17) $$\frac{d\delta \bar{A}}{d\bar{t}}=f\left({\bar{A}}_{i}\right)\;+\;f^{\prime }\left({\bar{A}}_{i}\right)\delta \bar{A}.$$

If ${\bar{A}}_{i}$ is a steady state, then $f\left({\bar{A}}_{i}\right)=0$, and the equation reduces to exponential relaxation with dimensionless timescale ${\left|f^{\prime }\left({\bar{A}}_{i}\right)\right|}^{-1}$. Stable fixed points, denoted ${\bar{A}}_{\text{low}}$ and ${\bar{A}}_{\text{high}}$, satisfy $f^{\prime }\left({\bar{A}}_{\text{low}/\;\text{high}}\right)<0$, so perturbations decay over time. For unstable fixed points, where $f^{\prime }\left({\bar{A}}_{\text{unstable}}\right)>0$, small deviations grow exponentially and drive the system away from the fixed point.

However, if ${\bar{A}}_{i}$ is not a fixed point, then $f\left({\bar{A}}_{i}\right)\neq 0$ and the solution to Eqn. 17 does not represent convergence to a steady state. Instead, it predicts that $\delta \bar{A}$ asymptotes to a finite offset $f\left({\bar{A}}_{i}\right)/\left|f^{\prime }\left({\bar{A}}_{i}\right)\right|$, breaking the assumption of linearity $\left(\delta \bar{A}(\bar{t})\to 0\right)$. In this case, the derivative $f^{\prime }\left({\bar{A}}_{i}\right)$ still encodes the local response to small perturbations, but only describes the dynamics while deviations remain small. Therefore, the timescale ${\left|f^{\prime }(\bar{A})\right|}^{-1}$ is most meaningful in the vicinity of fixed points, even if it can still provide qualitative insights elsewhere.

These insights are reflected in Fig. 13(B), where we numerically compute the relaxation timescale as a function of the initial condition ${\bar{A}}_{0}$. For initial values near the stable fixed points, the timescale closely follows the linear prediction ${\left|f^{\prime }\left({\bar{A}}_{\text{low}/\;\text{high}}\right)\right|}^{-1}$. In contrast, when the initial condition lies near the unstable fixed point, the system initially diverges exponentially and only later relaxes nonlinearly to a stable state. This leads to an overall increase in the time to reach steady state, consistent with the trajectories displayed in Fig. 13(A) (see Appendix I for the precise numerical procedure used to compute these timescales). Together, these results reveal that the stabilization timescale in auto-activation circuits is not fixed but depends sensitively on initial conditions, particularly near unstable fixed points—highlighting the importance of considering nonlinear transient dynamics when predicting response times in bistable gene networks.

### B. The mutual repression regulatory motif

**Figure 14:**
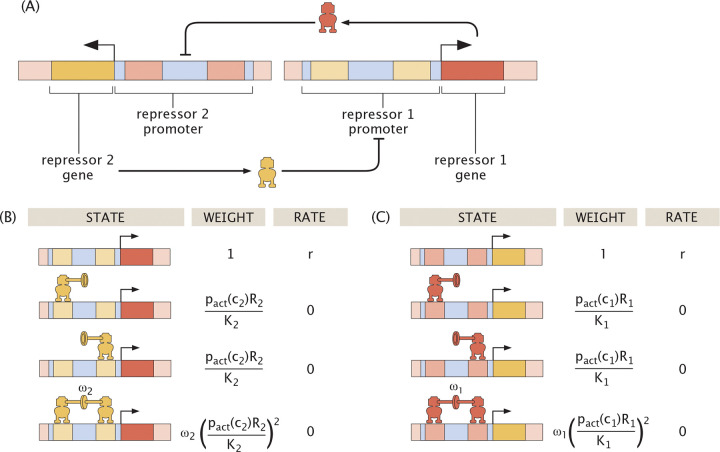
The mutual repression regulatory circuit. (A) Schematic of the operation of the circuit. When the gene for repressor 1 is expressed, the resulting protein downregulates the expression of the gene for repressor 2. Repressor 2, in turn, downregulates the expression of the gene for repressor 1. (B) Thermodynamic states, weights, and rates for expression of repressor 1. In our model, a repressor can bind non-exclusively at one of two possible sites within the target promoter region to suppress gene transcription. The parameter $\omega_{2}$ denotes the cooperative strength between two bound repressors $R_{2}$. (C) Thermodynamic states, weights, and rates for expression of repressor 2. The states and weights for the regulation of the promoter responsible for the production of repressor 2 are analogous to those shown for repressor 1. However, the dissociation constant of repressor 1 in this case is given by $K_{1}$, and the cooperativity term for the interaction of two repressor 1 molecules bound to the DNA is $\omega_{1}$.

Many regulatory circuits in prokaryotes and eukaryotes alike are mediated by the interaction between two genes that repress each other as shown in Fig. 14 [74, 75, 92]. Indeed, one of the signature achievements of the early days of synthetic biology was the consideration of a mutual repression switch in bacteria whose behavior was reported by the use of fluorescent proteins and controlled by a small molecule inducer [32]. As the name suggests, two genes $R_{1}$ and $R_{2}$ mutually repress each other. To simplify our analysis we assume that the two genes share the same degradation rate γ and production rate r.

By inspecting the states and weights diagrams of Fig. 14, we can express the dynamics of repressor 1 expression as

(18) $$\frac{\text{d}R_{1}}{\text{d}t}=-\gamma R_{1}\;+\;\frac{r}{1\;+\;2\frac{p_{\text{act}}\left(c_{2}\right)R_{2}}{K_{2}}\;+\;{\left(\frac{p_{\text{act}}\left(c_{2}\right)R_{2}}{K_{2}}\right)}^{2}\omega_{2}},$$

where we have defined an effector with concentration $c_{2}$ that regulates the activity of repressor 2. Similarly, the dynamics for $R_{2}$ expression are described by an equation analogous to Eqn. 18 but swapping $R_{1}$ and $R_{2}$, given by

(19) $$\frac{\text{d}R_{2}}{\;\text{d}t}=-\gamma R_{2}\;+\;\frac{r}{1\;+\;2\frac{p_{\text{act}}\left(c_{1}\right)R_{1}}{K_{1}}\;+\;{\left(\frac{p_{\text{act}}\left(c_{1}\right)R_{1}}{K_{1}}\right)}^{2}\omega_{1}},$$

where we have also defined an effector with concentration $c_{1}$ that regulates the activity of repressor 1. The production term attributes a rate r to the state with no bound repressors as shown in Fig. 14. As in auto-activation, this analysis only accounts for the presence of RNA polymerase implicitly, which we discuss further in Appendix A 2. Note that having two effectors means that the control knob becomes a 2-dimensional vector $\left(c_{1},c_{2}\right)$. The bistable region becomes an area, and $\left(c_{1},c_{2}\right)$ can be tuned following arbitrary trajectories in the inducer concentration space. We will explore these threads in this section.

To continue the analysis, we can write dimensionless forms of these kinetic equations by transforming $\bar{t}=\gamma t$, ${\bar{R}}_{i}=R_{i}/K_{2}$, and $\bar{r}=r/\gamma K_{2}$, and obtain

(20) $$\frac{d{\bar{R}}_{1}}{d\bar{t}}=-{\bar{R}}_{1}\;+\;\frac{\bar{r}}{1\;+\;2p_{\text{act}}\left(c_{2}\right){\bar{R}}_{2}\;+\;\omega_{2}{\left[p_{\text{act}}\left(c_{2}\right){\bar{R}}_{2}\right]}^{2}},$$

(21) $$\frac{d{\bar{R}}_{2}}{d\bar{t}}=-{\bar{R}}_{2}\;+\;\frac{\bar{r}}{1\;+\;2p_{\text{act}}\left(c_{1}\right)\frac{{\bar{R}}_{1}}{\bar{K}}\;+\;\omega_{1}{\left[p_{\text{act}}\left(c_{1}\right)\frac{{\bar{R}}_{1}}{\bar{K}}\right]}^{2}}.$$

As a reminder, $\omega_{1}$ and $\omega_{2}$ are the cooperativity for ${\bar{R}}_{1}$ and ${\bar{R}}_{2}$, respectively, $c_{1}$ and $c_{2}$ are the inducer concentrations for each repressor, and $\bar{K}=K_{1}/K_{2}$ is the ratio of dissociation constants. Note that the equations above assume that each repressor responds to induction with the same inducer-protein binding chemistry, obeying the same activation probability function $p_{\text{act}}$, but responding to potentially different inducer concentrations $c_{1}$ and $c_{2}$. In the most general case, however, the two probability functions could differ.

This formulation now provides a two-dimensional knob for tuning the concentrations of the inducers corresponding to ${\bar{R}}_{1}$ and ${\bar{R}}_{2}$. The system may then be tuned to follow arbitrary trajectories in the two-dimensional parameter space spanned by $c_{1}$ and $c_{2}$. Frequently, this tuning generates non-trivial bifurcation curves.

**Figure 15:**
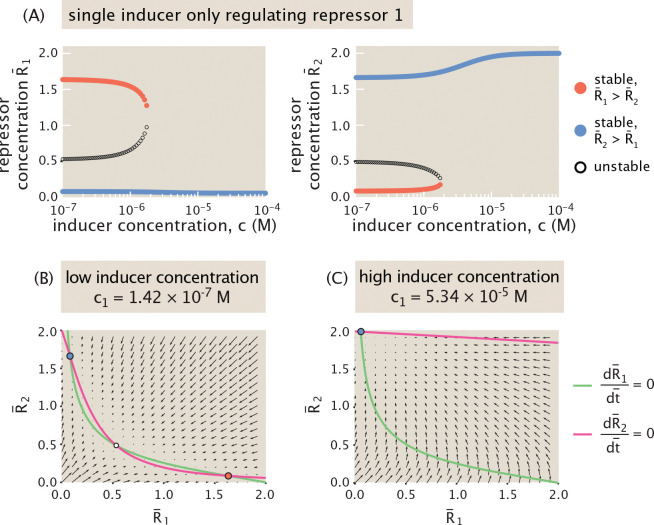
Bifurcation diagrams and phase portraits for mutual repression in the presence of a single inducer regulating the activity of repressor $R_{1}$ (with fixed parameters $\bar{K}=K_{1}/K_{2}=1$, $\bar{r}=2$ and $\omega_{1}=\omega_{2}=7.5$). (A) Bifurcation diagrams tracking steady-state ${\bar{R}}_{1}$ and ${\bar{R}}_{2}$ expression as inducer concentration increases. The red curves track expression in the steady state for which ${\bar{R}}_{1}>{\bar{R}}_{2}$, the blue curves track expression in the steady state for which ${\bar{R}}_{2}>{\bar{R}}_{1}$, and the unfilled nodes denote unstable saddle points. (B) Phase portrait at a low inducer concentration, demonstrating bistable dynamics between two possible stable states favoring either ${\bar{R}}_{1}$ (red) or ${\bar{R}}_{2}$ (blue). Steady states occur at the intersections of the nullclines as shown. (C) Phase portrait at a high inducer concentration, for which the system is monostable to favor ${\bar{R}}_{2}$.

To build intuition about this system, we first consider a scenario in which only one of the repressors may be induced. Fig. 15(A) plots the resulting bifurcation diagrams for ${\bar{R}}_{1}$ and ${\bar{R}}_{2}$ steady-state expression. At low inducer concentrations, the system is bistable and can either evolve to favor ${\bar{R}}_{1}$ (red) or ${\bar{R}}_{2}$ (blue) expression. Fig. 15(B) shows an example phase portrait within this low induction regime that depicts vector field flows through expression space toward these fixed points. At a sufficiently high inducer concentration, ${\bar{R}}_{1}$ expression can no longer be maintained, and the system transitions to a monostable regime in which only the state favoring ${\bar{R}}_{2}$ expression survives. A higher inducer concentration amplifies the expression of ${\bar{R}}_{2}$ up to its production limit. Fig. 15(C) depicts a phase portrait in this regime.

**Figure 16:**
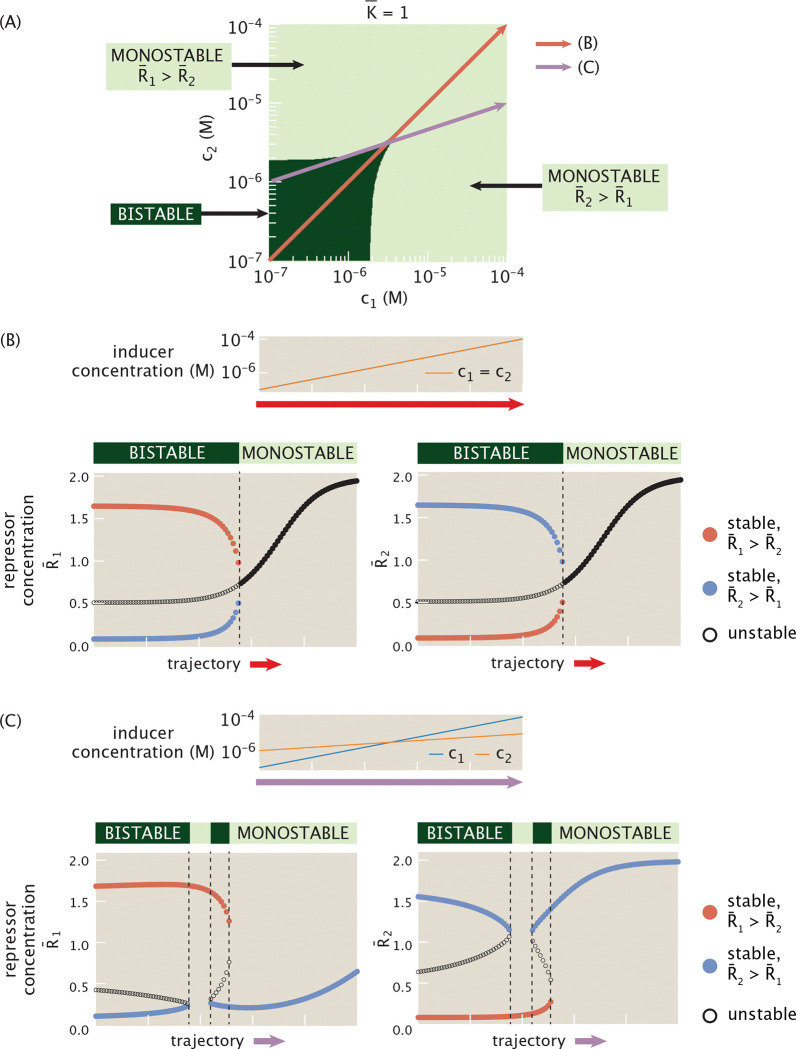
Phase diagram and bifurcation diagrams for mutual repression when each repressor’s activity is regulated by an inducer (with fixed parameters $\bar{K}=K_{1}/K_{2}=1$, $\bar{r}=2$ and $\omega_{1}=\omega_{2}=7.5$.) (A) Phase diagram in which the dark green region denotes the inducer concentration regime for which the system has bistable dynamics. Trajectories (*B*) and (*C*) follow the shifts in dynamics moving through phase space along different paths, and are shown in the corresponding panels. (B) Bifurcation diagrams for ${\bar{R}}_{1}$ and ${\bar{R}}_{2}$ steady-state expression as the inducer concentrations controlling each repressor increase at the same rate. (C) Bifurcation diagrams as inducer concentrations increase at different rates.

We now turn to a two-dimensional setting for allosteric regulation, with distinct inducers downregulating the activity of each repressor. Fig. 16(A) plots the phase diagram for the dynamics of mutual repression at different combinations of inducer concentrations. The dark green region denotes the region of parameter space with bistable dynamics, with monostable behavior elsewhere. Note the symmetry of this phase diagram with respect to the diagonal $c_{1}=c_{2}$. This is unsurprising given the condition $\bar{K}=1$ which amounts to saying that the two different repressors bind with the same dissociation constant. This condition will be discussed further in the following subsection.

We can now consider how dynamics evolve as inducer concentrations change at different rates. Fig. 16(A) considers different “protocols” for simultaneously varying the concentration of each inducer. For example, in Fig. 16(B), we examine a protocol in which the inducer concentration for each gene increases at the same constant rate (corresponding to the red trajectory shown in Fig. 16(A)). We then plot the corresponding bifurcation diagram. As inducer concentration increases, the scope of the bistable switch shrinks in expression space, with the stable states continuously approaching each other. At $c_{1}=c_{2}\approx 3.2\;\cdot \;10^{-6}\;M$, the system then undergoes a pitchfork bifurcation to monostable expression, stabilizing at increasingly high concentrations of both ${\bar{R}}_{1}$ and ${\bar{R}}_{2}$.

We could follow an alternative trajectory (denoted by the purple arrow in Fig. 16(A)) through parameter space such that the inducer concentrations evolve at different rates, in this case with $c_{1}$ increasing more rapidly than $c_{2}$. This purple trajectory then passes in and out of the green bistable region several times. Fig. 16(C) plots the corresponding bifurcation diagram tracking stable and unstable steady states as the inducer concentrations increase, and demonstrates the switches between bistability and monostability. Note that while the intermediate monostable regime favors ${\bar{R}}_{1}$ expression, the monostable regime at later times favors ${\bar{R}}_{2}$ instead, reflecting the swap in the dominant inducer concentration that occurs between these time periods. Thus, by modulating the induction dynamics of each repressor we can access a broad range of dynamical responses in repressor concentrations.

#### 1. Conditions for bistability

**Figure 17:**
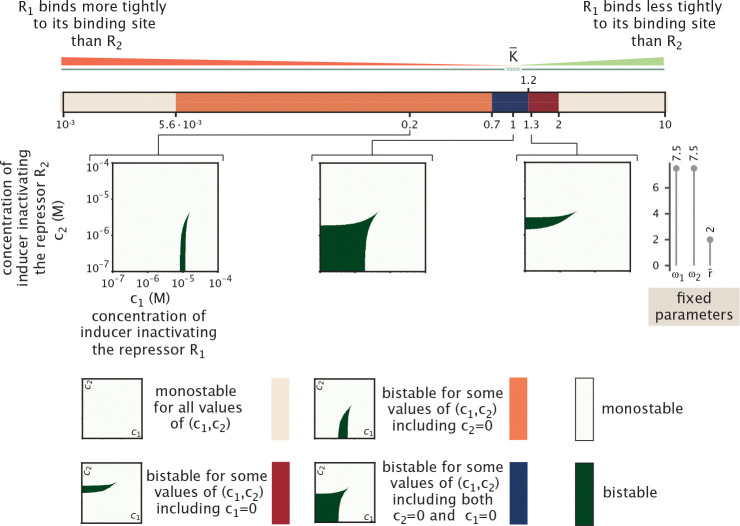
Bistability regimes in mutual repression as a function of the relative DNA-binding affinities of repressors ${\bar{R}}_{1}$ and ${\bar{R}}_{2}$. Colored regions denote distinct bistable phase space geometries, defined by whether bistability occurs for a finite window of $c_{1}$, a finite window of $c_{2}$, or the presence of both at sufficiently small concentrations. Otherwise, the system is never bistable for any concentration $\left(c_{1},c_{2}\right)$ in the interval [10^−7^M, 10^−4^M] considered. These geometries evolve as the binding affinity ratio $\bar{K}=K_{1}/K_{2}$ is varied, with $\omega_{1}=\omega_{2}=7.5$ and $\bar{r}=2$ held constant.

We now study how the size, shape, and symmetry of the bistable region observed in the phase diagram of Fig. 16(A) varies with system parameters. Specifically, in Fig. 17 we first identify three distinct geometries for the bistable region in the $\left(c_{1},c_{2}\right)$ plane, each reflecting different limiting behaviors of the inducers.

The first geometry (marked orange in the legend of Fig. 17) corresponds to a situation where bistability is present only when $c_{1}$ lies within a finite interval $\left[c_{1}^{\min},c_{1}^{\max}\right]$ for a given $c_{2}$. In this case, both bounds of the interval are strictly positive and finite, and $c_{2}$ must be smaller than a certain threshold. The limiting factor is therefore $c_{1}$, which must be finely tuned to enable bistability, while $c_{2}$ simply needs to remain below a critical level. Nevertheless, when decreasing $c_{2}$ the bistable interval in $c_{1}$ broadens, showing that lower $c_{2}$ expands the range of $c_{1}$ values supporting bistability.

A second geometry (marked red in the legend of Fig. 17) mirrors the first, but with the roles of $c_{1}$ and $c_{2}$ reversed. Here, bistability is present only when $c_{2}$ lies within a finite interval, while $c_{1}$ must remain below a threshold. In this case, $c_{2}$ becomes the more constrained parameter to tune. In contrast, a third geometry (marked blue in the legend of Fig. 17) arises when bistability is supported broadly for small enough values of both $c_{1}$ and $c_{2}$, with no lower bound required for either parameter. Although the bistable region remains upper-bounded, neither inducer is particularly limiting, with a broad range of concentrations allowing for bistability as long as neither becomes too large.

Fig. 17 illustrates how these phase space geometries for bistability depend on system parameters. We vary the relative DNA-binding affinity of the repressors by tuning $\bar{K}=K_{1}/K_{2}$, while keeping the basal production $\bar{r}$ fixed, and the cooperativities fixed and equal $\left(\omega_{1}=\omega_{2}\right)$. We observe that the most permissive bistable region—broad in both $c_{1}$ and $c_{2}$, for the range of concentration studied—occurs when $\bar{K}\approx 1$, corresponding to a symmetric system where both repressors bind with comparable affinity. As $\bar{K}$ decreases (i.e., $R_{1}$ binds more tightly than $R_{2}$), the phase space becomes increasingly constrained in $c_{1}$. If $c_{1}$ is too low, $R_{1}$ remains fully bound and strongly represses $R_{2}$, suppressing bistability. Conversely, if $c_{1}$ is too high, $R_{1}$ becomes fully unbound, leaving $R_{2}$ unrepressed and again eliminating bistability. Only an intermediate range of $c_{1}$ supports bistability in this regime, while $c_{2}$ simply needs to be small enough. A similar scenario occurs when $\bar{K}$ becomes large (i.e., $R_{2}$ binds more tightly than $R_{1}$), but with $c_{1}$ and $c_{2}$ effectively reversed. Eventually, $c_{2}$ is no longer sufficient to counteract the tight binding of $R_{2}$, and above a critical value of $\bar{K}$, bistability disappears entirely from the parameter space. Tuning the cooperativity parameter $\omega_{2}$ produces similar qualitative changes in the bistability phase space as varying the relative binding affinity $\bar{K}$.

In both cases, we observe the same sequence of transitions in the structure of the bistable region, as shown in Appendix J 3. High values of $\omega_{2}$ reflect strong cooperative binding of repressor $R_{2}$ to the DNA, meaning that binding becomes more favorable when two repressors are present. This effect mirrors what happens when increasing $\bar{K}$ : if $\bar{K}>1$, then $K_{1}>K_{2}$, implying that higher concentrations of $R_{1}$ are required for effective DNA binding compared to $R_{2}$. As a result, increasing $\bar{K}$ effectively enhances the influence of $R_{2}$, analogous to increasing $\omega_{2}$. Conversely, tuning $\omega_{1}$ affects the system similarly, but with the roles of $c_{1}$ and $c_{2}$ reversed. The effects of cooperativity are examined in greater detail in Appendix J 3.

From Fig. 17, we note that extreme (low or high) values of $\bar{K}$ tend to suppress bistability, as they strongly favor one repressor over the other across all concentrations. In contrast, high values of $\omega_{1}$ or $\omega_{2}$ amplify repression mostly when the corresponding repressor is present at high concentration. As a result, the system requires finely tuned inducer concentrations to counteract this cooperative imbalance and sustain bistability, effectively constraining the range of inducer concentrations for which the system can be bistable—as shown in Appendix J 3.

**Figure 18:**
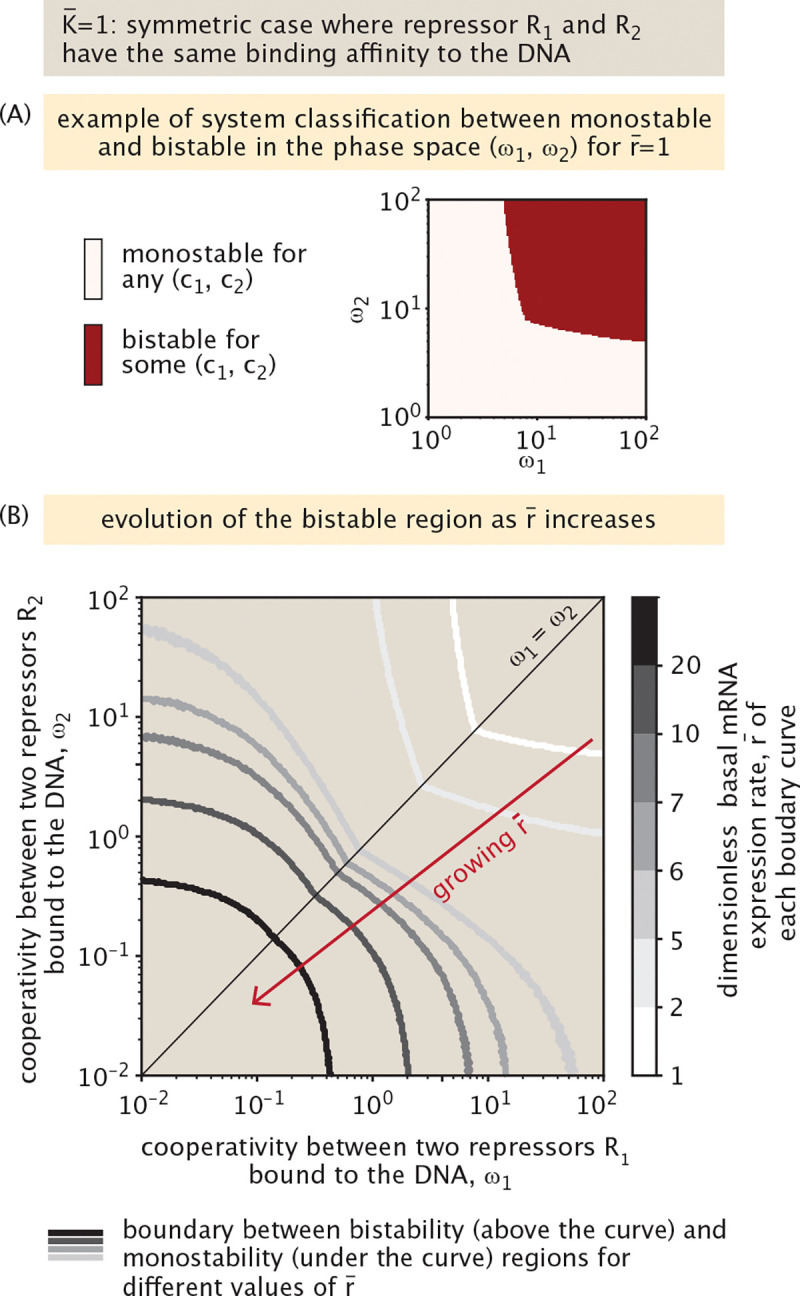
Bistability regions in mutual repression as a function of cooperativity and production rate. In both panels, the two repressors have equal DNA-binding affinities, with $\bar{K}=1$. We determine the boundary in $\left(\omega_{1},\omega_{2}\right)$ space that separates regions where bistability is possible for some $\left(c_{1},c_{2}\right)$ from those where it is not possible. (A) Example for $\bar{r}=1$. (B) Boundary curves in the $\left(\omega_{1},\omega_{2}\right)$ plane showing how the region of possible bistability expands as $\bar{r}$ increases. Each curve corresponds to fixed $\bar{r}$ values and separates parameter combinations that are monostable for all $\left(c_{1},c_{2}\right)$ (below the curve) from combinations that are bistable for some $\left(c_{1},c_{2}\right)$ (above the curve). Colors indicate the value of r used to compute each boundary curve.

We next explore how the interplay between cooperativity and production rate controls the presence and extent of bistability in mutual repression systems, focusing on the symmetric case $\bar{K}=1$ shown in Fig. 18. We note that, in contrast to parameters like $\omega_{1}$, $\omega_{2}$, or K, tuning the rate parameter $\bar{r}$ does not break the symmetry between the two genes, as it controls the production rate of both repressors equally.

In Fig. 18(A), we classify parameter combinations in the $\left(\omega_{1},\omega_{2}\right)$ plane for $\bar{r}=1$ according to whether the system exhibits bistability for any inducer concentrations $\left(c_{1},c_{2}\right)$. Fig. 18(B) shows how the boundary between monostable and bistable regimes shifts with $\bar{r}$, separating regions where bistability is either inaccessible or achievable for at least some inducer pairs. We observe that the production rate, when coupled to cooperativity, plays a critical role in enabling bistability—much like in the auto-activation system, where the product $\omega {\bar{r}}_{2}$ must exceed a threshold to generate bistability. We quantify this cooperative relationship between $\omega_{i}$ and $\bar{r}$ further in Appendix J 1 by deriving a necessary condition for bistability where

(22) $$\bar{r}>\frac{1}{p_{\text{act}}^{\max}\;-\;p_{\text{act}}^{\min}\;+\;\min\left(\omega_{2},\;\omega_{1}\right)p_{\text{act}}^{\max}/2},$$

with $p_{\text{act}}^{\max}$ and $p_{\text{act}}^{\min}$ defined in Eqns. 6 and 7.

Increasing $\bar{r}$ systematically expands the range of cooperativity values that can support bistability in the range of $\bar{r}$ swept in Fig. 18. We observe from the figure that at low production rates, bistability only arises when both repressors exhibit stabilizing cooperative binding to DNA $\left(\omega_{i}>1\right)$. As $\bar{r}$ increases, this constraint relaxes: bistability becomes possible even without cooperativity $\left(\omega_{1}=\omega_{2}=1\right)$, and for sufficiently high $\bar{r}$, bistability can occur even in the presence of destabilizing interactions between the two repressors $\left(\omega_{i}<1\right)$. Intermediate production rates typically require at least one positively cooperative repressor.

In auto-activation systems, the effective Hill coefficient can vary above or below one, but bistability only occurs when it exceeds one. In contrast, for mutual repression, the effective Hill coefficients for $R_{1}$ and $R_{2}$ vary with parameters but remain strictly greater than one when $\omega_{1}$, $\omega_{2}>0$ (Appendix J 1), making the Hill coefficient less informative about the existence of bistability. Even in models using empirical Hill functions to describe the production terms, Hill coefficients above one are not always sufficient for bistability, and computational studies show that extended network interactions can yield bistability even with coefficients below one [93].

#### 2. Timescale for stabilization

**Figure 19:**
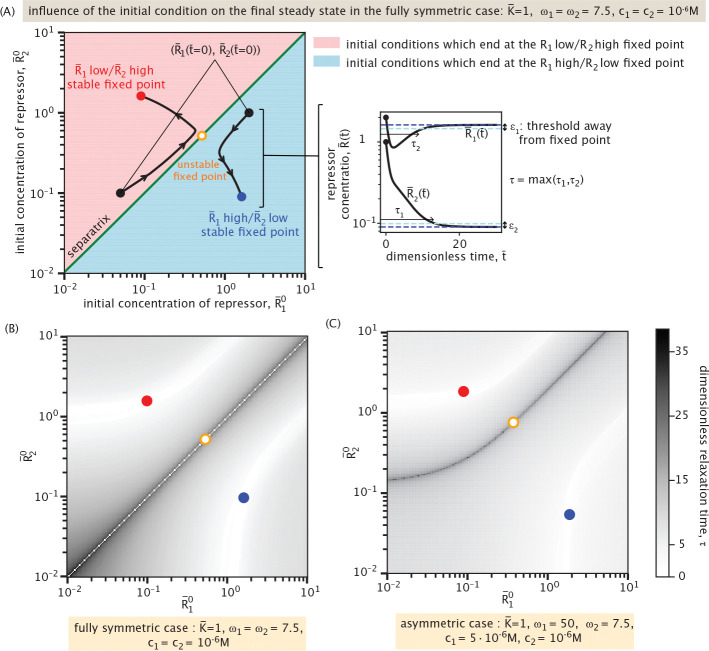
Dynamics and relaxation time in a mutual repression system. (A) Influence of the initial condition on the final steady state for a symmetric case $\left(\bar{K}=1,\omega_{1}=\omega_{2}=7.5,c_{1}=c_{2}=10^{-6}\;\text{M}\right)$. Two stable steady states and one unstable steady state are shown. The colored regions correspond to sets of initial conditions that converge to each respective stable steady state. The green curve separating these regions is the separatrix. Dimensionless relaxation times $\tau_{1}$ and $\tau_{2}$ are defined as the time required for the system to approach within thresholds $\epsilon_{1}$ and $\epsilon_{2}$ of the stable fixed points, where these thresholds correspond to 95% of the respective steady-state values. The global relaxation time of the system, denoted τ, is then defined as the maximum of the two: $\tau =\max\left(\tau_{1},\tau_{2}\right)$. (B-C) Dimensionless relaxation time as a function of initial concentrations of repressors $R_{1}$ and $R_{2}$. Panel (B) corresponds to the symmetric case, whereas panel (C) plots an asymmetric case $\left(K=1,\omega_{1}=50,\omega_{2}=7.5,c_{1}=5\cdot 10^{-6}\;\text{M},c_{2}=10^{-6}\;\text{M}\right)$.

Beyond identifying the final steady state of the mutual repression system, it is important to characterize the time required for the system to reach steady state starting from different initial conditions. We define the relaxation time τ as the maximum of the times $\tau_{1}$ and $\tau_{2}$ taken by the two trajectories, ${\bar{R}}_{1}(\bar{t})$ and ${\bar{R}}_{2}(\bar{t})$, to reach 95% of their respective steady states. Fig. 19(A) illustrates the influence of the initial condition on the final steady state in a symmetric system, where $\bar{K}=1$, $\omega_{1}=\omega_{2}=7.5$, and $c_{1}=c_{2}=10^{-6}\;M$. The figure reveals that the phase space is divided into two basins of attraction, each leading to one of the two stable steady states. The separatrix, plotted in green, denotes the boundary between these basins, and is derived analytically in Appendix K.

Fig. 19(B) and (C) quantify the relaxation time τ across the phase space $\left({\bar{R}}_{1}^{0},{\bar{R}}_{2}^{0}\right)$ for both symmetric and asymmetric parameter regimes. In both cases, the relaxation time is shorter when the initial condition lies far from the separatrix and near the final stable steady state. In contrast, initial conditions close to the separatrix result in significantly longer relaxation times, as the system evolves slowly near the unstable fixed point before diverging toward a stable state. Exactly on the separatrix, the relaxation to steady state is more difficult to quantify because the system may remain indefinitely near an unstable manifold without converging to either stable fixed point. In practice, however, even minimal noise in a real system will eventually drive the system away from this unstable region toward a stable state. For this reason, we disregard the white line—signifying artificially short relaxation times—observed exactly on the separatrix in Fig. 19(B). This feature is absent in Fig. 19(C), as the separatrix has a more complex shape and the numerical sweep over initial conditions does not sample it precisely.

In the asymmetric case (Fig. 19(C)), with $\omega_{1}=50$, $\omega_{2}=7.5$, and distinct inducer concentrations $\left(c_{1}=5\cdot 10^{-6}\;\text{M},c_{2}=10^{-6}\;\text{M}\right)$, the phase space becomes skewed. The separatrix, corresponding to the ridge of maximal relaxation time in the grayscale colormap, delineates the boundary between the two basins of attraction. We do not overlay it explicitly, as doing so would interfere with the visualization of the relaxation times, which are particularly sensitive near this boundary. Compared to Fig. 19(B), where the separatrix coincides with the diagonal ${\bar{R}}_{1}^{0}={\bar{R}}_{2}^{0}$ due to the symmetry of the system, we observe that the separatrix is now deformed, and the relative sizes of the basins of attraction have shifted. Despite these geometric changes, the maximal relaxation times across both regimes remain comparable. This indicates that, although asymmetry reshapes the phase space and can affect which attractor is reached, it does not substantially alter the overall timescale required for the system to stabilize. The main contribution to long relaxation times remains the initial conditions–and how close they are to the separatrix–regardless of symmetry.

This whole section has had as its primary ambition to carefully consider the famed mutual repression genetic switch from the new perspective in which the two repressors are controlled separately by different effector molecules. We have seen that the steady-states and the dynamics in this case are extremely rich, making it clear that there is much freedom in the biological context to exploit different kinds of behavior.

## iv. KINETICS AND TIME DELAYS IN FEED-FORWARD LOOPS

**Figure 20:**
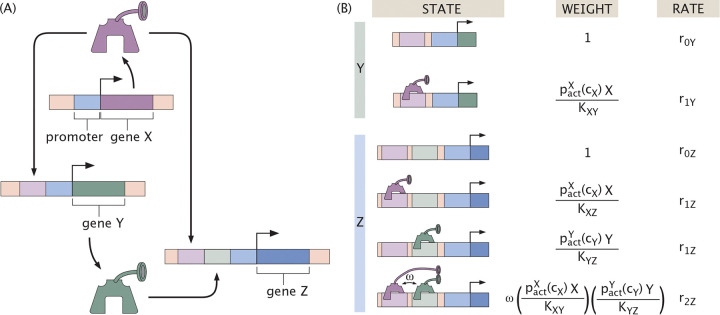
The coherent feed-forward loop. (A) Schematic representation of the coherent feed-forward loop. Expression of output protein Z is controlled by expression of protein X, either by direct activation or indirectly, first activating expression of Y which in turn activates Z. The regulatory circuit is coherent because both pathways have the same activating effect on Z. (B) Thermodynamic states, weights, and rates for expression of activator Y and output protein Z. Note that the model assumes both X and Y can bind together to activate expression at their respective binding sites, interacting with cooperativity ω.

In this section, we consider gene circuits whose functionality appears in their dynamics rather than in their steady state responses. Specifically, we focus on a three-gene circuit called the feed-forward loop shown schematically in Fig. 20(A) [71]. Here, we denote the input genes as X and Y, and the output gene as Z. In a feed-forward loop, X regulates Y, and X and Y together regulate Z. X thus controls expression of output Z through both direct and indirect regulatory paths. This network typically features a sign-sensitive delayed or accelerated response (depending on architecture) to a step-wise change in the effector concentration for protein X [71]. That is, while the qualitative nature of the response (delay or acceleration) remains fixed, its magnitude depends on the sign (an increase or a decrease) of the input change. This delayed or accelerated response has been hypothesized to have important biological consequences, particularly in information-processing systems that filter noisy inputs [71]. Beyond their role in shaping temporal responses, coherent feed-forward loops have been shown to attenuate input noise, thereby enhancing the reliability of gene expression [94–96].

Interestingly, there are various architectures of feed-forward loop depending on whether X and Y work together or at cross purposes. We will largely focus on the particular architecture where X activates Y and Z, and Y also activates Z, which is the so-called type I coherent feed-forward loop, with the word “coherent” attached to this architecture since X and Y alter the expression of Z in a coherent manner. We consider this particular motif primarily because at the time of the most recent census of regulatory architectures in *E. coli*, this version of the feed-forward loop appeared the most frequently [97, 98]. The logic of our analysis can be applied to any of the other feed-forward architectures as well.

Previous literature explores feed-forward loops from a dynamical systems perspective using Hill functions to model transcription factor-DNA interactions and considering the effector concentration for X as a Boolean variable that is either fully on or fully off [71]. We build upon that earlier analysis also by making a systematic search for network parameters that gives rise to various functions. Our goal is to expand the theoretical understanding of the feed-forward loop architecture by incorporating the thermodynamic model to describe transcription factor binding to the DNA and to explicitly include effector function. Specifically, we explore what gives rise to the dynamical features of the feed-forward loop, the robustness of such features, and the effect of continuously tuning the effector concentration.

As usual when writing the dynamical equations, we begin with the states, weights and rates for the regulatory architecture of interest. Fig. 20(B) provides the states, weights and rates for the coherent feed-forward loop architecture, where we assume one binding site per transcription factor. In light of the states and weights, we can write the time-evolution equations for the coherent feed-forward loop as

(23) $$\frac{dY}{dt}=-\gamma Y\;+\;\frac{r_{0Y}\;+\;r_{1Y}p_{\text{act}}^{X}\left(c_{X}\right)\frac{X}{K_{XY}}}{1\;+\;p_{\text{act}}^{X}\left(c_{X}\right)\frac{X}{K_{XY}}}$$

for the regulation of Y by X and

(24) $$\frac{dZ}{dt}=-\gamma Z\;+\;\frac{r_{0Z}\;+\;r_{1Z}\left(p_{\text{act}}^{X}\left(c_{X}\right)\frac{X}{K_{XZ}}\;+\;p_{\text{act}}^{Y}\left(c_{Y}\right)\frac{Y}{K_{YZ}}\right)\;+\;r_{2Z}\omega p_{\text{act}}^{X}\left(c_{X}\right)\frac{X}{K_{XZ}}p_{\text{act}}^{Y}\left(c_{Y}\right)\frac{Y}{K_{YZ}}}{1\;+\;p_{\text{act}}^{X}\left(c_{X}\right)\frac{X}{K_{XZ}}\;+\;p_{\text{act}}^{Y}\left(c_{Y}\right)\frac{Y}{K_{YZ}}\;+\;\omega p_{\text{act}}^{X}\left(c_{X}\right)\frac{X}{K_{XZ}}p_{\text{act}}^{Y}\left(c_{Y}\right)\frac{Y}{K_{YZ}}}$$

for the regulation of Z by both X and Y. We assume here that the proteins Y and Z have the same degradation rate γ. The production rates and dissociation constants are assumed to be different in general for each thermodynamic state. Specifically, $r_{iG}$ denotes the production rate of gene G when i number of transcription factor are bound. Further, $K_{G_{1}G_{2}}$ denotes the dissociation constant of transcription factor $G_{1}$ binding to gene $G_{2}$. ω is the cooperativity, which takes into account the extra interaction energy between X and Y when bound to the DNA. Finally, the probabilities $p_{\text{act}}^{X}\left(c_{X}\right)$ and $p_{\text{act}}^{Y}\left(c_{Y}\right)$ scale the activity of transcription factors X and Y. Note also that we consider different effectors $c_{X}$ and $c_{Y}$ for the two genes that can be varied independently. These functions may be distinct in principle, and the analytic discussions here make no assumption regarding their nature. For numerical results, however, we assume these probability functions, and thus effector activity functions, to be the same regardless of target transcription factor for simplicity.

We non-dimensionalize the equations by using 1/γ as our unit of time and $K_{XY}$ as our measure of concentration. In light of these conventions, we arrive at

(25) $$\frac{d\bar{Y}}{d\bar{t}}=-\bar{Y}\;+\;\frac{{\bar{r}}_{0Y}\;+\;{\bar{r}}_{1Y}p_{\text{act}}^{X}\left(c_{X}\right)\bar{X}}{1\;+\;p_{\text{act}}^{X}\left(c_{X}\right)\bar{X}},$$

and

(26) $$\frac{d\bar{Z}}{d\bar{t}}=-\bar{Z}\;+\;\frac{{\bar{r}}_{0Z}\;+\;{\bar{r}}_{1Z}(𝒳\;+\;𝒴)\;+\;\omega {\bar{r}}_{2Z}𝒳𝒴}{1\;+\;𝒳\;+\;𝒴\;+\;\omega 𝒳𝒴}.$$

To make subsequent analysis less cumbersome, we further introduce $𝒳=p_{\text{act}}^{X}\left(c_{X}\right)\bar{X}/{\bar{K}}_{XZ}$ and $𝒴=p_{\text{act}}^{Y}\left(c_{Y}\right)\bar{Y}/{\bar{K}}_{YZ}$ as simpler notation for the effective regulatory contributions of $\bar{X}$ and $\bar{Y}$ to $\bar{Z}$ expression. The bar indicates quantities where time is measured in units of 1/γ, and where concentration and dissociation constants are measured in units of $K_{XY}$. Specifically, we define dimensionless dissociation constants ${\bar{K}}_{XZ}=K_{XZ}/K_{XY}$ and ${\bar{K}}_{YZ}=K_{YZ}/K_{XY}$. The rates are then in units of $\gamma K_{XY}$. Note that this model fixes the concentration of $\bar{X}$ for simplicity, such that its activity regulating $\bar{Y}$ and $\bar{Z}$ depends entirely on effector concentration $c_{X}$.

### A. Characterizing delay responses in coherent feed-forward loops

Previous work has shown that coherent feed-forward loops can delay a system’s response to an input signal [71]. Here, we demonstrate from our thermodynamic modeling perspective the analytic origins of this delay. In particular, we rigorously define how the introduction of an indirect but coherent path for regulation of output $\bar{Z}$ affects its response.

Suppose that we keep the effector concentration $c_{Y}$ fixed, and that at time $\bar{t}=0$ the effector concentration $c_{X}$ changes sharply from an initial concentration $c_{X}^{i}$ to a final concentration $c_{X}^{f}$ such that input $\bar{X}$ activity either increases (an “ON” step) or decreases (an “OFF” step). This then means that

(27) $$𝒳(\bar{t})=\left\{\begin{array}{ccc} {𝒳}_{i}=\frac{p_{\text{act}}^{X}(c_{X}^{i})\bar{X}}{{\bar{K}}_{XZ}} & \text{if} & \bar{t}\leq 0,\\ {𝒳}_{f}=\frac{p_{\text{act}}^{X}(c_{X}^{f})\bar{X}}{{\bar{K}}_{XZ}} & \text{if} & \bar{t}>0. \end{array}\right.$$

In the coherent feed-forward loop, since all the regulatory relations are activation, both $\bar{Y}$ and $\bar{Z}$ increase in response to an ON step, and decrease in response to an OFF step.

To understand intuitively how exactly the feed-forward loop regulatory structure responds to such a switch in input signal, let us first consider a simpler scheme in which $\bar{X}$ no longer regulates $\bar{Y}$, leaving $\bar{X}$ and $\bar{Y}$ to regulate $\bar{Z}$ independently with $\bar{Y}(\bar{t})=\bar{Y}$ fixed at a value. Since we are keeping effector concentration $c_{Y}$ fixed constant, this then also means that 𝒴 is constant. We refer to this setting as “simple regulation.”

Before the switch in $c_{X}$, the simple regulation system is at steady state, with initial output expression ${\bar{Z}}_{s}^{i}$ where subscript s denotes simple regulation. After the switch, the system relaxes to a new steady state with final expression ${\bar{Z}}_{s}^{f}$. For $\bar{t}>0$, the production term in Eqn. 26 is simply a constant, yielding the following differential equation for $\bar{Z}$.


(28) $$\begin{array}{l} \frac{d\bar{Z}}{d\bar{t}}=-\bar{Z}\;+\;\frac{{\bar{r}}_{0Z}\;+\;{\bar{r}}_{1Z}\left({𝒳}_{f}\;+\;𝒴\right)\;+\;\omega {\bar{r}}_{2Z}{𝒳}_{f}𝒴}{1\;+\;{𝒳}_{f}\;+\;𝒴\;+\;\omega {𝒳}_{f}𝒴}\\ \;\equiv -\bar{Z}\;+\;{\bar{Z}}_{s}^{f}. \end{array}$$

Integrating Eqn. 28, we thus determine that under simple regulation, output $\bar{Z}$ evolves after the switch in input $\bar{X}$ signal by a standard exponential behavior defined as

(29) $${\bar{Z}}_{s}(\bar{t})={\bar{Z}}_{s}^{i}e^{-\bar{t}}\;+\;{\bar{Z}}_{s}^{f}(1-e^{-\bar{t}}).$$

By contrast, in the coherent feed-forward loop, the concentration of $\bar{Y}$ directly depends on $\bar{X}$ as seen in Eqn. 25. As a result, additional time is needed for $\bar{Y}$ to evolve from its initial steady-state value ${\bar{Y}}_{i}$ to a new value ${\bar{Y}}_{f}$ following a change in $\bar{X}$. Expressed mathematically, since $\bar{Y}$ itself is a function of time, the solution of $\bar{Z}$ to Eqn. 26 is not strictly an exponential relaxation to steady state. More formally, output expression for the coherent feed-forward loop evolves by a function of the form

(30) $$\bar{Z}(\bar{t})={\bar{Z}}_{i}e^{-t}\;+\;{\bar{Z}}_{f}\left(1-e^{-t}\right)\;+\;\text{Θ}(\bar{t}),$$

where ${\bar{Z}}_{i}$ is the initial steady state in the feed-forward setting before the change in $c_{X}$, and ${\bar{Z}}_{f}$ is the final steady state after the change that $\bar{Z}(\bar{t})$ relaxes to eventually. We derive Eqn. 30 explicitly from Eqns. 25 and 26 in Appendix L. Note that the sum of the first two terms in Eqn. 30 describes behavior of the same form as simple regulation in Eqn. 29. Therefore, by rescaling Eqn. 29 we can treat the exponential portion of Eqn. 30 as an equivalent ${\bar{Z}}_{\text{simple}}$ with the same relaxation dynamics as observed for simple regulation. We can also then express output response for the feed-forward loop as

(31) $$\bar{Z}(\bar{t})={\bar{Z}}_{\text{simple}}\;(\bar{t})\;+\;\text{Θ}(\bar{t}).$$

We thus observe that the feed-forward loop’s output response differs from behavior in simple regulation by a function $\text{Θ}(\bar{t})$. Analytically solving Eqns. 25 and 26 results in

(32) $$\text{Θ}(\bar{t})=-\frac{\text{Φ}\Delta 𝒴}{S^{2}}e^{\;-\;\bar{t}}\log\left(\frac{Se^{\bar{t}}\;-\;\text{Δ}𝒴\left(1\;+\;\omega {𝒳}_{f}\right)}{S\;-\;\text{Δ}𝒴\left(1\;+\;\omega {𝒳}_{f}\right)}\right).$$

Here, $\text{Θ}(\bar{t})$ depends on the three quantities Δ𝒴, Φ, and S. First, the quantity

(33) $$\Delta 𝒴={𝒴}_{f}-{𝒴}_{i}=\frac{p_{\text{act}}^{Y}\left(c_{Y}\right)}{{\bar{K}}_{YZ}}\left({\bar{Y}}_{f}-{\bar{Y}}_{i}\right)$$

denotes the total change in quantity 𝒴 in response to the change in input effector concentration $c_{X}$. Δ𝒴 thus contains implicit information about regulation of $\bar{Y}$ by $\bar{X}$ from its evolution as defined in Eqn. 25. $\Theta (\bar{t})$ also depends on the coefficient

(34) $$\begin{array}{l} \Phi =\omega {\left({𝒳}_{f}\right)}^{2}\left({\bar{r}}_{2Z}-{\bar{r}}_{1Z}\right)\;+\;\omega {𝒳}_{f}\left({\bar{r}}_{2Z}-{\bar{r}}_{0Z}\right)\\ \;\;+\;\left({\bar{r}}_{1Z}-{\bar{r}}_{0Z}\right), \end{array}$$

which encodes how the rates and cooperativity regulate output $\bar{Z}$ expression as a function of input signal ${𝒳}_{f}$. Interestingly, we note that all types of feed-forward loops have the same solution as Eqn. 32, except with a potentially different Φ. Appendix L 4 discusses this in more detail. Finally, the quantity

(35) $$S=1\;+\;{𝒳}_{f}\;+\;{𝒴}_{f}\;+\;\omega {𝒳}_{f}{𝒴}_{f}$$

is the sum of the dimensionless weights for all possible regulatory states with zero, one, or two transcription factors bound, at final concentrations of active $\bar{X}$ and $\bar{Y}$.

**Figure 21:**
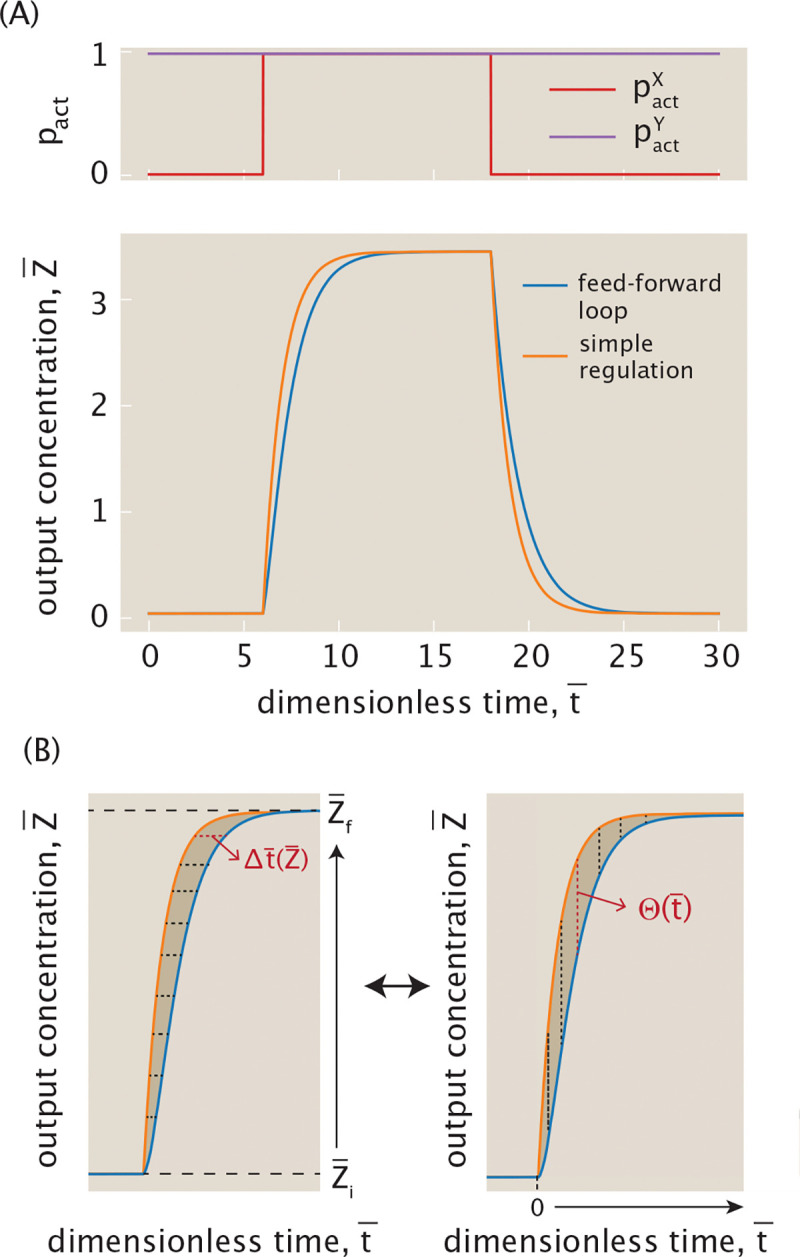
Delay in coherent feed-forward loop response compared to simple regulation. (A) The input signal is applied by tuning $c_{X}$ as a step function: from high (10^−4^ M) to low (10^−7^ M) at $\bar{t}=6$, and back to high at $\bar{t}=18$. The effector concentration $c_{Y}$ is held constant at 10^−7^ M. These inputs determine the activation probabilities $p_{\text{act}}^{X}$ and $p_{\text{act}}^{Y}$, shown in red and purple, respectively. The second panel plots the time evolution of the dimensionless output concentration $\bar{Z}(\bar{t})$ under feed-forward and simple regulation schemes, with ${\bar{r}}_{0Y}={\bar{r}}_{0Z}=0$, ${\bar{r}}_{1Y}={\bar{r}}_{1Z}=2$, ${\bar{r}}_{2Z}=10$, ω=1, and ${\bar{K}}_{XZ}={\bar{K}}_{YZ}=1$. (B) Schematic demonstrating the two ways to equivalently quantify the delayed response of the feed-forward loop compared to simple regulation, captured by the shaded area between the two curves. One can either integrate over individual time delay measurements $\text{Δ}\bar{t}(\bar{Z})$ as a function of $\bar{Z}$, or equivalently integrate the difference in responses $\text{Θ}(\bar{t})$ as a function of $\bar{t}$.

Fig. 21(A) highlights the coherent feed-forward loop’s delayed response to changes in input signal for a specific set of parameters. In our simulations, we set the rates ${\bar{r}}_{0G}$ to zero, indicating that this system requires activator(s) to be bound to express output $\bar{Z}$. In the top plot of Fig. 21(A), the effector concentration $c_{Y}$ is fixed such that $p_{\text{act}}^{Y}\left(c_{Y}\right)$ is always at high activity, and the effector concentration $c_{X}$ jumps such that $p_{\text{act}}^{X}\left(c_{X}\right)$ reaches high activity as a step function (ON step), and then back to low activity (OFF step). The bottom plot of Fig. 21(A) shows how the output $\bar{Z}$ evolves over time in response to a step function input $\bar{X}$ activity, evolving and stabilizing to a high $\bar{Z}$ value before the OFF step in $p_{\text{act}}^{X}\left(c_{X}\right)$ causes the output concentration to decay back down to steady state value zero. We observe that, compared to simple regulation, the change in output concentration $\bar{Z}$ is slower when responding to both an increase and a decrease in input activity, matching our expectation.

Fig. 21(B) visualizes how to quantify this delay in the time it takes the feed-forward system to reach a given output concentration as it responds to an input pulse. The diagram on the left illustrates the response to the ON step, in which the output starts to increase from ${\bar{Z}}_{i}$ to ${\bar{Z}}_{f}$ at (dimensionless) time $\bar{t}=0$. If we choose a value of $\bar{Z}$ in this time frame, we observe that it takes longer to reach this value on its way to steady state ${\bar{Z}}_{f}$ in the feed-forward loop setting than in simple regulation. We highlight one such horizontal distance between the two curves as the time delay $\Delta \bar{t}(\bar{Z})$. Explicitly, we define $\Delta \bar{t}(\bar{Z})$ to be the difference between the time it takes for simple regulation to reach a given $\bar{Z}$ and that for feed-forward loop. $\Delta \bar{t}(\bar{Z})<0$ signifies a delay and $\Delta \bar{t}(\bar{Z})>0$ signifies acceleration. We can then compute the average time difference observed between the two curves by integrating $\Delta \bar{t}(\bar{Z})$ over the range of output $\bar{Z}$, and normalizing by this range. Therefore, for a given step function change in input $\bar{X}$ activity, the average delay during the system’s evolution toward its new final steady state is

(36) $$\langle \Delta \bar{t}\rangle =\frac{1}{\left|{\bar{Z}}_{f}\;-\;{\bar{Z}}_{i}\right|}\int_{{\bar{Z}}_{i}}^{{\bar{Z}}_{f}}\Delta \bar{t}(\bar{Z})d\bar{Z}.$$

Note, however, that it is not straightforward to derive an expression for $\Delta \bar{t}(\bar{Z})$. Instead, since this integral geometrically captures the area between the two curves, we can equivalently evaluate this area as shown in the second diagram of Fig. 21(B) by integrating vertical slices through this shaded region. At a given time $\bar{t}$, the vertical dotted line corresponds to the difference in output response between the two curves, defined by the function $\Theta (\bar{t})$ previously derived in Eqn. 32.

From this description, we can then also derive the average time delay from the offset $\Theta (\bar{t})$, and thus arrive at the equivalent definition

(37) $$\langle \Delta \bar{t}\rangle =\frac{1}{{\bar{Z}}_{f}\;-\;{\bar{Z}}_{i}}\int_{0}^{\infty }\Theta (\bar{t})d\bar{t}.$$

Notice that the absolute value on ${\bar{Z}}_{f}-{\bar{Z}}_{i}$ is dropped to match the sign of $\langle \Delta \bar{t}\rangle$ in Eqn. 36. Substituting $\Theta (\bar{t})$ from Eqn. 32 and evaluating the integral, the average time delay becomes

(38) $$\langle \Delta \bar{t}\rangle =\frac{\Phi {\left({\bar{Z}}_{f}\;-\;{\bar{Z}}_{i}\right)}^{-1}}{S\left(1\;+\;\omega {𝒳}_{f}\right)}\log\left(\frac{1\;+\;{𝒳}_{f}\;+\;{𝒴}_{i}\;+\;\omega {𝒳}_{f}{𝒴}_{i}}{S}\right).$$

This result highlights that $\langle \Delta \bar{t}\rangle$ depends on both the initial state and the final state of 𝒳 and 𝒴. In fact, it is this dual dependence that causes the delays in response to ON and OFF steps to differ in Fig. 21(A). Switching from ON to OFF and vice versa simply swaps the initial and final expression states of 𝒳 and 𝒴. Applying these transformations ${𝒳}_{i}↔{𝒳}_{f}$ and ${𝒴}_{i}↔{𝒴}_{f}$ in Eqn. 38 shows that the magnitude of the average delay $\langle \Delta \bar{t}\rangle$ is in general not conserved under the exchange of initial and final states. It is therefore this asymmetric dependence on initial and final states that directly leads to differences in output responses to ON and OFF steps in the coherent feed-forward loop.

From Eqn. 38, we can analytically deduce whether a feed-forward loop delays or accelerates output response from the sign of $\langle \Delta \bar{t}\rangle$. In Appendix L 2, we prove that in general $\langle \Delta \bar{t}\rangle \leq 0$ for the coherent feed-forward loop, leading to delay for both the ON and OFF steps as seen in the example of Fig. 21(A).

### B. Robustness of delayed response for different coherent logic gates

While Fig. 21(A) demonstrates delays in both the ON and OFF step of the feed-forward loop for an arbitrary choice of the dimensionless rates, dissociation constants, and cooperativity, distinct choices for this set of parameters lead to different magnitudes of delay. Returning to Fig. 20, the thermodynamic states and weights listed here are defined generally such that all of the states can contribute to transcription factor expression, and this is reflected in the example feed-forward loop shown in Fig. 21(A). However, certain alternative choices for parameters can carry physical significance because they restrict the regulatory states allowing expression to only a subset of those depicted in Fig. 20. How would the feed-forward loop’s behavior differ, for example, if expression could only be enhanced when both activators are bound? We use the framework of *logic gates* to define such unique categories for regulatory conditions.

Specifically, we highlight three commonly-encountered logic gates—the AND, XOR, and OR gates. We will assume here that all gates can express output $\bar{Z}$ at a basal level, as defined by rate ${\bar{r}}_{0Z}$ for the state. Each logic gate is then characterized by a different set of parameters for the states in which one or both activators can be bound, and these parameters determine whether a given state’s expression is enhanced or remains unaffected at the basal level.

In the AND gate, Z expression is enhanced only when both X and Y are bound. From the description in Fig. 20, this corresponds to systems in which cooperativity ω is nonzero and ${\bar{r}}_{2Z}>{\bar{r}}_{1Z}={\bar{r}}_{0Z}$, such that X and Y have no activating effect on basal expression unless simultaneously bound. In the XOR gate, X and Y cannot be bound at the same time (ω=0), and single-activator bound states enhance Z expression $\left({\bar{r}}_{1Z}>{\bar{r}}_{0Z}\right)$. Finally, the OR gate allows enhanced expression when either X, Y, or both are bound, and broadly applies to systems for which ${\bar{r}}_{2Z}\geq {\bar{r}}_{1Z}>{\bar{r}}_{0Z}$. Note that the expression rates when one or both transcription factors bind can differ.

In previous work, XOR and AND gates have been reported to exhibit sign-sensitive delays in response to a signal change in $c_{X}$ : the XOR gate feed-forward loop delays the OFF step but not the ON step, while the AND gate delays the ON step but not the OFF step [71]. While these descriptions hold for certain parameter choices, it remains unclear how robust these patterns are across parameter space as the conditions governing regulatory interactions change. We will now examine the conditions under which the feed-forward loop response delays both ON and OFF steps, delays only one, or delays neither as we compare the different types of logic gates.

To evaluate the robustness of time delays across different types of logic gates, we sweep across parameter space and find regions with high average delay, $\langle \Delta \bar{t}\rangle$. For now, we choose to fix rate and cooperativity parameters and sweep across the two-dimensional space $\left({\bar{K}}_{XZ},{\bar{K}}_{YZ}\right)$. The motivation is to find the suitable dissociation constants given a logic gate, in the case in which the production rates and cooperativity must satisfy certain requirements. For example, in the XOR gate, ω=0 is fixed and is not a tunable parameter.

**Figure 22:**
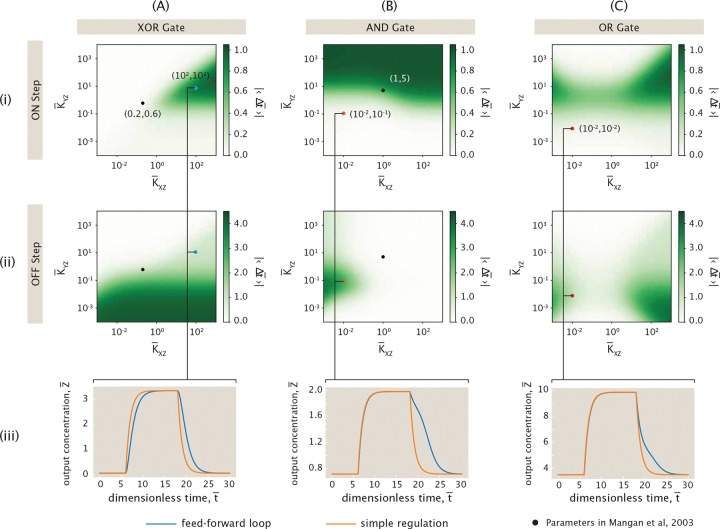
Magnitude of average time delay observed across parameter space in the ON and OFF steps of different coherent feed-forward logic gates. Each colorplot shows $\text{Δ}\bar{t}$ as a function of ${\bar{K}}_{XZ}$ and ${\bar{K}}_{YZ}$. ON (row (i)) and OFF (row (ii)) steps are defined by the same $c_{X}$ step function as in Fig. 21. Each panel represents a different logic gate — (A) the XOR gate, (B) the AND gate, and (C) the OR gate. For each gate, we select a set of $\left({\bar{K}}_{XZ},{\bar{K}}_{YZ}\right)$ that exhibit unexpected behaviors, and in row (iii) plot the corresponding feed-forward and simple regulation trajectories observed from numerical integration. The $c_{X}$ and $c_{Y}$ signal for these trajectories are the same as in Fig. 21. The XOR gate parameters are ${\bar{r}}_{0Y}={\bar{r}}_{0Z}=0$, ${\bar{r}}_{1Y}={\bar{r}}_{1Z}=2$, and ω=0. The AND gate parameters are ${\bar{r}}_{0Y}={\bar{r}}_{0Z}={\bar{r}}_{1Z}=0$, ${\bar{r}}_{1Y}={\bar{r}}_{2Z}=2$, and ω=10. The OR gate parameters are ${\bar{r}}_{0Y}={\bar{r}}_{0Z}=0$, ${\bar{r}}_{1Y}={\bar{r}}_{1Z}=2$, ${\bar{r}}_{2Z}=10$, and ω=1, which are the same as in Fig. 21.

In Fig. 22, we show a parameter sweep for three sets of rates and cooperativity parameters that correspond to three different logic gates. For each set of parameters, both ON and OFF steps are studied. These calculations inspire several observations. First, for a given step undergone by $p_{\text{act}}^{X}\left(c_{X}\right)$, we can computationally identify a maximum $\langle \Delta \bar{t}\rangle$ with respect to all other parameters. Further, this maximum is different for ON and OFF steps. For example, in Fig. 22(i), specific to the input step chosen here, we observe that in all logic gates the maximal $\langle \Delta \bar{t}\rangle$ achievable is about 1 for the ON step and 4.5 for the OFF step. To tie this back to units of time, we consider *E. coli*. Here, proteins tend to be stable over the timescale of a cell cycle and hence the dilution resulting from cell division becomes the effective degradation rate. Taking a rate of degradation of order $\gamma =10^{-2}\;{\text{min}}^{-1}$, we then get a maximum delay of 500 min, which is more than 8 hours. The asymmetry between maximal $\langle \Delta \bar{t}\rangle$ the ON and OFF steps resonates with the analytic discussion in the previous section. We show computational evidence in Appendix M that sweeps across all other parameters indicate the existence of a global maximum $\langle \Delta \bar{t}\rangle$. Note that the exact value and existence of this maximum is a result specific to function $p_{\text{act}}\left(c_{X}\right)$ and allosteric parameters we chose to describe effector activity in Fig. 5.

Second, both the XOR and AND gates exhibit the expected sign-sensitive delay across a substantial portion of the $\left({\bar{K}}_{XZ},{\bar{K}}_{YZ}\right)$ parameter space, though not uniformly. In particular, Fig. 22(A)(i,ii) shows that for the XOR gate, the lower half of this space yields negligible ON-step delay but a pronounced OFF-step delay. Conversely, Fig. 22(B)(i,ii) reveals that for the AND gate, the upper half of the space yields negligible OFF-step delay and a pronounced ON-step delay. Nevertheless, in specific regions of the $\left({\bar{K}}_{XZ},{\bar{K}}_{YZ}\right)$ parameter space—namely, the upper left quadrant for the XOR gate and the lower right quadrant for the AND gate—neither the ON nor the OFF step exhibits any appreciable delay. More intriguingly, certain extreme choices of dissociation constants contradict the expected behavior: the XOR gate can show delay on both ON and OFF steps, and the AND gate can only delay the OFF step. Example trajectories corresponding to these atypical regimes are shown in Fig. 22(A,B)(iii).

Finally, the OR gate can produce sizable delays, but only for more extreme values of the dissociation constants. Near the region where ${\bar{K}}_{XZ}\approx {\bar{K}}_{YZ}\approx 1$—which corresponds to similar binding strengths between X, Y, and Z with the DNA—the delays for both ON and OFF steps are minimal. This suggests that in some biologically relevant regimes, where dissociation constants are typically of the same order of magnitude, the OR gate is the least effective at generating a temporal delay.

We observe that the average delay $\langle \Delta \bar{t}\rangle$ in coherent feed-forward loops depends strongly on the dissociation constant ${\bar{K}}_{YZ}$, which sets the binding affinity of Y to the promoter of Z—one of the interactions that distinguishes feed-forward loops from simple regulation. A clear trend emerges: ON steps (Fig. 22(A–C)(i)) exhibit stronger delays when Y binding is weak (large ${\bar{K}}_{YZ}$), whereas OFF steps (Fig. 22(A–C)(ii)) show stronger delays when Y binding is strong (small ${\bar{K}}_{YZ}$).

Since the feed-forward loop ultimately aims to control Z activation, another relevant feature is the output amplitude $\text{Δ}\bar{Z}=\left|{\bar{Z}}_{f}-{\bar{Z}}_{i}\right|$ for a given change in input $c_{X}$. A small $\text{Δ}\bar{Z}$ would imply that $\bar{Z}$ remains nearly constant, making the response uninformative. However, we show in Appendix M that $\text{Δ}\bar{Z}$ scales linearly with the production rates. Although the dependence of $\text{Δ}\bar{Z}$ on other parameters is non-trivial, globally the effect of the rates dominates. We thus reserve a detailed discussion of the optimization of $\text{Δ}\bar{Z}$ values for Appendix M and remain focused here on the average delay $\langle \Delta \bar{t}\rangle$.

### c. Existence of pulse in incoherent feed-forward loop

**Figure 23:**
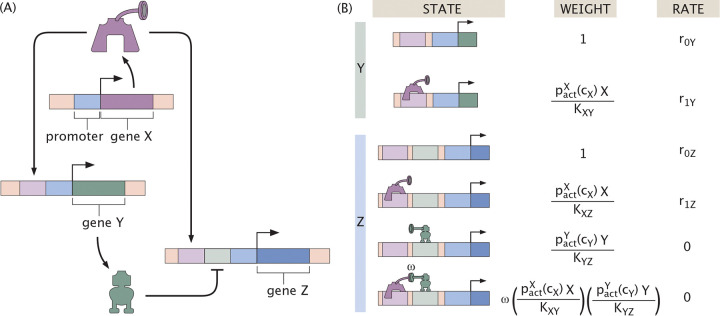
The incoherent feed-forward loop. (A) Schematic representation of the incoherent feed-forward loop. Expression of output protein Z is controlled by expression of protein X, either by direct activation or indirectly, first activating expression of Y which then represses Z. The regulatory circuit is incoherent because the pathways have opposing effects on Z. (B) Thermodynamic states, weights, and rates for expression of repressor Y and output protein Z. X and Y interact with cooperativity ω, but bound repressor suppresses expression regardless of activator presence.

We now turn our attention to the incoherent feed-forward loop depicted in Fig. 23. This architecture is a commonly observed motif in *E. coli*, where X activates both Y and Z, while Y represses Z [97, 98]. This motif gives rise to a qualitatively distinct dynamical behavior from the coherent feed-forward loop: a pulse in the output Z. A pulse is conventionally defined as a transient trajectory of $\bar{Z}(\bar{t})$ such that there exists a time $\bar{t}$ where $\bar{Z}(\bar{t})>{\bar{Z}}_{f}$ if the response of $\bar{Z}(\bar{t})$ is increasing, or $\bar{Z}(\bar{t})<{\bar{Z}}_{f}$ if the response of $\bar{Z}(\bar{t})$ is decreasing. In incoherent feed-forward loops, an ON step signal where $p_{\text{act}}^{X}\left(c_{X}\right)$ increases does not necessarily produce an increasing response in $\bar{Z}(\bar{t})$, as activation induced by increasing $\bar{X}$ competes with repression induced by increasing $\bar{Y}$. This is precisely the incoherence in the name of such circuits.

Referring to the states, weights, and rates shown in Fig. 23, the non-dimensional dynamics of the system are described by

(39) $$\frac{d\bar{Y}}{d\bar{t}}=-\bar{Y}\;+\;\frac{{\bar{r}}_{0Y}\;+\;{\bar{r}}_{1Y}p_{\text{act}}^{X}\bar{X}}{1\;+\;p_{\text{act}}^{X}\bar{X}}$$

and

(40) $$\frac{d\bar{Z}}{d\bar{t}}=-\bar{Z}\;+\;\frac{{\bar{r}}_{0Z}\;+\;{\bar{r}}_{1Z}𝒳}{1\;+\;𝒳\;+\;𝒴\;+\;\omega 𝒳𝒴},$$

with $𝒳=p_{\text{act}}^{X}\left(c_{X}\right)\bar{X}/{\bar{K}}_{XZ}$ and $𝒴=p_{\text{act}}^{Y}\left(c_{Y}\right)\bar{Y}/{\bar{K}}_{YZ}$. Again the bar indicates the time in units of 1/γ, concentrations and dissociation rates in units of $K_{XY}$ and rates in units of $\gamma K_{XY}$.

**Figure 24:**
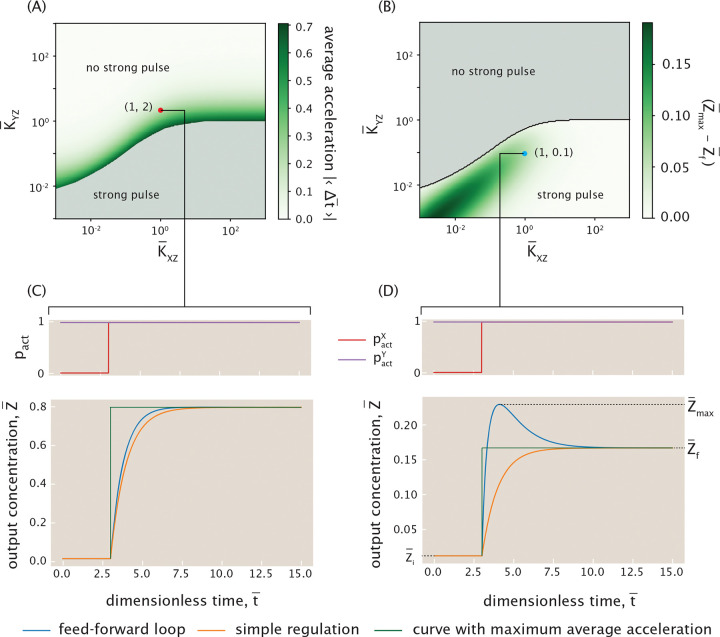
Existence of a pulse and acceleration in the incoherent feed-forward loop. Parameters used here are ${\bar{r}}_{0Y}={\bar{r}}_{0Z}=0$, ${\bar{r}}_{1Y}={\bar{r}}_{1Z}=2$, and ω=0. (A) Average acceleration $\left|\langle \Delta \bar{t}\rangle \right|$ during ON step across the phase space $\left({\bar{K}}_{YZ},{\bar{K}}_{XZ}\right)$, computed only for parameter sets where no strong pulse is observed. For the parameters we chose, the ON step in $p_{\text{act}}^{X}\left(c_{X}\right)$ coincides with $\bar{Z}(\bar{t})$ having an increasing response. (B) Pulse amplitude ${\bar{Z}}_{\max}-{\bar{Z}}_{f}$ across the same phase space, quantifying the transient overshoot above steady state. (C) Example trajectories corresponding to $\left(K_{XZ},K_{YZ}\right)=(1,2)$. No strong pulse is observed, but the feed-forward loop response is accelerated compared to the simple regulation response. The green curve is the trajectory with the largest $\langle \Delta \bar{t}\rangle$ without a pulse. The blue and orange curves are the feed-forward loop and simple regulation trajectories, respectively. (D) Example trajectories corresponding to $\left({\bar{K}}_{XZ},{\bar{K}}_{YZ}\right)=(1,\;0.1)$. A strong pulse is observed.

Intuitively, pulses emerge because of the delay in the repression exerted by Y. Following an ON step in $c_{X}$, $\bar{Y}$ increases exponentially to its final value. In the early phase of the response, X already activates Z strongly, but Y has not yet accumulated enough to exert repression. As a result, $\bar{Z}$ temporarily overshoots its final steady state. This behavior is depicted in Fig. 24(D), where the blue curve exhibits a pronounced pulse, in contrast to the monotonic exponential relaxation of the simple regulation output (orange).

The accelerating nature of the incoherent feed-forward loop can also be captured analytically by repeating the calculation leading to Eqn. 32 but adapted to this new setting. The modified prefactor

(41) $$\text{Φ}=-\left({\bar{r}}_{0Z}\;+\;{\bar{r}}_{1Z}{𝒳}_{f}\right)\left(1\;+\;\omega {𝒳}_{f}\right)$$

is always negative. As a result, the average time difference $\langle \Delta \bar{t}\rangle$ as defined in Eqn. 37 is always positive, both for ON and OFF steps, meaning that the incoherent feed-forward loop accelerates the response for both transitions. In Appendix N, we remark that the definition and interpretation of $\langle \Delta \bar{t}\rangle$ is subtle when $\bar{Z}$ exhibits a pulse. Thus, we only consider $\langle \Delta \bar{t}\rangle$ when a pulse does not exist, and focus on the difference between the peak of the pulse and final steady state of $\bar{Z}$ for the pulses.

To further quantify this acceleration, we again compute the average time difference $\langle \Delta \bar{t}\rangle$ between the feed-forward loop and simple regulation trajectories with Eqn. 37. Fig. 24(A) shows $\langle \Delta \bar{t}\rangle$ across the phase space defined by $\left({\bar{K}}_{XZ},{\bar{K}}_{YZ}\right)$, when the output does not present a strong pulse. The definition of “strong” is described in Appendix N. We observe that acceleration is limited to a maximum value of 1. This upper bound arises because the most accelerated trajectory would consist of an instantaneous jump to the final steady state (green curves in Fig. 24(C) and (D)), corresponding to 〈Δt〉=1 as shown in Appendix N. An example of a feed-forward trajectory that does not exhibit a strong pulse is shown in Fig. 24(C). The region of highest acceleration lies near the boundary separating the strongly pulsed and not strongly pulsed regimes. Interestingly, acceleration is only substantial in a restricted region of parameter space. For example, we see in Fig. 24(A) that high values of ${\bar{K}}_{YZ}$—corresponding to weak binding of Y—lead to negligible acceleration.

In addition to acceleration, the presence and magnitude of a pulse is another hallmark of the studied network. In Fig. 24(B), we map the pulse height, defined as the maximum deviation of $\bar{Z}(t)$ above its steady state value, across the $\left({\bar{K}}_{XZ},{\bar{K}}_{YZ}\right)$ space. Pulses are observed only in a portion of this space—specifically, for small enough values of ${\bar{K}}_{XZ}$, where X binds strongly. The highest pulses occur when both ${\bar{K}}_{XZ}$ and ${\bar{K}}_{YZ}$ are small, indicating that strong binding of both regulators enhances the transient overshoot in this case.

### D. Continuous signal

To conclude our discussion of feed-forward loops, we now consider the system’s response to a continuously tuned effector concentration, rather than an abrupt step change. When the timescale of effector concentration variation, denoted ${\bar{t}}_{c}$, is much shorter than the system’s relaxation timescale, the dynamics resemble those observed under step function inputs. Conversely, when ${\bar{t}}_{c}$ is much longer than the relaxation time, the system remains quasi-stationary when the input is evolving, effectively tracking steady states values of the output $\bar{Z}$ at each time step.

To analyze this quantitatively, we compare ${\bar{t}}_{c}$ to the intrinsic relaxation timescales of both the simple regulation case and the coherent feed-forward loop. In coherent feed-forward loops, we have shown that the output response is consistently delayed relative to simple regulation. However, the precise relaxation timescale of this architecture is not straightforward and depends strongly on the biochemical parameters. As a result, we use the relaxation timescale of the simple regulation system—which is 1 in units of 1/γ—as a reference estimate for the order of magnitude of the feed-forward loop’s relaxation time. We can thus define two limiting regimes: a fast tuning regime where ${\bar{t}}_{c}\ll 1$, and a slow tuning regime where ${\bar{t}}_{c}\gg 1$.

Before proceeding to the analysis of the delay, we must first clarify the definition of simple regulation in the case of a continuous signal. We adopt the same definition as the step function case, where we fix concentration $\bar{Y}=1$ here. Further, in our previous analysis, when $c_{X}(\bar{t})$ is a step function, the choice of $\bar{Y}$ does not affect the dynamics of relaxation of the simple regulation output. However, when $c_{X}(\bar{t})$ is a continuous function, the choice of $\bar{Y}$ changes these dynamics slightly. Fortunately, the effect of $\bar{Y}$ is small and does not qualitatively change our observations in the rest of the section, allowing us to continue with the comparison between feed-forward loop and simple regulation (for a more detailed discussion, see Appendix O).

**Figure 25:**
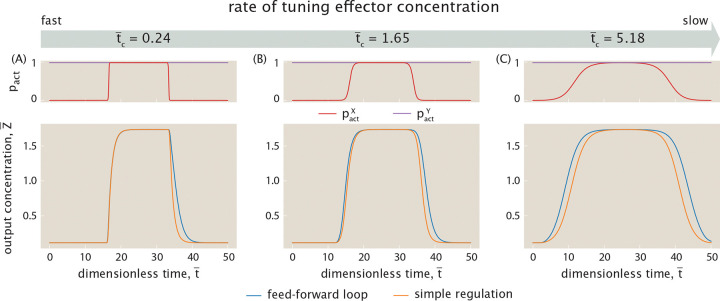
Feed-forward loop response to the rate of continuous tuning of effector concentration. From left to right, the rate of tuning effector concentration slows down while every other parameter is kept constant. Each ON step is a loglinearly increasing function from $c_{X}^{\min}=10^{-4}\;\text{M}$ to $c_{X}^{\min}=10^{-7}\;\text{M}$ across some time; an OFF step is the reverse. From left to right, the timescale of effector concentration variation are ${\bar{t}}_{c}=0.24$, ${\bar{t}}_{c}=1.65$, ${\bar{t}}_{c}=5.18$. ${\bar{t}}_{c}$, as defined in the main text, is the time it takes for $p_{\text{act}}^{X}\left(c_{X}(\bar{t})\right)$ to increase from 0.2 to 0.8. $\bar{Y}$ is set to be 1 at all times for simple regulation. Parameters used are the XOR gate parameters in Fig. 22.

We numerically integrate the dynamical equations under a continuously changing $c_{X}(\bar{t})$ and illustrate the result in Fig. 25, where we analyze the response of a coherent feed-forward loop operating as an XOR gate. In this setting, we numerically define the timescale of effector concentration variation $t_{c}$ as the time it takes for $p_{\text{act}}^{X}\left(c_{X}\right)$ to change from 0.2 to 0.8 or vice versa. $t_{c}$ indicates the time it takes for the switch to be flipped on or off, as regulated by the effector concentration $c_{X}$. When the effector concentration $c_{X}(\bar{t})$ is tuned rapidly as shown in Fig. 25(A), with ${\bar{t}}_{c}\approx 0.24$, we observe a large delay in the output $\bar{Z}(t)$ on the OFF step and almost no delay on the ON step, consistent with the step function dynamics discussed previously. In contrast, when $c_{X}(\bar{t})$ varies slowly, as depicted in Fig. 25(C), with ${\bar{t}}_{c}\approx 5.18$, both the feed-forward loop and the simple regulation trajectories become dominated by their respective steady states. They simply track their steady states dictated by $c_{X}(\bar{t})$. As a consequence, the responses to ON and OFF steps must be symmetric, as there is exactly one steady state corresponding to a specific $c_{X}$. Interestingly, the OFF delay is preserved, while for the ON step the feed-forward loop response is accelerated compared to simple regulation. The magnitude of the ON step acceleration is similar to that of the OFF step delay, as required by symmetry. We note that the magnitude of the acceleration/delay depends on the choice of $\bar{Y}$ in simple regulation.

Nevertheless, for all choices of $\bar{Y}$, the feed-forward loop accelerates the ON step and delays the OFF step when the concentration of effector $c_{X}$ is slowly tuned. Between the two limits, the feed-forward loop response smoothly transitions as the tuning rate of $c_{X}$ decreases. An example in this regime is shown in Fig. 25(B), with ${\bar{t}}_{c}\approx 1.65$, where we begin to observe the ON step acceleration, despite its magnitude being smaller than in Fig. 25(C). These results demonstrate that the dynamics of feed-forward loops under continuously varying effector concentrations are governed by the timescale of input change: rapid tuning reproduces the asymmetric ON- and OFF-step delays seen with step inputs, whereas slow tuning yields symmetric responses where ON-step acceleration balances OFF-step delay.

## V. DISCUSSION

The history of modern molecular biology has been a dazzlingly successful exploration of the way in which genes dictate the function and dynamics of the cells making up organisms of all kinds. One of the greatest success stories of that history is the development of our understanding of how genes are connected together in genetic circuits [14], giving rise to an array of stereotyped regulatory motifs such as switches, oscillators, double negative networks and feed-forward networks, to name but a few examples [97]. We note that despite all of this progress, there remain gaping holes in our knowledge of how most genes are regulated. Even in our best understood organisms such as *E. coli*, we lack any knowledge of how more than 60% of its genes are regulated [99]. As a result, we expect that with the advent of the high-throughput era in biology, ever more genetic circuits like those we discussed here will be discovered.

In addition to our ignorance of the genetic circuits themselves, our understanding of the proteins that mediate those circuits is also very limited. In particular, we often don’t know how effector molecules alter the activity of the transcription factors that control these genes [100]. The key point here is that the action of proteins such as transcription factors is often altered through the binding of effector molecules, which induce allosteric conformational changes that in turn change the state of activity of those transcription factors. However, for many genes, we still remain ignorant of which effector molecules effect those changes. The central thesis of this paper is that, in fact, most gene circuits have their activity tuned by precisely these kinds of effector molecules and as a result, we need to revisit the theoretical analysis of such circuits to account for the effect of allosteric induction.

In parallel with the impressive progress in molecular biology and the dissection of the rules of regulation, huge progress was made in the study of the behavior of dynamical systems in contexts of all kinds [91], and with special reference to genetic circuits themselves [47]. However, when theorists have used dynamical systems frameworks to explore the behavior of such circuits, they have largely adopted an approach in which those circuits are tuned in abstract terms using model parameters such as degradation rates γ, mRNA production rates r and binding constants $K_{d}$. As was so importantly discovered in the 1960s, typically these parameters are in fact “tuned” in the context of living cells through the action of allosteric transitions of transcription factors between active and inactive conformations as a result of the binding of effector molecules [36–43]. The history of dynamical systems in these problems largely leaves the all-important effector molecules out of this story, only considering them implicitly. In this paper we have undertaken a systematic analysis of the role of such effectors in governing the function and dynamics of a variety of fundamental genetic regulatory motifs.

The overarching theme of the work described here is that whereas typical dynamical systems approaches to genetic networks feature the number of transcription factors such as A(t) for activator concentrations and R(t) for repressor concentrations, the variable that the cell actually “cares about” is the active number of activators and repressors. There are a variety of well-defined statistical mechanical approaches that allow us to compute this active fraction by multiplying the total number of transcription factors by the function $p_{\text{act}}(c)$ as dictated by the Monod-Wyman-Changeux model, for example, and highlighted in Eqn. 4. The power of this approach is that now the parameters governing properties such as bistability in genetic circuits will be tuned by experimentally and biologically accessible parameters such as the concentrations of effector molecules.

Throughout the paper, we have shown how the tuning variable of effector concentration makes it possible for the dynamical systems describing genetic circuits to range across their phase portraits. We began with perhaps the simplest of such circuits, the auto-activation motif, and showed how tuning effector concentration narrows the range of possible behaviors relative to those found in an unconstrained dynamical system perspective. We also availed ourselves of the opportunity to compare and contrast the conventional Hill function approach to transcription factor-DNA binding and the more mechanistically detailed thermodynamic models that we systematically explore throughout this work.

One interesting conclusion of this comparison between models based on the full set of states and weights demanded by thermodynamic models and the more phenomenological Hill function is that, for certain parameter regimes, each approach will display dramatically different circuit dynamics (i.e., monostability vs. bistability). This insight emphasizes the need to carefully dissect the quantitative parameters underlying the description of gene regulatory architectures in order to justify whether a Hill function description, which is a limiting case of the thermodynamic description, is warranted.

We also used the auto-activation motif as an opportunity for a careful analysis of the temporal relaxation of these genetic circuits to their terminal steady state. That analysis revealed that for initial conditions that are not “far” from the stable fixed points, the relaxation to steady state is exponential with a time constant dictated by the derivative of the nonlinear protein degradation/production function evaluated at the fixed point. For initial conditions that start near to the unstable fixed point, the dynamics are richer.

With the description of the induced auto-activation circuit in hand, we turned to the very important mutual repression motif which is ubiquitous in prokaryotes and eukaryotes alike. Here, again, the capacity to independently tune the effector concentration for each repressor revealed a large flexibility in how cells and synthetic biologists alike can decide to tune the dynamical behavior of this genetic circuit from dictating its steady state behavior to the dynamics of the repressors as they converge to those steady state values.

Finally, we undertook a dissection of the ubiquitous feed-forward loop. Our analysis shows that the dynamic behavior typically associated with feed-forward loops in response to input effector signals is more nuanced and parameter-dependent than previously appreciated [72]. For the coherent feed-forward loop, we analytically confirm the presence of delay in output response compared to simple regulation. However, we show that both the magnitude and the *sign-sensitivity* of these delays depend on system parameters such as dissociation constants, production rates and cooperativity. This rich range of qualitatively distinct behavior remains true even within the different categories of logic gates that can emerge from special combinations of these parameters. Conversely, incoherent feed-forward loops accelerate output responses compared to simple regulation and can generate transient pulses—again only in certain regions of parameter space.

Within this analysis of feed-forward loops, we demonstrate the crucial roles of effector concentration in our models. The leakiness of $p_{\text{act}}(c)$, for example, influences key metrics such as delay time. We also highlight how the sigmoidal shape of $p_{\text{act}}(c)$ enables a continuous change in effector concentration to be translated to a sharp signal when tuning the probability of transcription factors being active. Overall, while the dynamical behaviors of feed-forward loops can be rich, they are not always guaranteed. This emphasizes how behavior emerges from a delicate interplay of biochemical parameters rather than rigid circuit logic alone, and underscores the need for further experimental and theoretical efforts toward understanding the functions and dynamics of feed-forward loops.

All told, our efforts demonstrate that there is great flexibility inherent in the endogenous signaling modalities adopted by living cells to be contrasted with the way in which model parameters are artificially tuned in many dynamical systems approaches to these same problems. We are excited for experimental efforts to make a substantial push to solve the huge puzzle of the allosterome, opening the door to more realistic analyses of genetic circuits from a dynamical systems perspective.

## Supplementary Material

Supplement 1
