# Supplementary material for: The Dynamics of Inducible Genetic Circuits
Source: ArXiv. 2026 Mar 4:arXiv:2505.07053v2. Preprint. [Version 2] (PMC12976931)
Supplement: Supplement 1 [file NIHPP2505.07053v2-supplement-1.pdf]

Appendix A: Thermodynamic model equivalence to description with polymerase

In this section, we demonstrate the equivalence of the thermodynamic models used throughout the paper in which we essentially ignored the presence of RNA polymerase (RNAP), to those that explicitly incorporate regulatory interaction with the RNA polymerase. To illustrate the comparison between those that explicitly treat polymerase and those that do not, we begin by examining the auto-activation switch with which the paper opened.

1. Coarse-graining the auto-activation model

The statistical mechanical model for auto-activation depicted in Fig. 2 implicitly accounts for interaction between the activator and polymerase. Fig. 26 represents the complete accounting of the thermodynamic states, weights and rates, now explicitly accounting for all of the possible polymerase bound states and denoting the interaction energy between polymerase and activator as ε_{ap}. In light of this complete set of states, weights and rates, We can write the dynamical equation for the number of activators as

$$\frac{dA}{dt} = -\gamma A + \frac{\frac{P}{K_P}[r_0 + 2r_1 e^{-\beta\varepsilon_{ap}} \frac{p_{\mathrm{act}}(c)A}{K_d} + r_2 e^{-2\beta\varepsilon_{ap}} \omega(\frac{p_{\mathrm{act}}(c)A}{K_d})^2]}{Z}, \tag{A1}$$

where P is the number of copies of polymerase present and K_P is the dissociation constant for P. Z is the partition function obtained by summing the statistical weights of all of the states in Fig. 26, which we define as

$$\begin{aligned} Z &= \frac{P}{K_P}\left[1 + 2e^{-\beta\varepsilon_{ap}} \frac{p_{\mathrm{act}}(c)A}{K_d} + e^{-2\beta\varepsilon_{ap}} \omega\Big(\frac{p_{\mathrm{act}}(c)A}{K_d}\Big)^2\right] \\ &\quad + \left[1 + 2\frac{p_{\mathrm{act}}(c)A}{K_d} + \omega\Big(\frac{p_{\mathrm{act}}(c)A}{K_d}\Big)^2\right] \end{aligned}$$

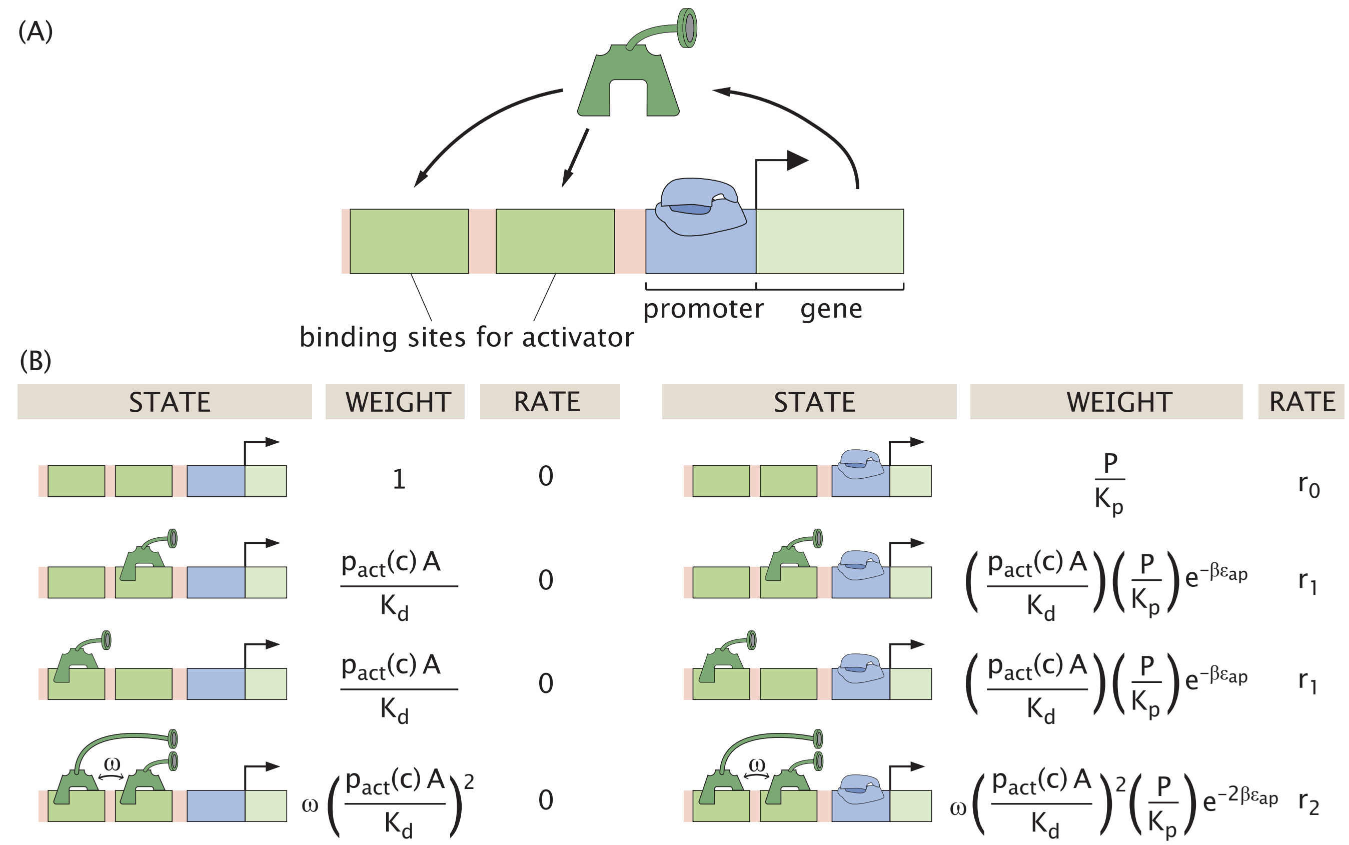

Figure 26: The auto-activation regulatory circuit. (A) A schematic of the circuit operation. Polymerase binding at the promoter (blue) transcribes the gene (encoded in the green region), producing a protein that can activate its own expression at a sufficient concentration. In our model, an activator can bind at two possible sites to enhance gene transcription. (B) The thermodynamic states, weights, and rates for the auto-activation motif including polymerase binding explicitly. The parameter ω denotes the binding cooperativity between two activators.

$$\begin{aligned} &= 1+\frac{P}{K_P}+2\frac{p_{\rm act}(c)A}{K_d}\left(1+\frac{P}{K_P}e^{-\beta\varepsilon_{ap}}\right) \\ &\quad +\omega\left(\frac{p_{\rm act}(c)A}{K_d}\right)^2\left(1+e^{-2\beta\varepsilon_{ap}}\frac{P}{K_P}\right) \\ &= \left(1+\frac{P}{K_P}\right)\left[1+2\frac{p_{\rm act}(c)A}{K_d}\frac{(1+\frac{P}{K_P}e^{-\beta\varepsilon_{ap}})}{1+\frac{P}{K_P}}\right. \\ &\quad \left.+\omega\left(\frac{p_{\rm act}(c)A}{K_d}\right)^2\frac{(1+e^{-2\beta\varepsilon_{ap}}\frac{P}{K_P})}{1+\frac{P}{K_P}}\right] \\ &\equiv \left(1+\frac{P}{K_P}\right)Z_0. \end{aligned} \tag{A2}$$

Note that our goal at this point is to see if by defining the various "constants" that appear in Eqn. A1 we can show that it is equivalent to Eqn. 8 in which we ignored polymerase altogether. In particular, we need to find effective versions of the parameters K_d, ω, r_0, r_1 and r_2 that have all the polymerase dependence hidden within them. To re-express Z_0 as a sum of states with implicit dependence on polymerase, we note that if we define

$$K_d^{\rm eff} = \frac{1+\frac{P}{K_P}}{1+\frac{P}{K_P}e^{-\beta\varepsilon_{ap}}}K_d, \tag{A3}$$

and

$$\omega^{\rm eff} = \frac{(1+\frac{P}{K_P})(1+\frac{P}{K_P}e^{-2\beta\varepsilon_{ap}})}{(1+\frac{P}{K_P}e^{-\beta\varepsilon_{ap}})^2}\omega \tag{A4}$$

then the denominator will have the same form as the denominator of Eqn. 8. Next, we see that if we redefine the rate parameters as

$$r_0^{\rm eff} = \frac{\frac{P}{K_P}}{1+\frac{P}{K_P}}r_0, \tag{A5}$$

$$r_1^{\rm eff} = \frac{\frac{P}{K_P}e^{-\beta\varepsilon_{ap}}}{1+\frac{P}{K_P}e^{-\beta\varepsilon_{ap}}}r_1, \tag{A6}$$

$$r_2^{\rm eff} = \frac{\frac{P}{K_P}e^{-2\beta\varepsilon_{ap}}}{1+\frac{P}{K_P}e^{-2\beta\varepsilon_{ap}}}r_2, \tag{A7}$$

we recover an equation that is equivalent to the dynamical equation as described in Eqn. 8. Note that for convenience, in Eqn. 8 we have everywhere dropped the superscript "eff" because the notation is way too cumbersome to carry throughout the paper. The key point is that we

see that the two approaches are formally equivalent.

However, it is important to always bear in mind that the rate parameters, cooperativity, and dissociation constant used in the reduced representation of the paper are thus effective parameters that implicitly depend on the concentration of polymerase present (P), the strength of polymerase binding to the DNA (K_P), and the strength of interaction between activator and RNAP (ε_{ap}). In a very real sense, this description will lead to a description of the auto-activation switch in which the polymerase serves as a hidden variable. This analysis is extremely interesting because it shows that there is a way to rigorously leave explicit treatment of the polymerase out of the problem.

2. Coarse-graining the mutual repression model

In the main body of the paper, just as we did for the auto-activation motif, we treated the mutual repression motif without making explicit reference to RNA polymerase. We now consider the full set of states, weights and rates illustrated in Fig. 27. The states and weights shown here should be contrasted with those shown in Fig. 14 where all reference to RNA polymerase is absent. We now demonstrate the equivalence of these two descriptions of the mutual repression switch following precisely the same kind of strategy we followed above in the context of the auto-activation switch.

Under this expanded thermodynamic model, the governing equations for the dynamics of R_1 and R_2 prior to non-dimensionalization can be read off directly from Fig. 27 yielding

$$\frac{dR_1}{dt} = -\gamma_1 R_1 + \frac{r\frac{P}{K_P}}{1+\frac{P}{K_P}+2\frac{p_{\rm act}(c_2)R_2}{K_2}+\omega_2\left(\frac{p_{\rm act}(c_2)R_2}{K_2}\right)^2} \tag{A8}$$

and

$$\frac{dR_2}{dt} = -\gamma_2 R_2 + \frac{r\frac{P}{K_P}}{1+\frac{P}{K_P}+2\frac{p_{\rm act}(c_1)R_1}{K_1}+\omega_1\left(\frac{p_{\rm act}(c_1)R_1}{K_1}\right)^2}. \tag{A9}$$

These equations can be algebraically manipulated by factoring out $(1 + P/K_P)$ from the denominator, resulting in the forms

$$\frac{dR_1}{dt} = -\gamma_1 R_1 + \frac{r\frac{\frac{P}{K_P}}{1+\frac{P}{K_P}}}{1+2\frac{p_{\rm act}(c_2)R_2}{K_2(1+\frac{P}{K_P})}+\omega_2(1+\frac{P}{K_P})\left(\frac{p_{\rm act}(c_2)R_2}{K_2(1+\frac{P}{K_P})}\right)^2}, \tag{A10}$$

$$\frac{dR_2}{dt} = -\gamma_2 R_2 + \frac{r\frac{\frac{P}{K_P}}{1+\frac{P}{K_P}}}{1+2\frac{p_{\rm act}(c_1)R_1}{K_1(1+\frac{P}{K_P})}+\omega_1(1+\frac{P}{K_P})\left(\frac{p_{\rm act}(c_1)R_1}{K_1(1+\frac{P}{K_P})}\right)^2}. \tag{A11}$$

This formulation reveals that the original equations given in Eqns. 18 and 19 can be recovered through a simple transformation of parameters in which we once again define effective parameters. The effective mRNA production rate is given by

$$r^{\rm eff} = \frac{\frac{P}{K_P}}{1+\frac{P}{K_P}} r, \tag{A12}$$

the two K_ds for transcription factor-DNA binding are given by

$$K_1^{\rm eff} = (1+\frac{P}{K_P})K_1 \tag{A13}$$

and

$$K_2^{\rm eff} = (1+\frac{P}{K_P})K_2 \tag{A14}$$

and the two cooperativities are written in effective form as

$$\omega_1^{\rm eff} = (1+\frac{P}{K_P})\omega_1 \tag{A15}$$

and

$$\omega_2^{\rm eff} = (1+\frac{P}{K_P})\omega_2. \tag{A16}$$

This demonstrates that polymerase binding can be absorbed into effective parameters, yielding a reduced model equivalent to the one presented in the main text, with renormalized production rate, dissociation constants, and cooperativities. Once again, the polymerase copy number P and binding strength K_P are hidden variables in the context of the bare model, but the results are exact - this is not an approximation valid only in the limit of weak promoters, for example. Note also that as in the case of the auto-activation switch, in the main body of the paper we do *not* carry around the cumbersome "eff" notation, electing instead to simply use the parameters r, K_1, K_2, ω_1 and ω_2 with the convention that those parameters include the hidden variables associated with polymerase.

Appendix B: Minimal bounds on cooperativity and rates for the existence of bistability in auto-activation

The auto-activation system can exhibit bistability, meaning that it can reach different steady states depending on the initial condition. However, this behavior arises only within a restricted range of parameter values, shown in Fig. 11 as the red region. To investigate the conditions for which multiple different steady states are possible, we derive analytic bounds in parameter space. Setting $d\bar{A}/d\bar{t} = 0$ and re-expressing in standard polynomial

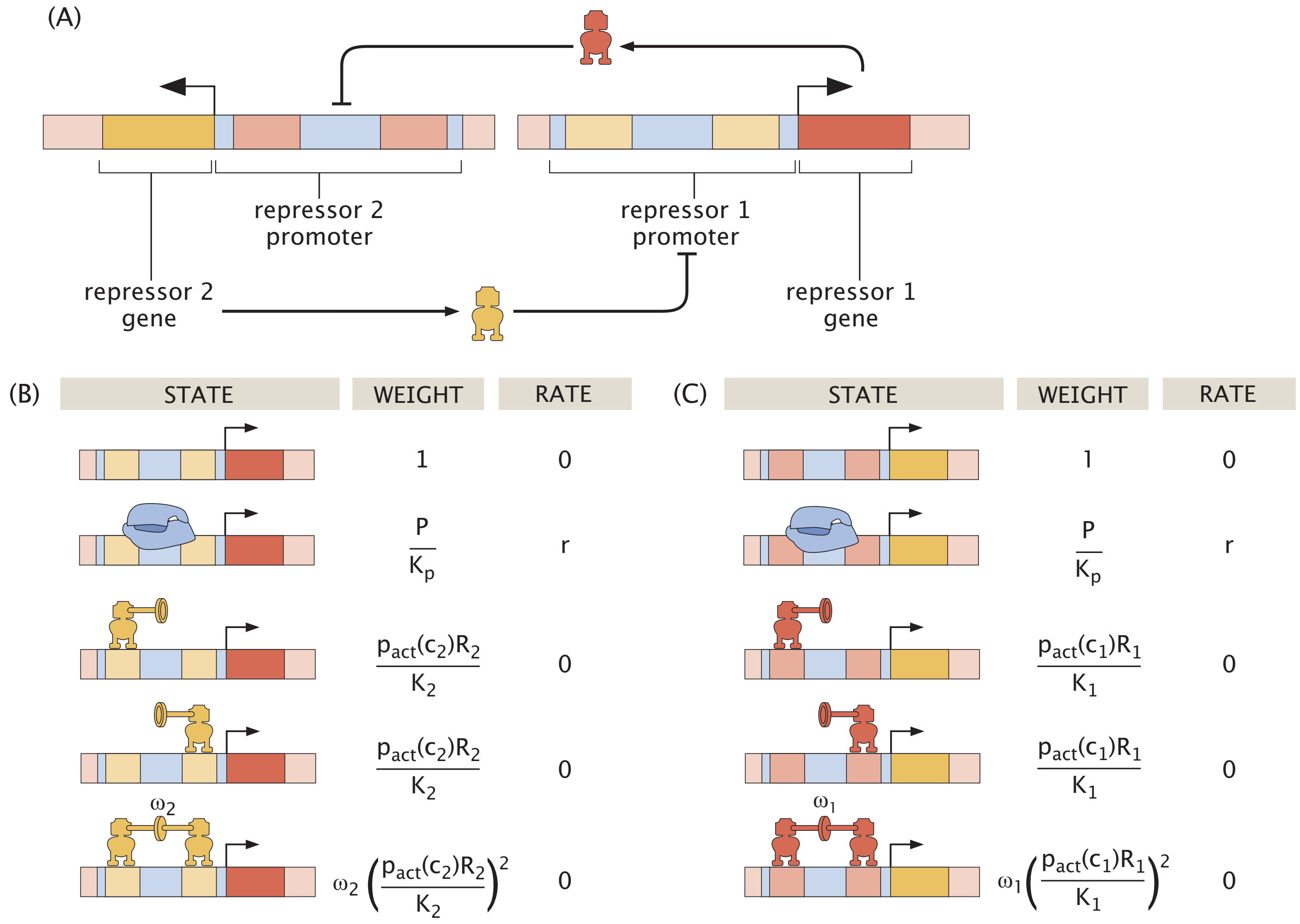

Figure 27: The mutual repression regulatory circuit. (A) Schematic of the operation of the circuit. When the gene for repressor 1 is expressed, the resulting protein downregulates the expression of the gene for repressor 2. Repressor 2, in turn, downregulates the expression of the gene for repressor 1. (B) Thermodynamic states, weights, and rates for expression of repressor 1 including the action of the inducer which tunes the number of active repressors. In our model, a repressor can bind non-exclusively at one of two possible sites within the target promoter region to suppress gene transcription. The parameter ω_2 denotes the cooperative strength between two bound repressors R_2. (C) Thermodynamic states, weights, and rates for expression of repressor 2 including the action of the inducer which tunes the number of active repressors. The states and weights for the regulation of the promoter responsible for the production of repressor 2 are analogous to those shown for repressor 1. However, the dissociation constant of repressor 1 in this case is given by K_1, and the cooperativity term for the interaction of two repressor 1 molecules bound to the DNA is ω_1.

form, $\bar{A}$ must satisfy

$$\omega p_{\mathrm{act}}^2 \bar{A}^3 + p_{\mathrm{act}}(2 - \omega \bar{r}_2 p_{\mathrm{act}})\bar{A}^2 + (1 - 2\bar{r}_1 p_{\mathrm{act}})\bar{A} - \bar{r}_0 = 0. \tag{B1}$$

If the system exhibits bistability, the corresponding polynomial must have three real roots, as a third-order polynomial cannot have exactly two. Physically, these roots correspond to two stable steady states and one unstable steady state. Further, the presence of only one real root indicates that the system is monostable, as discussed in Appendix C.

To identify conditions for bistability, we search for combinations of ω, $\bar{r}0$, $\bar{r}1$, $\bar{r}_2$, and effector concentration c that produce three positive real roots of the polynomial—corresponding to the red region in Fig. 11. We may bound this bistable region of parameter space analytically using Descartes' rule of signs, which states that for a single-variable polynomial with real coefficients, the number of positive roots of the polynomial is equal to the number of sign changes between consecutive non-zero coefficients minus an even number. In our case, the polynomial in Eqn. B1 must then have either one or three sign changes. Therefore, three sign changes are necessary for the system to allow bistability. Evaluating Eqn. B1, we observe that the coefficient of $\bar{A}^3$ is strictly positive and

the constant term is strictly negative. Thus, three (consecutive) coefficient sign changes are only possible if the second term of Eqn. B1 is negative, and the third term of that same equation is positive. Specifically, the condition on the second term implies that

$$p_{\mathrm{act}}(c)\Big(2-\omega\bar{r}_2 p_{\mathrm{act}}(c)\Big)<0 \implies p_{\mathrm{act}}(c)>\frac{2}{\omega\bar{r}_2}, \quad \text{(B2)}$$

while the condition on the second term leads to

$$1-2\bar{r}_1 p_{\mathrm{act}}(c)>0 \implies p_{\mathrm{act}}(c)<\frac{1}{2\bar{r}_1}. \quad \text{(B3)}$$

Thus, these two conditions can be combined to yield

$$\frac{2}{\omega\bar{r}_2}<p_{\mathrm{act}}(c)<\frac{1}{2\bar{r}_1}. \quad \text{(B4)}$$

1. Necessary condition for bistability at some effector concentration c

Note that if the above condition were to be true at all possible effector concentrations c, the system would always be bistable. Rather, we are more specifically interested in the conditions that would allow bistability for at least one value of effector concentration c_0. In other words, there exists a concentration such that

$$\frac{2}{\omega\bar{r}_2}<p_{\mathrm{act}}(c_0) \quad \text{(B5)}$$

and

$$\frac{1}{2\bar{r}_1}>p_{\mathrm{act}}(c_0). \quad \text{(B6)}$$

If the inequality in Eqn. B5 holds true, then we also know that

$$\frac{2}{\omega\bar{r}_2}<p_{\mathrm{act}}(c_0)\le \max_{c\in[0,\infty]}[p_{\mathrm{act}}(c)]. \quad \text{(B7)}$$

This result itself then directly requires the existence of some effector concentration for which Eqn. B5 is true. We can prove this by considering two possible cases. First, if

$$\frac{2}{\omega\bar{r}_2}<\min_{c\in[0,\infty]}[p_{\mathrm{act}}(c)]<\max_{c\in[0,\infty]}[p_{\mathrm{act}}(c)], \quad \text{(B8)}$$

then we know that Eqn. B5 holds true for all concentrations $c\ge 0$. Otherwise, if

$$\min_{c\in[0,\infty]}[p_{\mathrm{act}}(c)]<\frac{2}{\omega\bar{r}_2}<\max_{c\in[0,\infty]}[p_{\mathrm{act}}(c)], \quad \text{(B9)}$$

then Eqn. B5 is true for all non-negative effector concentrations smaller than a threshold concentration

$$c^*=p_{\mathrm{act}}^{-1}\Big(\frac{2}{\omega\bar{r}_2}\Big), \quad \text{(B10)}$$

(derived from the inverse of Eqn. B5) because p_{act} is a continuous and monotonically decreasing function.

Applying similar logic to the inequality in Eqn. B6, we may thus rewrite the necessary conditions for bistability in Eqns. B5 and B6 as

$$\frac{2}{\omega\bar{r}_2} < \max_{c\in[0,\infty]}(p_{\mathrm{act}}(c))=\frac{1}{1+e^{-\beta\varepsilon}}, \quad \text{(B11)}$$

$$\frac{1}{2\bar{r}_1} > \min_{c\in[0,\infty]}(p_{\mathrm{act}}(c))=\frac{1}{1+e^{-\beta\varepsilon}\bar{K}_c^2}, \quad \text{(B12)}$$

and

$$\omega\bar{r}_2>4\bar{r}_1, \quad \text{(B13)}$$

where we have recalled the saturation (maximum) and leakiness (minimum) of $p_{\mathrm{act}}(c)$ defined in Eqns. 6 and 7. Note that we are in a setting where effector binding stabilizes the inactive form of the activator such that $\bar{K}_c=K_A/K_I>1$. This fixes the values of saturation and leakiness, which would otherwise be switched if $K_c<1$. After some algebra, we can re-express Eqns. B11 and B12 such that the necessary conditions for bistability are given by

$$\frac{\omega\bar{r}_2}{2} > 1+e^{-\beta\varepsilon}, \quad \text{(B14)}$$

$$1+e^{-\beta\varepsilon}\bar{K}_c^2 > 2\bar{r}_1, \quad \text{(B15)}$$

and

$$\omega\bar{r}_2>4\bar{r}_1. \quad \text{(B16)}$$

Following a similar procedure as in the previous section, in the next section we derive a necessary condition for bistability that depends on the concentration of effector c. For fixed parameter values, this condition defines a bounded range of effector concentrations outside of which the system is guaranteed to be monostable.

2. Necessary condition for bistability for a fixed concentration of effector c

We consider the case where the activation probability $p_{\mathrm{act}}(c)$ is a decreasing function of the effector concentration c, as seen in Fig. 5. This monotonicity condition, which requires the derivative of the probability function to be negative for all possible c, depends on the parameters of the model, particularly the ratio of dissociation constants $\bar{K}_c$. The derivative of $p_{\mathrm{act}}(c)$ with respect to c is given from Eqn. 4 by

$$\frac{dp_{\mathrm{act}}}{dc}=-\frac{2(1+c/K_A)e^{\beta\varepsilon}(-1+\bar{K}_c)(1+c/K_I)}{\left((1+c/K_A)^2e^{\beta\varepsilon}+(1+c/K_I)^2\right)^2}, \quad \text{(B17)}$$

which is negative for all $c>0$ if and only if $\bar{K}_c>1$.

Recalling the previously-derived necessary condition

for bistability,

$$\frac{2}{\omega\bar{r}_2} < p_{\rm act}(c) < \frac{1}{2\bar{r}_1}, \tag{B18}$$

we now investigate what constraint this condition imposes on the effector concentration c, assuming the parameters of the system are fixed. First, the inequality

$$p_{\rm act}(c) < \frac{1}{2\bar{r}_1} \tag{B19}$$

can be re-expressed equivalently using the explicit expression of $p_{\rm act}(c)$ in Eqn. 4 as

$$\begin{aligned} g(c) = &\left(\frac{c}{K_A}\right)^2 \frac{1}{1+e^{-\beta\varepsilon}}\left[\frac{1}{2\bar{r}_1} - \frac{1}{1+e^{-\beta\varepsilon}\bar{K}_c^2}\right] \\ &+ 2\frac{c}{K_A}\frac{\frac{1}{2\bar{r}_1}(1+e^{-\beta\varepsilon}\bar{K}_c)-1}{(1+e^{-\beta\varepsilon})(1+e^{-\beta\varepsilon}\bar{K}_c^2)} \\ &+ \left[\frac{1}{2\bar{r}_1} - \frac{1}{1+e^{-\beta\varepsilon}}\right]\frac{1}{1+e^{-\beta\varepsilon}\bar{K}_c^2} > 0. \end{aligned} \tag{B20}$$

Similarly, the condition

$$p_{\rm act}(c) > \frac{2}{\omega\bar{r}_2} \tag{B21}$$

is equivalent to requiring that

$$\begin{aligned} h(c) = &\left(\frac{c}{K_A}\right)^2 \frac{1}{1+e^{-\beta\varepsilon}}\left[\frac{2}{\omega\bar{r}_2} - \frac{1}{1+e^{-\beta\varepsilon}\bar{K}_c^2}\right] \\ &+ 2\frac{c}{K_A}\frac{\frac{2}{\omega\bar{r}_2}(1+e^{-\beta\varepsilon}\bar{K}_c)-1}{(1+e^{-\beta\varepsilon})(1+e^{-\beta\varepsilon}\bar{K}_c^2)} \\ &+ \left[\frac{2}{\omega\bar{r}_2} - \frac{1}{1+e^{-\beta\varepsilon}}\right]\frac{1}{1+e^{-\beta\varepsilon}\bar{K}_c^2} < 0. \end{aligned} \tag{B22}$$

We can now apply Descartes' Rule of Signs to the polynomials $g(c)$ and $h(c)$ to determine when the inequalities are satisfied. Since we are working under the assumption that $\bar{K}_c > 1$, this means that,

$$p_{\rm act}^{\max} = \frac{1}{1+e^{-\beta\varepsilon}} > \frac{1}{1+e^{-\beta\varepsilon}\bar{K}_c} > \frac{1}{1+e^{-\beta\varepsilon}\bar{K}_c^2} = p_{\rm act}^{\min}. \tag{B23}$$

For the polynomial $g(c)$, three cases then arise. First, if

$$\frac{1}{2\bar{r}_1} > \frac{1}{1+e^{-\beta\varepsilon}}, \tag{B24}$$

then all coefficients of $g(c)$ are positive and $g(c) > 0$ for all $c \geq 0$, so Eqn. B21 is always satisfied. Second, if

$$\frac{1}{2\bar{r}_1} > \frac{1}{1+e^{-\beta\varepsilon}\bar{K}_c^2}, \tag{B25}$$

then all coefficients are negative, and the condition is never satisfied for any c. Finally, if the intermediate case

$$\frac{1}{1+e^{-\beta\varepsilon}\bar{K}_c} > \frac{1}{2\bar{r}_1} > \frac{1}{1+e^{-\beta\varepsilon}\bar{K}_c^2} \tag{B26}$$

holds, then the coefficient of the term proportional to c^2 in $g(c)$ is positive, while those of the remaining terms proportional to c^1 and c^0 are negative. This results in exactly one sign change, so by Descartes' Rule of Signs, the polynomial $g(c)$ has exactly one positive root. This defines the minimal concentration for bistability, denoted $c_{\rm bistab}^{\min}(\bar{r}_1)$, and given by

$$c_{\rm bistab}^{\min}(\bar{r}_1) = K_A\frac{e^{-\beta\varepsilon}\bar{K}_c + 2\bar{r}_1 - 1 + \sqrt{e^{-\beta\varepsilon}(1+\bar{K}_c)^2(2\bar{r}_1-1)}}{e^{-\beta\varepsilon}\bar{K}_c^2 - 2\bar{r}_1 + 1}. \tag{B27}$$

Under these conditions, the inequality $g(c) > 0$ holds for all $c > c_{\rm bistab}^{\min}(\bar{r}_1)$.

We now turn to the polynomial $h(c)$. If

$$\frac{2}{\omega\bar{r}_2} > \frac{1}{1+e^{-\beta\varepsilon}}, \tag{B28}$$

then all coefficients are positive and the polynomial is strictly positive for all c, meaning that the condition in Eqn. B21 is never satisfied. Conversely, if

$$\frac{2}{\omega\bar{r}_2} < \frac{1}{1+e^{-\beta\varepsilon}\bar{K}_c^2}, \tag{B29}$$

then all coefficients are negative and the condition is always satisfied. Lastly, in the intermediate case

$$\frac{1}{1+e^{-\beta\varepsilon}\bar{K}_c} > \frac{2}{\omega\bar{r}_2} > \frac{1}{1+e^{-\beta\varepsilon}\bar{K}_c^2}, \tag{B30}$$

Descartes' Rule of Signs again implies exactly one positive root of $h(c)$, corresponding to the upper bound of the bistable region. This upper concentration threshold is denoted $c_{\rm bistab}^{\max}(\omega\bar{r}_2)$ and given by

$$c_{\rm bistab}^{\max}(\omega\bar{r}_2) = K_A\frac{e^{-\beta\varepsilon}\bar{K}_c + \frac{\omega\bar{r}_2}{2} - 1 + \sqrt{e^{-\beta\varepsilon}(1+\bar{K}_c)^2\left(\frac{\omega\bar{r}_2}{2}-1\right)}}{e^{-\beta\varepsilon}\bar{K}_c^2 - \frac{\omega\bar{r}_2}{2} + 1}. \tag{B31}$$

Under these conditions, the inequality $h(c) < 0$ holds for all $c < c_{\rm bistab}^{\max}(\omega\bar{r}_2)$.

Summing up the case-by-case analysis, we derive an effector concentration-dependent necessary condition for bistability. The full set of conditions allowing for bistability in different ranges of effector concentrations is given by

$$\begin{cases} c_{\rm bistab}^{\max} > c > c_{\rm bistab}^{\min} & \text{if } \frac{1}{1+e^{-\beta\varepsilon}\bar{K}_c^2} < \frac{2}{\omega\bar{r}_2} < \frac{1}{2\bar{r}_1} < \frac{1}{1+e^{-\beta\varepsilon}}, \\ c_{\rm bistab}^{\max} > c & \text{if } \frac{1}{1+e^{-\beta\varepsilon}\bar{K}_c^2} < \frac{2}{\omega\bar{r}_2} < \frac{1}{1+e^{-\beta\varepsilon}} < \frac{1}{2\bar{r}_1}, \\ c > c_{\rm bistab}^{\min} & \text{if } \frac{2}{\omega\bar{r}_2} < \frac{1}{1+e^{-\beta\varepsilon}\bar{K}_c^2} < \frac{1}{2\bar{r}_1} < \frac{1}{1+e^{-\beta\varepsilon}}, \\ c \geq 0 & \text{if } \frac{2}{\omega\bar{r}_2} < \frac{1}{1+e^{-\beta\varepsilon}\bar{K}_c^2} < \frac{1}{1+e^{-\beta\varepsilon}} < \frac{1}{2\bar{r}_1}, \\ \text{no bistability} & \text{if } \frac{2}{\omega\bar{r}_2} > \frac{1}{1+e^{-\beta\varepsilon}} \text{ or } \frac{1}{1+e^{-\beta\varepsilon}\bar{K}_c^2} > \frac{1}{2\bar{r}_1}. \end{cases} \tag{B32}$$

As noted, the parameter $\bar{r}_0$ does not enter into these Descartes-based bounds and thus does not influence the

existence of bistability in this analysis. From these expressions, we recover the necessary conditions for bistability, stated in the previous section as

$$\frac{2}{\omega \bar{r}_2} < \frac{1}{1+e^{-\beta\varepsilon}}, \tag{B33}$$

$$\frac{1}{1+e^{-\beta\varepsilon}\bar{K}_c^2} < \frac{1}{2\bar{r}_1}, \tag{B34}$$

and

$$\frac{2}{\omega r_2} < \frac{1}{2\bar{r}_1}. \tag{B35}$$

and re-expressed in Eqns. 10 - 12. These conditions can equivalently be rewritten as

$$\omega \bar{r}_2 > \max\left(2(1+e^{-\beta\varepsilon}),\ 4\bar{r}_1\right) \tag{B36}$$

and

$$\bar{r}_1 < \min\left(\frac{1+e^{-\beta\varepsilon}\bar{K}_c^2}{2},\ \frac{\omega\bar{r}_2}{4}\right). \tag{B37}$$

From Eqn. B32, we identify necessary conditions under which the system displays bistability for all effector concentrations above a minimal threshold. This corresponds to being in either the third or fourth case of Eqn. B32. These cases are captured by the inequality

$$\omega\bar{r}_2 > 2(1+e^{-\beta\varepsilon}\bar{K}_c^2), \tag{B38}$$

which implies that, for a sufficiently large product $\omega\bar{r}_2$, the system permits bistability across a semi-infinite range of effector concentrations.

To complement the analytical results summarized in Eqn. B32, we compare the derived necessary conditions for bistability with numerically computed bistability regions across different parameters. As shown in Figure 28, the analytically predicted bounds—represented in orange—are in close agreement with the numerically determined region of bistability—shown in red—near the onset of bistability. For larger values of the cooperativity parameter ω or the activation rate $\bar{r}_2$, as well as for smaller values of the intermediate rate $\bar{r}_1$, the analytical bounds significantly overestimate the true bistable region. This discrepancy arises because the analytical bounds are necessary but not sufficient conditions, and therefore do not capture the full behavior of the system. Nevertheless, these bounds offer a valuable predictor of the minimal and maximal effector concentrations that can support bistability under a given set of parameters.

Appendix C: Fixed point structure of the auto-activation system as a gradient flow

The auto-activation dynamical system defined in Eqn. 9 is a dynamical system that derives from a gradient. Indeed, we can write this equation as

$$\frac{d\bar{A}}{d\bar{t}} = -\frac{dV}{d\bar{A}}, \tag{C1}$$

with

$$-\frac{dV}{d\bar{A}} = \frac{P(\bar{A})}{1+2p_{\text{act}}(c)\bar{A}+\omega(p_{\text{act}}(c)\bar{A})^2}, \tag{C2}$$

and

$$\begin{aligned} P(\bar{A}) &= r_0 + (r_1 2p_{\text{act}}(c)-1)\bar{A} \\ &\quad + (r_2\omega(p_{\text{act}}(c))^2 - 2p_{\text{act}}(c))\bar{A}^2 - \omega(p_{\text{act}}(c))^2\bar{A}^3 \\ &= \omega p_{act}^2(c)(\bar{A}-\bar{A}_1)(\bar{A}-\bar{A}_2)(\bar{A}-\bar{A}_3), \end{aligned} \tag{C3}$$

where $(\bar{A}_1, \bar{A}_2, \bar{A}_3) \in \mathbb{R}$, if there is bistability.

Given Eqn. C2, our goal now is to determine the landscape $V(A)$ itself. To that end, we need to integrate Eqn. C2. We invoke the strategy of separation of variables, resulting in

$$-dV = d\bar{A}\cdot\frac{1+2p_{\text{act}}(c)\bar{A}+\omega(p_{\text{act}}(c)\bar{A})^2}{\omega p_{\text{act}}^2(c)(\bar{A}-\bar{A}_1)(\bar{A}-\bar{A}_2)(\bar{A}-\bar{A}_3)}. \tag{C4}$$

To make progress with this integral, we express the right-hand side using partial fraction decomposition. This yields

$$\frac{1+2p_{\text{act}}(c)\bar{A}+\omega(p_{\text{act}}(c)\bar{A})^2}{\omega p_{\text{act}}^2(c)\prod_{i=1}^3(\bar{A}-\bar{A}_i)} = \sum_{i=1}^{3}\frac{C_i}{\bar{A}-\bar{A}_i}. \tag{C5}$$

We find the coefficients C_1, C_2, and C_3 by multiplying through by the common denominator and evaluating at $\bar{A} = \bar{A}_i$, for $i \in \{1,2,3\}$. The resulting expressions are

$$\begin{cases} C_1 = \dfrac{\frac{1}{p_{\text{act}}^2(c)\omega} + \frac{2}{p_{\text{act}}(c)\omega}\bar{A}_1 + \bar{A}_1^2}{(\bar{A}_1-\bar{A}_2)(\bar{A}_1-\bar{A}_3)}, \\ C_2 = \dfrac{\frac{1}{p_{\text{act}}^2(c)\omega} + \frac{2}{p_{\text{act}}(c)\omega}\bar{A}_2 + \bar{A}_2^2}{(\bar{A}_2-\bar{A}_1)(\bar{A}_2-\bar{A}_3)}, \\ C_3 = \dfrac{\frac{1}{p_{\text{act}}^2(c)\omega} + \frac{2}{p_{\text{act}}(c)\omega}\bar{A}_3 + \bar{A}_3^2}{(\bar{A}_3-\bar{A}_1)(\bar{A}_3-\bar{A}_2)}. \end{cases} \tag{C6}$$

We can then write the potential function $V(\bar{A})$ as

$$V(\bar{A}) = C_1\ln|\bar{A}-\bar{A}_1| + C_2\ln|\bar{A}-\bar{A}_2| + C_3\ln|\bar{A}-\bar{A}_3|. \tag{C7}$$

Since the auto-activation system derives from a gradient, we can apply classical results from one-dimensional gradient dynamics: namely, that the number of stable steady states is equal to the number of unstable steady

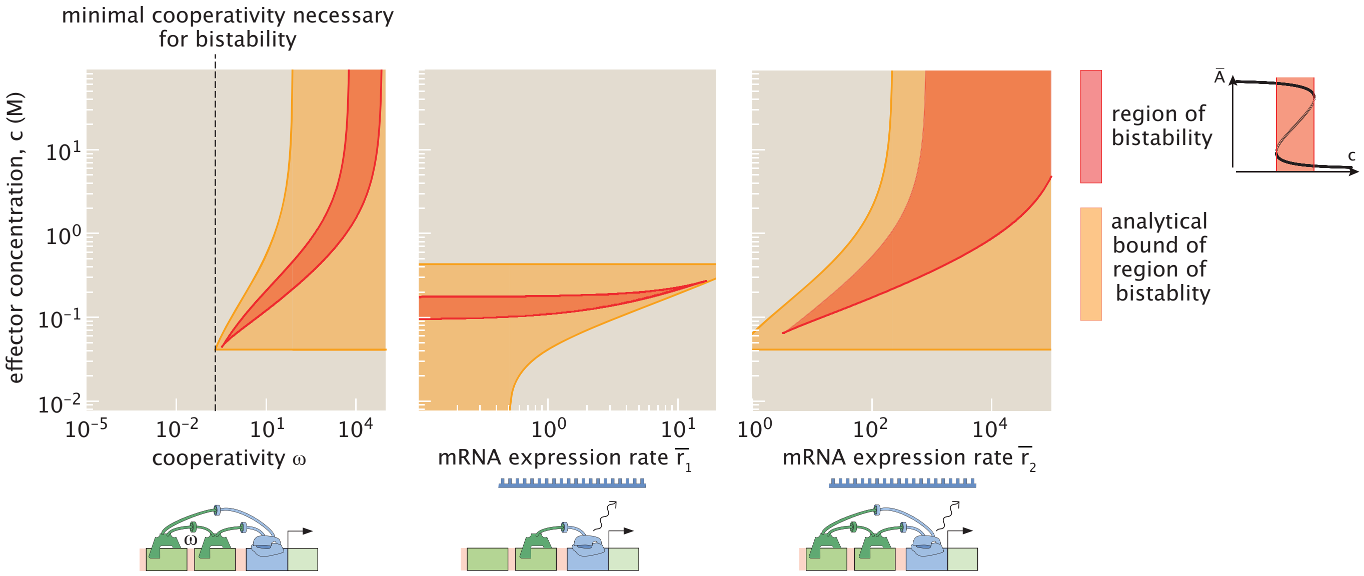

Figure 28: Minimal and maximal values of effector concentration between which the system is bistable. Given baseline parameter values $\omega = 7.5$, $\bar{r}_0 = 0.1$, $\bar{r}_1 = 1$, $\bar{r}_2 = 20$, each figure varies a different parameter, keeping all others fixed. For each panel, the shaded region in red is the region of effector concentration for which there is bistability. The shaded region in orange denotes the analytically-bounded region of bistability. The dotted line in the first figure is an analytical lower bound for the minimal cooperativity required for bistability. Note that the analytic approach discussed here, summed up in Eqn. B32, invokes a necessary but not sufficient condition for bistability, and thus always encompasses a larger region of parameter space than the system's observed region in red.

states plus one [91]. Indeed let's assume that

$$\frac{dV(\bar{A})}{d\bar{A}}|_{\bar{A}=\bar{A}^i} = 0 \tag{C8}$$

at finitely many points $i \in [1, n]$ and

$$\frac{d^2V(\bar{A})}{d\bar{A}^2}|_{\bar{A}=\bar{A}^i} \neq 0 \tag{C9}$$

at those points (the stable points are not degenerate). We take $\bar{A}_1 < ... < \bar{A}_n$. With two minima in V, the function must then reach a local maximum between the two to transition between these minima. We therefore see that the local minima and maxima of V must alternate. A last key point is why that the first and last extrema of V must be minima. If the first extremum of V were a maximum—corresponding to an unstable steady state—a small perturbation toward smaller $\bar{A}$ would drive the system toward the boundary of the domain, where no minimum of V exists and no steady state is defined. This would render the system ill-posed. A similar reasoning can be applied to understand why the last steady state also has to be a minimum. So we can apply this to our system. Intuitively, imagining our dynamical system as a one-dimensional energy landscape, two stable steady state "valleys" must be connected by an unstable steady state "hill." Therefore, bistability implies that our system has three steady states, two stable and one unstable.

Appendix D: Auto-activation : No bistability at high cooperativity and rate $\bar{r}_2$.

As shown in Fig. 11, for sufficiently large values of ω and $\bar{r}_2$, the system does not exhibit bistability for any effector concentration c. In this section, we support this observation using bi-dimensional numerical parameter sweeps and provide analytical arguments explaining its origin.

In Fig. 29, we report the maximal cooperativity ω above which the system is monostable for all values of effector concentration. The rate parameters are varied two at a time while keeping the third fixed. For each triplet $(\bar{r}_0, \bar{r}_1, \bar{r}_2)$, we sample all effector concentrations by sweeping over values of p_{act} between leakiness and saturation. We then determine the maximal value of ω for which the system is bistable for at least one value of c. These parameter sweeps reveal a finite—but potentially large—upper bound on cooperativity beyond which bistability is lost. The yellow regions in Fig. 29(A–B) indicate that no upper bound was found within our sampled cooperativity range (10^1 to 10^9); this absence does not imply the bound does not exist, but rather reflects the limits of our numerical exploration, which did not extend beyond 10^9 due to sampling choices and diminishing biophysical relevance. However, since a finite bound exists in other parts of parameter space, we hypothesize that such a bound also exists in these regions. In the next section, we confirm this analytically. Interestingly, the appearance of yellow regions

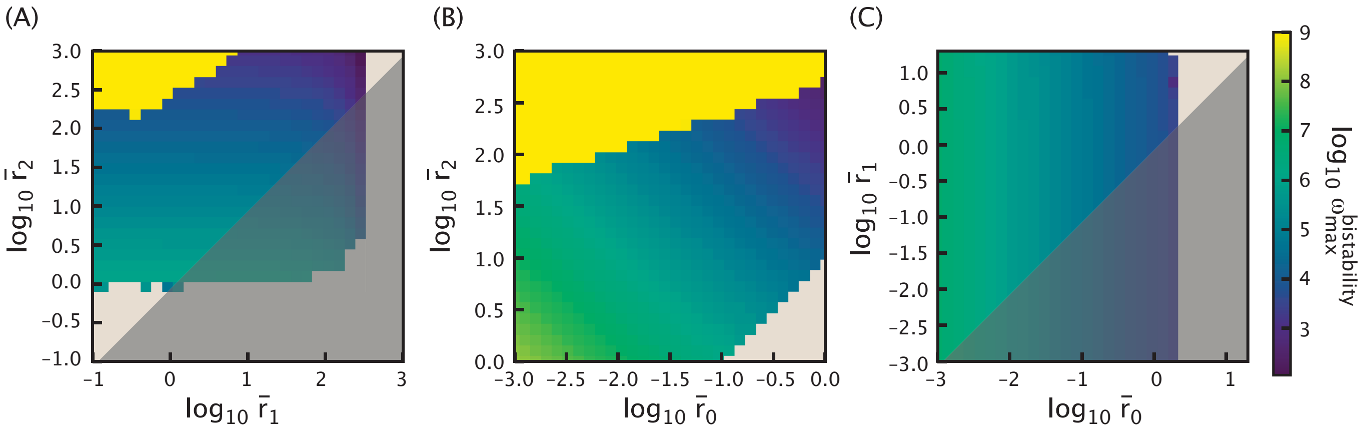

Figure 29: Parameter space exploration of the maximal cooperativity $\omega_{\max}^{\text{bistable}}$ above which the system becomes monostable for all effector concentrations. The cooperativity ω is sampled over the interval $\omega \in [10^1, 10^9]$. Effector concentrations are effectively scanned from 0 to ∞ by sweeping p_{act} between its biologically constrained bounds: the leakiness level $p_{\text{act}}^{\min} = \frac{1}{1+e^{-\beta\varepsilon}\bar{K}_c^2}$ and the saturation level $p_{\text{act}}^{\max} = \frac{1}{1+e^{-\beta\varepsilon}}$, with fixed parameters $\beta\varepsilon = 4.5$ and $\bar{K}_c = 2.6 \times 10^2$. Three two-dimensional parameter sweeps are performed. In panel (A), $(\bar{r}_1, \bar{r}_2)$ are varied in $[\bar{r}_0, 10^5] \times [\bar{r}_0, 10^5]$ with $\bar{r}_0 = 0.1$ held constant. In panel (B), $(\bar{r}_0, \bar{r}_2)$ are varied in $[10^{-5}, \bar{r}_1] \times [\bar{r}_1, 10^5]$ with $\bar{r}_1 = 1$ fixed. In panel (C), $(\bar{r}_0, \bar{r}_1)$ are varied in $[10^{-5}, \bar{r}_2] \times [10^{-5}, \bar{r}_2]$ with $\bar{r}_2 = 20$ fixed. Regions shaded in gray correspond to parameter combinations that violate the auto-activation condition $\bar{r}_0 \leq \bar{r}_1 \leq \bar{r}_2$, and for which the system no longer functions as an auto-activating unit. In regions where no maximal cooperativity values for bistability are reported, the system remains monostable across the entire range of cooperativity values sampled.

in Fig. 29(A–B)—where no numerical upper bound on cooperativity is observed—correlates with increasing values of $\bar{r}_2$, consistent with the fact that raising $\bar{r}_2$ initially promotes bistability. While these bounds appear only at very high cooperativities (typically $\omega > 10^2$), and may exceed biologically plausible values, they nonetheless depend on the system's rate parameters and could be lower in other settings.

We explain this behavior analytically. As discussed in Appendix B 2, bistability can be assessed by examining the number of non-negative roots of the steady-state polynomial Eqn. B1. To be bistable, the system has to admit more than one steady state, which corresponds to the polynomial having three real non-negative roots. A necessary condition for this, is that the polynomial has three real roots, regardless of their sign. While this condition does not guarantee bistability—since some of the roots may be negative—it is nonetheless governed directly by the sign of the polynomial's discriminant. For a general cubic polynomial

$$Q(x) = ax^3 + bx^2 + cx + d, \tag{D1}$$

the discriminant is given by

$$\Delta = b^2c^2 - 4ac^3 - 4b^3d - 27a^2d^2 + 18abcd. \tag{D2}$$

If $\Delta > 0$, the polynomial has three distinct real roots; if $\Delta < 0$, it has only one real root; and if $\Delta = 0$, it has at least one repeated root.

For the auto-activation system, letting $p_{\text{act}}(c) = p$, the discriminant of the steady-state polynomial can be written as

$$\begin{aligned}\Delta = \; & p^2\Big[4 + 32p\,\bar{r}_0 + 16p\,\bar{r}_1(-1 + p\,\bar{r}_1) \\ & + 4\omega(-1 + 2p\,\bar{r}_1)\,(1 + 9p\,\bar{r}_0 + 4p\,\bar{r}_1(-1 + p\,\bar{r}_1)) \\ & - 4\omega p\,(1 + 4p(3\bar{r}_0 + \bar{r}_1(-1 + p\,\bar{r}_1)))\,\bar{r}_2 \\ & - 4\omega^3 p^4 \bar{r}_0\,\bar{r}_2^3 \\ & + \omega^2 p^2\left[-27\,\bar{r}_0^2 + (1 - 2p\,\bar{r}_1)^2\,\bar{r}_2^2 + 6\bar{r}_0\,\bar{r}_2\,(3 - 6p\,\bar{r}_1 + 4p\,\bar{r}_2)\right]\Big].\end{aligned} \tag{D3}$$

We now study the asymptotic behavior of this discriminant in the limits of infinite ω, $\bar{r}_2$, and $\bar{r}_0$. We do not consider the limit of infinite $\bar{r}_1$, since, according to the bounds in Eqns. 10 - 12, a necessary condition for bistability is that $\bar{r}_1$ remains below a threshold set by the other system parameters. Therefore, in this limit, the system is necessarily monostable.

In each of the asymptotic limits, we derive the leading-order term of the discriminant and infer the discriminant diverges negatively. Respectively for ω,

$$\begin{cases} \Delta \underset{\omega\to\infty}{\sim} -4p^6\bar{r}_0\bar{r}_2^3\omega^3, \\ \lim\limits_{\omega\to\infty} \Delta = -\infty, \end{cases} \tag{D4}$$

for $\bar{r}_2$,

$$\begin{cases} \Delta \underset{\bar{r}_2\to\infty}{\sim} -4p^6\bar{r}_0\bar{r}_2^3\omega^3, \\ \lim\limits_{\bar{r}_2\to\infty} \Delta = -\infty, \end{cases} \tag{D5}$$

and for $\bar{r}_0$,

$$\begin{cases} \Delta \underset{\bar{r}_0\to\infty}{\sim} -27\omega^2 p^4 \bar{r}_0^2, \\ \lim\limits_{\bar{r}_0\to\infty} \Delta = -\infty. \end{cases} \tag{D6}$$

These asymptotic results indicate that bistability becomes impossible in the limit of arbitrarily large ω, $\bar{r}_2$, or $\bar{r}_0$.

Since a negative discriminant implies that the polynomial admits only one real root, the system is necessarily monostable in these asymptotic regimes. This analytically supports the existence of upper bounds on ω and $\bar{r}_2$ observed in Fig. 29 and Fig. 11(A,D), consistent with the scaling behaviors shown in Eqns.D4 and D5. Moreover, the symmetric structure of these leading-order terms highlights the seemingly interchangeable roles of ω and $\bar{r}_2$ in promoting bistability. The loss of bistability for large $\bar{r}_0$, as seen in Fig. 11(B), is similarly explained by the negative divergence of the discriminant in Eqn. D6.

Appendix E: Conditions for activation in auto-activation circuit

We define the range of parameters on which we will focus in the setting of the study of auto-activation. In this framework, the production term in Eqn. 9, which we will refer to as $y(\bar{A})$, must be monotonically increasing. In other words, we want $dy/d\bar{A} \geq 0$ for all $\bar{A} \geq 0$. To simplify the computation, let $x = p_{\text{act}}(c)\bar{A}$. We then have

$$\frac{dy}{dx} = \frac{1}{p_{\text{act}}(c)}\frac{dy}{d\bar{A}}, \tag{E1}$$

and the condition thus becomes $dy/dx \geq 0$ for all $x \geq 0$.

Writing down the expression of $y(x)$,

$$y(x) = \frac{\bar{r}_0 + 2\bar{r}_1 x + \omega\bar{r}_2 x^2}{1 + 2x + \omega x^2}, \tag{E2}$$

we compute the derivative

$$\frac{dy}{dx} = \frac{2\left(\bar{r}_1 - \bar{r}_0 + x\omega(\bar{r}_2 - \bar{r}_0) + x^2\omega(\bar{r}_2 - \bar{r}_1)\right)}{\left(1 + x\left(2 + x\omega\right)\right)^2}. \tag{E3}$$

For this expression to be non-negative for all x, it must be non-negative for $x = 0$ and for $x \to \infty$. This then requires that $\bar{r}_2 \geq \bar{r}_1$ and $\bar{r}_1 \geq \bar{r}_0$, further implying that $\bar{r}_2 \geq \bar{r}_0$. This is enough to assert that $y(x)$ is an increasing function of x as its derivative is always non-negative for all $x \geq 0$.

Appendix F: Comparison between the Hill and thermodynamic models for auto-activation

In this section, we provide support for some of the claims made in Section III A 2 when comparing the use of thermodynamic and Hill function approaches to model auto-activation.

First, we demonstrate how a Hill function emerges in the high cooperativity limit of the thermodynamic model, a result known previously in the literature [55]. To reiterate, the dynamical equation for protein production through auto-activation using a thermodynamical model is

$$\frac{dA}{dt} = -\gamma A + \frac{r_0 + r_1(2p_{\text{act}}(c)\frac{A}{K_d}) + r_2\omega(p_{\text{act}}(c)\frac{A}{K_d})^2}{1 + 2p_{\text{act}}(c)\frac{A}{K_d} + \omega(p_{\text{act}}(c)\frac{A}{K_d})^2}. \tag{F1}$$

Letting $K_d^{\text{eff}} = K_d/\sqrt{\omega}$, we can then re-express the previous equation as

$$\frac{dA}{dt} = -\gamma A + \frac{r_0 + r_1 2p_{\text{act}}(c)\frac{A}{K_d^{\text{eff}}\sqrt{\omega}} + r_2(p_{\text{act}}(c)\frac{A}{K_d^{\text{eff}}})^2}{1 + 2p_{\text{act}}(c)\frac{A}{K_d^{\text{eff}}\sqrt{\omega}} + (p_{\text{act}}(c)\frac{A}{K_d^{\text{eff}}})^2}. \tag{F2}$$

In the limit as $\omega \to \infty$ with finite K_d^{eff}, the single activator-bound state vanishes and the dynamics simplify to

$$\frac{dA}{dt} = -\gamma A + \frac{r_0 + r_2(p_{\text{act}}(c)\frac{A}{K_d^{\text{eff}}})^2}{1 + (p_{\text{act}}(c)\frac{A}{K_d^{\text{eff}}})^2}. \tag{F3}$$

The production term thus takes the form of a Hill function with $n = 2$, as in Eqn. 14.

We next compare the probabilities of different state occupancies as derived from the thermodynamic and Hill function models. Fig. 30 plots the probabilities of no TF bound, one TF bound, and two TFs bound as functions of the active activator concentration $A_{\text{act}} = p_{\text{act}}(c)A$. In the thermodynamic model the probability of no activator bound is

$$\frac{1}{1 + 2\frac{A_{\text{act}}}{K_d} + \omega\left(\frac{A_{\text{act}}}{K_d}\right)^2}. \tag{F4}$$

In the high cooperativity regime, the singly-bound state has negligible weight, and the probabilities of zero or two activators being bound, as plotted in Fig. 30(B), closely match those given by the Hill function in Fig. 30(D). The correspondence is weaker in the low cooperativity regime but still visible. The probabilities of zero or two activators being bound, plotted in Fig. 30(A) and (C) for both models, share a sigmoidal shape and have similar EC50. Where the probability curves intersect at $A_{\text{act}} = 1\,\mu$M, however, the Hill model assigns higher probabilities than the thermodynamic model because the singly bound

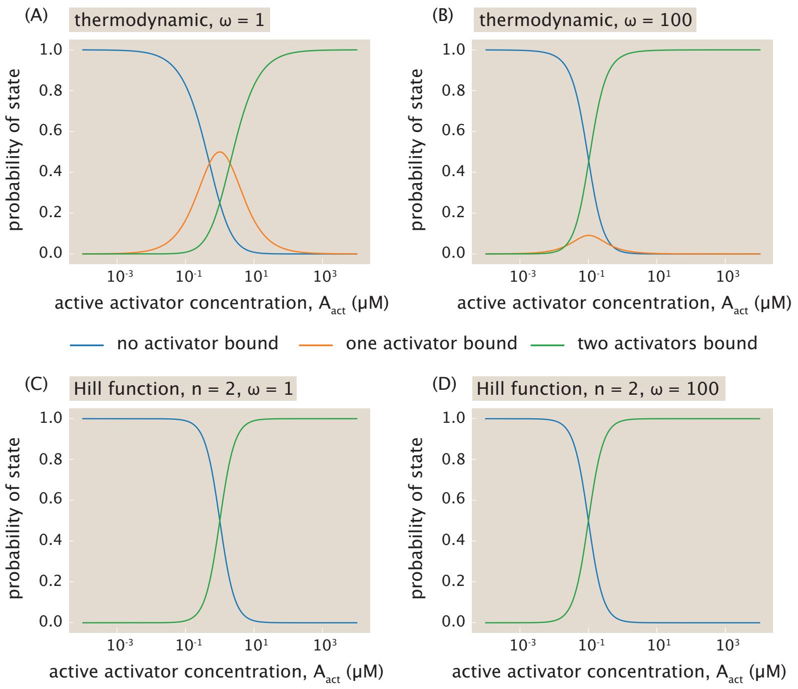

Figure 30: Comparing probabilities of activator binding states as a function of active activator concentration $A_{\rm act}$ in the thermodynamic and Hill model. $K_d = 1\mu$M across all panels. (A) Thermodynamic model with cooperativity $\omega = 1$. (B) Thermodynamic model with $\omega = 100$. (C) Hill function model with $\omega = 1$. (D) Hill function model with $\omega = 100$. The blue curve corresponds to the state with no activator bound. The orange curve corresponds to the state with one activator bound. The green curve corresponds to the state with two activators bound. Notably, there is no orange curve in (C) as the Hill function approximates away the state with one activator bound.

state contributes strongly in the latter setting.

Overall, these differences appear moderate, and it is difficult to anticipate from these results alone the substantive differences between the two models in the small ω regime that we present in the main text. This highlights the importance of comparing the two models not only through their production curves but also through their bifurcation curves.

Appendix G: Oligomerization and DNA Looping in the auto-activation regulation unit

In the main text, transcription factors (TFs) are treated as single binding units that occupy one site at a time, with cooperative interactions between TFs bound at two neighboring sites on the DNA. This description, however, omits two important possibilities: (i) TFs may oligomerize before binding DNA (for example as dimers, like the transcription factor PhoP in *E. coli* [101], or as tetramers, like the transcription factor ComK [102] in *B. subtilis*); and (ii) they may bridge distant sites to form DNA loops [30, 31, 103, 104]. Here we consider how these features would change the behavior of an auto-activating transcription factor, with similar analysis possible for mutual repression and other regulatory motifs.

To isolate the effects of oligomerization, the following discussion does not include inducers, though in general they could interact differently with monomers and oligomers. To extend the model, Eqn. 8 in Section III A, we now allow the transcription factor to dimerize, and each protein copy to exist as a free monomer or as part of a dimer. We track the total concentration of monomer subunits as

$$A_{\rm tot} = A + 2A_2, \tag{G1}$$

where A_2 denotes dimers. Protein synthesis increases $A_{\rm tot}$, and assuming rapid dimerization the newly produced monomers are instantly redistributed between monomers and dimers according to equilibrium. We assume that dilution and degradation act at the same effective rate γ on both monomers and dimers, which is reasonable for stable TFs in fast-growing *E. coli* where dilution dominates, as a few candidates of auto-activating feedback loops are present in this organism [105, 106].

Assuming the reaction $2A \leftrightarrow A_2$ is much faster than synthesis or degradation, the dimer concentration is given by

$$A_2 = \frac{A^2}{K_{\rm dim}}. \tag{G2}$$

Mass balance then gives

$$A_{\rm tot} = A + \frac{2A^2}{K_{\rm dim}}, \tag{G3}$$

which, solving for A, yields an explicit definition for the free monomer concentration as a function of $A_{\rm tot}$,

$$A(A_{\rm tot}) = \frac{K_{\rm dim}}{4}\left(\sqrt{1+\frac{8A_{\rm tot}}{K_{\rm dim}}}-1\right), \tag{G4}$$

and, given Eqn. G5, the corresponding free dimer concentration as a function of $A_{\rm tot}$,

$$A_2(A_{\rm tot}) = \frac{A(A_{\rm tot})^2}{K_{\rm dim}}. \tag{G5}$$

The transcription factor can bind DNA either as a monomer, with dissociation constant K_d^m, or as a dimer, with dissociation constant K_d^d. We now consider a promoter with two identical binding sites. The binding of two proteins can be cooperative, described by cooperativity parameters ω_{mm} (monomer–monomer), ω_{md} (monomer–dimer), and ω_{dd} (dimer–dimer). When both sites are occupied by dimers, they can also bridge the two sites to form a DNA loop, with a dissociation constant K_d^l describing the dimer bound to DNA in its looped state. Each promoter state is associated with a transcription rate r_0, r_1, or r_2, corresponding to zero, one, or two bound proteins, respectively. Given the statistical weights of all promoter states as presented in Fig. 31, the mean transcription rate is then

$$\begin{aligned} F(A, A_2) = {} & \frac{r_0}{Z} + r_1\frac{2\frac{A}{K_d^m}+2\frac{A_2}{K_d^d}}{Z} \\ & + r_2\frac{\omega_{mm}\left(\frac{A}{K_d^m}\right)^2 + 2\omega_{md}\frac{A}{K_d^m}\frac{A_2}{K_d^d} + \omega_{dd}\left(\frac{A_2}{K_d^d}\right)^2}{Z} \\ & + r_2\frac{\frac{A_2}{K_d^l}}{Z}, \end{aligned} \tag{G6}$$

with a partition function

$$\begin{aligned} Z = {} & 1 + 2\frac{A}{K_d^m} + 2\frac{A_2}{K_d^d} + \omega_{mm}\left(\frac{A}{K_d^m}\right)^2 + 2\omega_{md}\frac{A}{K_d^m}\frac{A_2}{K_d^d} \\ & + \omega_{dd}\left(\frac{A_2}{K_d^d}\right)^2 + \frac{A_2}{K_d^l}, \end{aligned} \tag{G7}$$

and the corresponding dynamics of the system become

$$\frac{dA_{\rm tot}}{dt} = -\gamma A_{\rm tot} + F\big(A(A_{\rm tot}), A_2(A_{\rm tot})\big). \tag{G8}$$

Eqn. G8 shows that once oligomerization is included, the system must be described in terms of $A_{\rm tot}$ rather than the free monomer alone. Importantly, the dynamics become

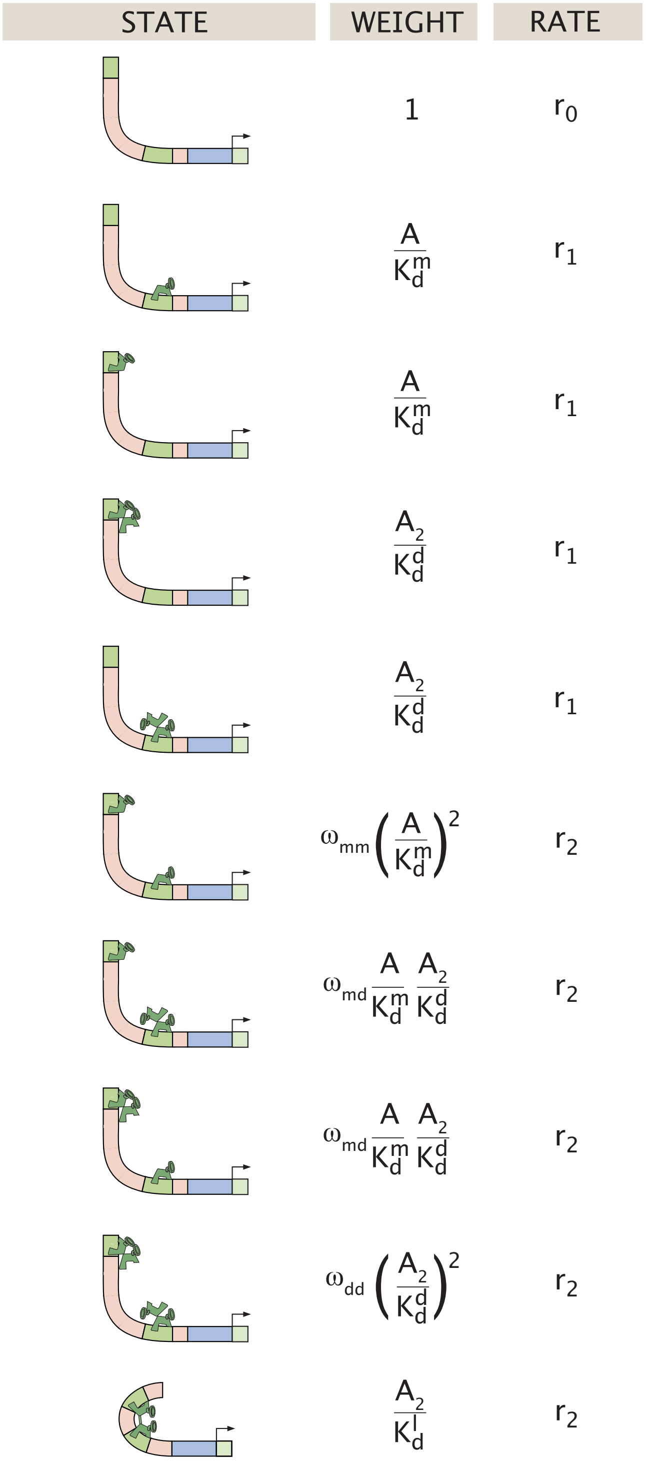

Figure 31: Promoter states for a dimerizing auto-activating transcription factor. Each row shows a possible promoter occupancy state when two binding sites are available. States differ by the number and type of bound transcription factor (monomer or dimer) and by whether the two dimers form a DNA loop. Each state is associated with a statistical weight determined by its binding configuration and a transcription rate (r_0, r_1, or r_2) that depends on the number of bound molecules.

more nonlinear, allowing new behaviors to emerge.

We now consider an alternative setting in which several oligomeric forms coexist in solution, but only one can bind to DNA. As an example, suppose that monomers and dimers are found in solution with equilibrium constant K_{dim}, but only monomers can bind to the gene promoter site. We then have

$$\frac{dA_{\text{tot}}}{dt} = -\gamma A_{\text{tot}} + \frac{r_0 + 2r_1\frac{A}{K_d^m} + r_2\omega_{mm}(\frac{A}{K_d^m})^2}{1 + 2\frac{A}{K_d^m} + \omega_{mm}(\frac{A}{K_d^m})^2}. \quad \text{(G9)}$$

Substituting $A_{\text{tot}} = A + 2A^2/K_{\text{dim}}$ and using the chain rule, we can ultimately arrive at an ordinary differential equation with additional terms beyond those of the simple monomer case,

$$\begin{aligned}\frac{dA}{dt} =& -\gamma\frac{K_{\text{dim}}A + 2A^2}{K_{\text{dim}} + 4A}\\ &+ \frac{K_{\text{dim}}}{K_{\text{dim}} + 4A}\frac{r_0 + 2r_1\frac{A}{K_d^m} + r_2\omega_{mm}(\frac{A}{K_d^m})^2}{1 + 2\frac{A}{K_d^m} + \omega_{mm}(\frac{A}{K_d^m})^2}.\end{aligned} \quad \text{(G10)}$$

The presence of multiple oligomeric states could therefore alter the number and stability of steady states, although in practice usually only one oligomeric form is dominant.

We next consider looping when only the dimeric form of the transcription factor is present. If a dimer both binds and loops DNA, looping can be seen as adding binding configurations that strengthen effective binding and cooperativity. The production term of a looping dimer A_2 can then be written as

$$\frac{r_0 + r_1(2 + \frac{K_d^d}{K_d^l})p_{\text{act}}\frac{A_2}{K_d^d} + r_2\omega_{dd}\left(\frac{p_{\text{act}}A_2}{K_d^d}\right)^2}{1 + (2 + \frac{K_d^d}{K_d^l})p_{\text{act}}\frac{A_2}{K_d^d} + \omega_{dd}\left(\frac{p_{\text{act}}A_2}{K_d^d}\right)^2}, \quad \text{(G11)}$$

which is algebraically equivalent to the standard model using renormalized parameters

$$\tilde{K_d^d} = \frac{2K_d^d}{2 + \frac{K_d^d}{K_d^l}} \quad \text{(G12)}$$

and

$$\tilde{\omega_{dd}} = \frac{4\omega_{dd}}{(2 + \frac{K_d^d}{K_d^l})^2}. \quad \text{(G13)}$$

Looping therefore does not change the functional form of the model but simply rescales K_d^d and ω_{dd}.

Finally, when dimers form from monomers with equilibrium constant K_{dim}, and there is no looping and all cooperativity terms are equal ($\omega_{mm} = \omega_{md} = \omega_{dd} = \omega$), the production term can be reduced exactly to the monomer-only functional form as

$$f_{\text{mono}}(x) = \frac{r_0 + 2r_1x + r_2\,\omega x^2}{1 + 2x + \omega x^2}, \quad \text{(G14)}$$

where

$$x = \frac{A}{K_d^m} + \frac{A_2}{K_d^d}. \quad \text{(G15)}$$

The dynamical equation can then be written as

$$\frac{dA_{\text{tot}}}{dt} = -\gamma\,A_{\text{tot}} + f_{\text{mono}}\big(x(A_{\text{tot}})\big). \quad \text{(G16)}$$

At steady state this means that

$$\gamma(x)x = f_{\text{mono}}(x), \quad \text{(G17)}$$

with

$$\gamma(x) = \frac{2K_d^d\left(K_d^d - K_d^m\left(K_{dim} + \sqrt{K_d^d\left(\frac{K_d^d}{(K_d^m)^2} + 4x\right)}\right)\right)}{K_d^d\,(1 - 2K_{dim}) - K_d^m\sqrt{K_d^d\left(\frac{K_d^d}{(K_d^m)^2} + 4x\right)}}. \quad \text{(G18)}$$

Thus, the production curve retains the same shape as in the monomeric model, but the effective degradation rate becomes concentration-dependent.

The various settings discussed in this appendix demonstrate how incorporating oligomerization alters the equations of the dynamical system. Specifically, accounting for oligomerization introduces additional molecular states, and makes the effective production term a function of the total protein pool rather than only the active monomer. This transforms the dynamics from a simple one-variable system into a more nonlinear one. By contrast, looping does not change the mathematical structure of the model but can be absorbed into effective parameters that strengthen binding and cooperativity. When only a single oligomeric form binds DNA, the production term can still be written in the same functional form as the monomer-only model, but the degradation term becomes effectively concentration-dependent.

Appendix H: Auto-activation : Bistability is possible for non cooperative systems ($\omega = 1$).

1. Definition of the effective Hill coefficient

We first recall how to compute the Hill coefficient of an activating Hill function with constitutive expression, denoted $g(x)$, defined by

$$g(x) = \frac{\bar{r}_0 + \bar{r}_2x^n}{1 + x^n}. \quad \text{(H1)}$$

Its log-derivative is

$$\frac{d\ln g}{d\ln x} = n \cdot \frac{x^n(\bar{r}_2 - \bar{r}_0)}{(1+x^n)(\bar{r}_0 + \bar{r}_2 x^n)}. \quad \text{(H2)}$$

Let us define x^* such that

$$g(x^*) = \frac{\bar{r}_2 + \bar{r}_0}{2}. \quad \text{(H3)}$$

This holds for $x^* = 1$. Evaluating the derivative at this value then gives

$$\left.\frac{d\ln g}{d\ln x}\right|_{x=x^*} = \frac{1}{2} n \cdot \frac{\bar{r}_2 - \bar{r}_0}{\bar{r}_2 + \bar{r}_0}, \quad \text{(H4)}$$

and solving for n, we obtain

$$n = \left.\frac{d\ln g}{d\ln x}\right|_{x=x^*} \cdot 2\frac{\bar{r}_2 + \bar{r}_0}{\bar{r}_0 - \bar{r}_2}. \quad \text{(H5)}$$

We now define a similar expression for a thermodynamic model $w(x)$ given by

$$w(x) = \frac{\bar{r}_0 + 2\bar{r}_1 p_{\mathrm{act}} x + \omega \bar{r}_2 p_{\mathrm{act}}^2 x^2}{1 + 2p_{\mathrm{act}} x + \omega p_{\mathrm{act}}^2 x^2}. \quad \text{(H6)}$$

We observe that $w(x)$ can be written as $\hat{w}(p_{\mathrm{act}} x)$ with

$$\hat{w}(\hat{x}) = \frac{\bar{r}_0 + 2\bar{r}_1 \hat{x} + \omega \bar{r}_2 \hat{x}^2}{1 + 2h\hat{x} + \omega \hat{x}^2}, \quad \text{(H7)}$$

where $h = 1$ in our case. Since $d\ln w/d\ln x = d\ln\hat{w}/d\ln\hat{x}$ when $h = 1$, the effective Hill coefficient does not depend on p_{act}. The derivative of $\hat{w}$ is

$$\frac{d\ln\hat{w}}{d\ln\hat{x}} = \frac{2(1+h\hat{x})}{1+\hat{x}(2h+\omega\hat{x})} - \frac{2(\bar{r}_0 + \bar{r}_1\hat{x})}{\bar{r}_0 + \hat{x}(2\bar{r}_1 + \omega\bar{r}_2\hat{x})}. \quad \text{(H8)}$$

Letting $\hat{x}^*$ be defined by $\hat{w}(\hat{x}^*) = \frac{\bar{r}_2+\bar{r}_0}{2}$ yields

$$\hat{x}^* = \frac{-h\bar{r}_0 + 2\bar{r}_1 - h\bar{r}_2}{(\bar{r}_0 - \bar{r}_2)\omega} + \sqrt{S}, \quad \text{(H9)}$$

where

$$S = \frac{\begin{array}{c} h^2\bar{r}_0^2 - 4h\bar{r}_0\bar{r}_1 + 4\bar{r}_1^2 + 2h^2\bar{r}_0\bar{r}_2 \\ - 4h\bar{r}_1\bar{r}_2 + h^2\bar{r}_2^2 + \bar{r}_0^2\omega - 2\bar{r}_0\bar{r}_2\omega + \bar{r}_2^2\omega \end{array}}{(\bar{r}_0 - \bar{r}_2)^2\omega^2}. \quad \text{(H10)}$$

This then gives the derivative at $\hat{x}^*$ as

$$\left.\frac{d\ln\hat{w}}{d\ln\hat{x}}\right|_{\hat{x}=\hat{x}^*} = \frac{\bar{r}_2 - \bar{r}_0}{\bar{r}_2 + \bar{r}_0} \cdot \frac{(h(\bar{r}_0 + \bar{r}_2) - 2\bar{r}_1)u + t}{(2h\bar{r}_2 - 2\bar{r}_1)u + t}, \quad \text{(H11)}$$

with

$$\begin{cases} \alpha = \sqrt{\frac{(-2\bar{r}_1 + h(\bar{r}_0 + \bar{r}_2))^2}{(\bar{r}_0 - \bar{r}_2)^2} + 4\omega}, \\ u = -2\bar{r}_1 + h(\bar{r}_0 + \bar{r}_2) + (-\bar{r}_0 + \bar{r}_2)\alpha, \\ t = 4(\bar{r}_0 - \bar{r}_2)^2\omega. \end{cases} \quad \text{(H12)}$$

We defined $\hat{w}$ with an extra parameter h to allow a mapping between the thermodynamic model and the Hill function. For the Hill case, we set $h = 0$, $\bar{r}_1 = 0$, and $\omega = 1$. In our thermodynamic model, $h = 1$ and the expression of Eqn. H11 becomes

$$\left.\frac{d\ln\hat{w}}{d\ln\hat{x}}\right|_{\hat{x}=\hat{x}^*} = \frac{\bar{r}_2 - \bar{r}_0}{\bar{r}_2 + \bar{r}_0} \cdot \frac{(\bar{r}_0 + \bar{r}_2 - 2\bar{r}_1)u + t}{(2\bar{r}_2 - 2\bar{r}_1)u + t}, \quad \text{(H13)}$$

with

$$\begin{cases} \alpha = \sqrt{\frac{(-2\bar{r}_1 + \bar{r}_0 + \bar{r}_2)^2}{(\bar{r}_0 - \bar{r}_2)^2} + 4\omega}, \\ u = -2\bar{r}_1 + \bar{r}_0 + \bar{r}_2 + (-\bar{r}_0 + \bar{r}_2)\alpha, \\ t = 4(\bar{r}_0 - \bar{r}_2)^2\omega. \end{cases} \quad \text{(H14)}$$

To ensure consistency with the Hill model case, we therefore define the effective Hill coefficient as

$$n_{\mathrm{eff}} = \left.\frac{d\ln w}{d\ln x}\right|_{x=x^*} \cdot 2\frac{\bar{r}_2 + \bar{r}_0}{\bar{r}_2 - \bar{r}_0} \quad \text{(H15)}$$

for

$$w(x) = \frac{\bar{r}_0 + 2\bar{r}_1 p_{\mathrm{act}} x + \omega \bar{r}_2 p_{\mathrm{act}} x^2}{1 + 2p_{\mathrm{act}} x + \omega p_{\mathrm{act}} x^2} \quad \text{(H16)}$$

and x^* defined such that

$$w(x^*) = \frac{\bar{r}_2 + \bar{r}_0}{2}. \quad \text{(H17)}$$

We note that for the auto activation system, in which $\bar{r}_0 \le \bar{r}_1 \le \bar{r}_2$, having an effective Hill coefficient larger than unity ($n_{eff} > 1$) is equivalent to

$$\omega > \frac{(\bar{r}_1 - \bar{r}_0)(\bar{r}_2 - \bar{r}_1)}{(\bar{r}_2 - \bar{r}_0)^2}. \quad \text{(H18)}$$

Therefore, depending on the parameters of the system, the effective Hill coefficient can be larger or smaller than one, despite the activator having two binding sites.

2. Numerical sweep for minimal cooperativity above which there is bistability.

To better understand how the parameters of the auto-activation system constrain the emergence of bistability, we explore the minimal cooperativity $\omega_{\mathrm{min}}^{\mathrm{bistable}}$ required to observe bistability across a broad range of rate parameters. Specifically, we perform numerical parameter sweeps over $(\bar{r}_0, \bar{r}_1, \bar{r}_2)$, systematically enforcing the auto-activation condition $\bar{r}_0 \le \bar{r}_1 \le \bar{r}_2$. Regions where this condition is violated are shaded in gray. For each valid triplet, we determine the minimal value of ω for which bistability occurs over a finite range of effector concentrations.

Fig. 32(A) illustrates the numerical method used to identify this minimum cooperativity. The resulting values are displayed in Fig. 32(B), where we observe that

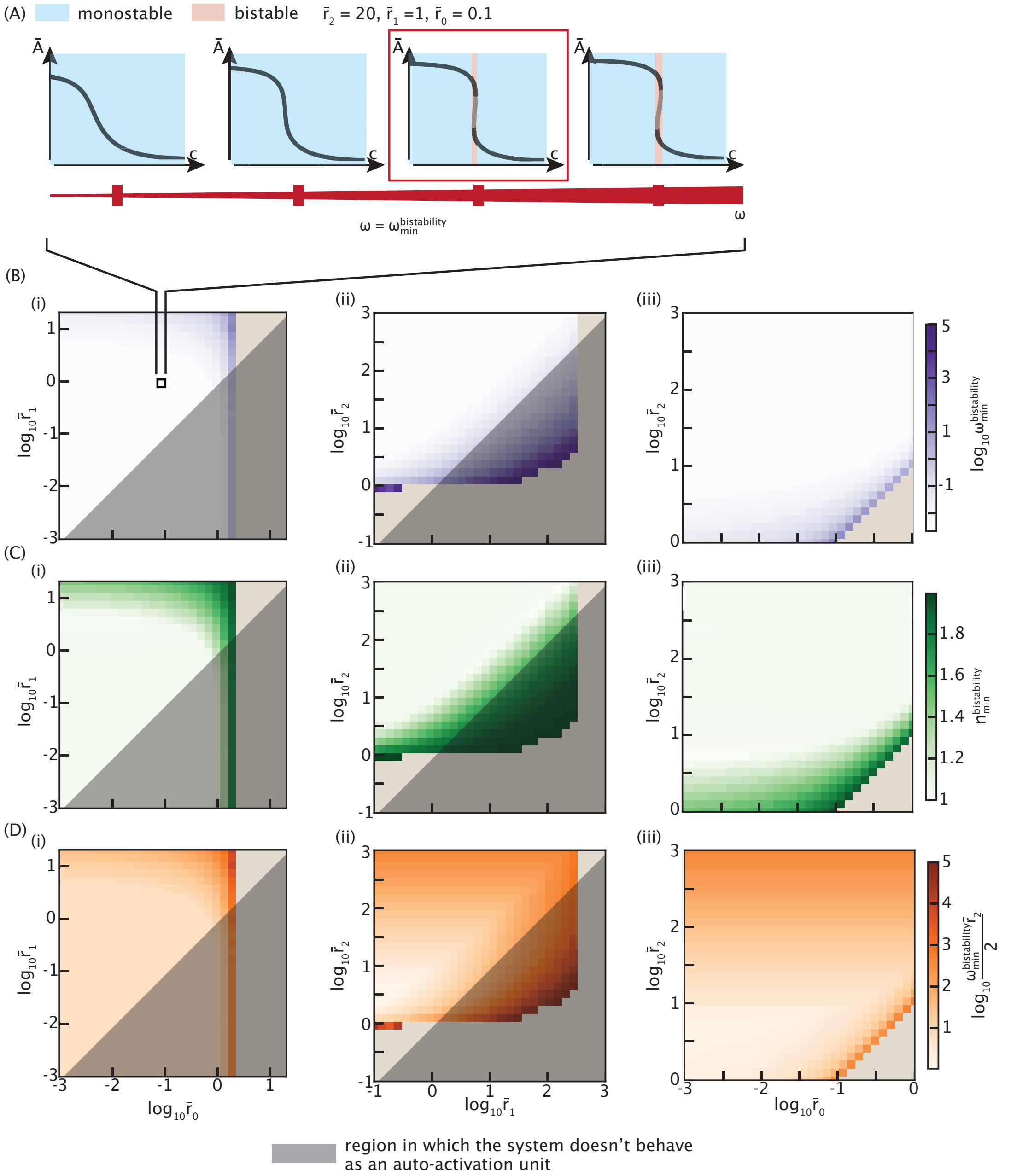

Figure 32: Parameter space exploration tracking the minimal cooperativity required for bistability over a range of effector concentrations. The cooperativity ω is sampled over the interval $\omega \in [1, 10^5]$. The condition $\bar{r}_0 \leq \bar{r}_1 \leq \bar{r}_2$ is imposed to ensure auto-activation behavior; regions where this is not satisfied are shaded in gray. (A) Illustration of the method used to determine the minimal cooperativity required for bistability. (B) Minimal cooperativity values identified using the approach in (A). (C) Corresponding effective Hill coefficient. (D) Effective cooperativity estimated from the a necessary condition for bistability $\omega\bar{r}_2/2 > 1$. In panels (B–D), subpanels (i)–(iii) show different slices of parameter space: (i) $(\bar{r}_0, \bar{r}_1) \in [10^{-5}, \bar{r}_2]^2$ with $\bar{r}_2 = 20$ fixed; (ii) $(\bar{r}_1, \bar{r}_2) \in [\bar{r}_0, 10^5]^2$ with $\bar{r}_0 = 0.1$ fixed; (iii) $(\bar{r}_0, \bar{r}_2) \in [10^{-5}, \bar{r}_1] \times [\bar{r}_1, 10^5]$ with $\bar{r}_1 = 1$ fixed. In regions where no minimal cooperativity values for bistability are reported, the system remains monostable across the entire range of cooperativity values sampled.

the required cooperativity varies significantly across parameter space. Notably, bistability can be achieved even in the case where $\omega \leq 1$. Particularly where $\bar{r}_2$ is sufficiently large relative to $\bar{r}_1$ and $\bar{r}_0$. This includes cases where $\omega < 1$, which corresponds to anti-cooperative behavior—i.e., where the binding of the first activator decreases the likelihood of a second one binding and cases where $\omega = 1$, which corresponds to no cooperativity.

While cooperativity in the strict thermodynamic sense may not be required, the system still exhibits an ef-

fective nonlinearity sufficient to support bistability. To assess this, we compute the effective Hill coefficient of the production term, derived with a thermodynamical model, shown in Fig. 32(C). When the system is bistable, the effectively Hill coefficient always exceeds 1, consistent with theoretical expectations [83]. Furthermore, in Fig. 32(D), we evaluate an effective cooperativity based on the inequality $\omega\bar{r}_2/2 > 1$, which serves as a necessary (though not sufficient) condition for bistability. The consistency of this bound with the numerically determined $\omega_{\text{min}}^{\text{bistable}}$ highlights its predictive value.

Together, these analyses reveal that bistability is not strictly dependent on cooperative binding in the classical sense, but rather emerges from the combined effects of system parameters—particularly the balance between production rates. This underscores the importance of kinetic tuning in biological systems and the potential for bistable behavior even in regimes of weak or anti-cooperative interactions.

Appendix I: Relaxation timescale to equilibrium for the auto-activation system

We examine the relaxation timescales to steady state in the auto-activation system as a function of the initial concentration of activator A, denoted $\bar{A}_0$. To define the timescale, we employ two different methods. The first method, referred to as the *threshold approach*, involves measuring the time it takes for the system to evolve from the initial condition to a fixed fraction of its steady state. We track the time-dependent trajectory $\bar{A}(\bar{t})$ and define the relaxation timescale as the time $\bar{t}^*$ such that

$$\frac{\bar{A}(\bar{t}^*) - \bar{A}_i}{\bar{A}_f - \bar{A}_i} = \epsilon, \tag{I1}$$

where ϵ is the chosen threshold. In practice, since the system is simulated numerically over N discrete time points, the relaxation time is computed as the earliest sampled time $\bar{t}_i$ for which the normalized deviation exceeds ϵ,

$$\bar{t}^* = \min_{j\in[1,N]} \left\{ \bar{t}_j \;\middle|\; \frac{\bar{A}(\bar{t}_j) - \bar{A}_i}{\bar{A}_f - \bar{A}_i} > \epsilon \right\}. \tag{I2}$$

We report the relaxation timescale obtained using various values of ϵ in Fig. 33. All resulting curves exhibit similar behavior, indicating that the precise value of the threshold does not significantly affect the overall system dynamics.

We compare these threshold-based relaxation timescales with those obtained by fitting an exponential function to the trajectory $\bar{A}(\bar{t})$. If $\bar{A}(\bar{t})$ followed a purely exponential decay—as it does near stable fixed points—then the timescale from the exponential fit would match the threshold-based timescale for $\epsilon \approx 0.63$. As shown in Fig. 33, the curve corresponding to the exponential fit closely matches the threshold-based curve with $\epsilon = 0.63$, even when the initial condition

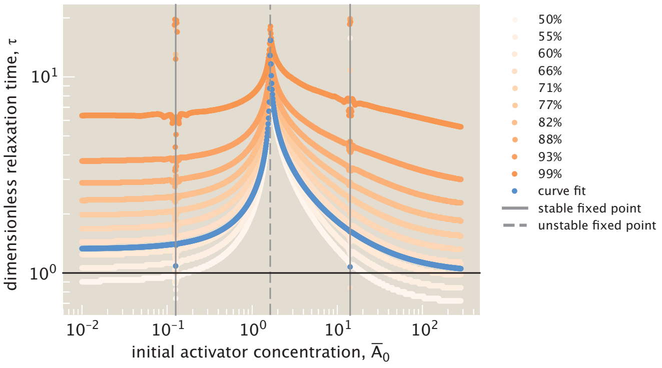

Figure 33: Relaxation timescales as a function of the initial concentration of gene A ($\bar{A}_0$). The parameters of the system are fixed at the following values: $\omega = 7.5$, $\bar{r}_0 = 0.1$, $\bar{r}_1 = 1$, $\bar{r}_2 = 20$, and $c = 2 \cdot 10^{-5}$M. Timescales are computed using two approaches: a threshold-based method (orange curves) and exponential curve fitting (blue curve). Each orange curve corresponds to a different threshold value ϵ indicated as percentages in the legend. The threshold timescale $\bar{t}^*$ is the time required for $\bar{A}(\bar{t})$ to reach a fraction ϵ of the total change from initial condition $\bar{A}_0$ to steady state $\bar{A}_\infty$. The blue curve represents the relaxation time obtained from exponential fits to $\bar{A}(\bar{t})$ trajectories. Vertical lines mark positions of stable (solid lines) and unstable (dashed line) fixed points. The horizontal black line indicates the unity timescale as a reference for comparison.

is far from the stable fixed points and the system is not strictly exponential, as illustrated in Fig. 13(A). Nevertheless, near the stable fixed points, we observe that the threshold method reports a longer timescale. This is because the initial condition is very close to that of the stable fixed point, and the system takes longer to cross the relative threshold. Therefore, we rely on the timescale obtained from the exponential fitting, as shown in Fig. 13(B).

Appendix J: Bistability regimes in the mutual repression circuit

1. A necessary condition for bistability

In the mutual repression system at steady state, we set the time derivatives to zero, leading to the equations

$$\bar{R}_1 = \frac{\bar{r}}{1 + 2p_2\bar{R}_2 + \omega_2(p_2\bar{R}_2)^2} \tag{J1}$$

and

$$\bar{R}_2 = \frac{\bar{r}}{1 + 2p_1\frac{\bar{R}_1}{\bar{K}} + \omega_1\left(p_1\frac{\bar{R}_1}{\bar{K}}\right)^2}, \tag{J2}$$

where for convenience we define $p_1 = p_{\rm act}(c_1)$ and $p_2 = p_{\rm act}(c_2)$. Substituting the expression for $\bar{R}_1$ of Eqn. J1 into Eqn. J2 for $\bar{R}_2$, and rewriting in standard polynomial form, $M(\bar{R}_2) = 0$, we obtain

$$\begin{aligned} M(\bar{R}_2) =& p_2^4\omega_2^2\bar{R}_2^5 + p_2^3\omega_2(4-\bar{r}\omega_2 p_2)\bar{R}_2^4 \\ &+ 2p_2^2(2+\omega_2(1+\bar{r}(\frac{p_1}{\bar{K}}-2p_2)))\bar{R}_2^3 \\ &+ 4p_2(1+\bar{r}(\frac{p_1}{\bar{K}}-p_2-\frac{\omega_2 p_2}{2}))\bar{R}_2^2 \\ &+ (1+\frac{2p_1\bar{r}}{\bar{K}}+\frac{\omega_1 p_1^2\bar{r}^2}{\bar{K}^2}-4p_2 r)\bar{R}_2 - \bar{r}. \end{aligned} \quad \text{(J3)}$$

To assess whether the system is monostable, we examine the number of non-negative roots of the polynomial $M(\bar{R}_2)$. If the second derivative of $M(\bar{R}_2)$ does not change sign, then the polynomial can have at most two real roots. In particular, if the polynomial is convex for all non-negative values of $\bar{R}_2$, i.e., $M''(\bar{R}_2) > 0$, then the system cannot be bistable. The second derivative of $M(\bar{R}_2)$ is given by

$$\begin{aligned} M''(\bar{R}_2) =& 20p_2^4\omega_2^2\bar{R}_2^3 + 12p_2^3\omega_2(4-\bar{r}\omega_2 p_2)\bar{R}_2^2 \\ &+ 12p_2^2\left(2+\omega_2\left(1+\bar{r}\left(\frac{p_1}{\bar{K}}-2p_2\right)\right)\right)\bar{R}_2 \\ &+ 8p_2\left(1+\bar{r}\left(\frac{p_1}{\bar{K}}-p_2-\frac{\omega_2 p_2}{2}\right)\right). \end{aligned} \quad \text{(J4)}$$

To guarantee that $M''(\bar{R}_2) > 0$ for all non-negative values of $\bar{R}_2$, we require that all coefficients in the polynomial expression of $M''(\bar{R}_2)$ remain strictly positive. This condition translates into three distinct inequalities. First, the positivity of the quadratic term, requires that

$$4 - \bar{r}\omega_2 p_2 > 0. \quad \text{(J5)}$$

Next, positivity of the linear term imposes the constraint

$$2+\omega_2\left(1+\bar{r}\left(\frac{p_1}{\bar{K}}-2p_2\right)\right) > 0. \quad \text{(J6)}$$

Finally, the positivity of the constant term yields

$$1+\bar{r}\left(\frac{p_1}{\bar{K}}-p_2-\frac{\omega_2 p_2}{2}\right) > 0. \quad \text{(J7)}$$

These conditions can be equivalently rewritten in terms of upper bounds on p_2 and combinations of p_1 and p_2, yielding

$$p_2 < \frac{4}{\bar{r}\omega_2}, \quad \text{(J8)}$$

$$2p_2 - \frac{p_1}{\bar{K}} < \frac{1}{\bar{r}}\left(\frac{2}{\omega_2}+1\right), \quad \text{(J9)}$$

$$\left(1+\frac{\omega_2}{2}\right)p_2 - \frac{p_1}{\bar{K}} < \frac{1}{\bar{r}}. \quad \text{(J10)}$$

To ensure that the system remains monostable for all values of effector concentrations c_1 and c_2, we require that these inequalities hold for the maximum possible values for the different functions of c_1 and c_2. Thus, we obtain the sufficient conditions

$$p_{\max} < \frac{4}{\bar{r}\omega_2}, \quad \text{(J11)}$$

$$2p_{\max} - \frac{p_{\min}}{\bar{K}} < \frac{1}{\bar{r}}\left(\frac{2}{\omega_2}+1\right), \quad \text{(J12)}$$

$$\left(1+\frac{\omega_2}{2}\right)p_{\max} - \frac{p_{\min}}{\bar{K}} < \frac{1}{\bar{r}}, \quad \text{(J13)}$$

Finally, in the special case where $\bar{K} = 1$, a sufficient conditions under which the system remains monostable for all effector concentrations simplify, using Mathematica, to

$$\bar{r} < \frac{1}{p_{\max} - p_{\min} + \omega_2 p_{\max}/2}. \quad \text{(J14)}$$

Taking the contrapositive, we obtain a necessary condition for the system to exhibit bistability at some value of the effector concentration

$$\bar{r} > \frac{1}{p_{\max} - p_{\min} + \omega_2 p_{\max}/2} \quad \text{(J15)}$$

The bound stated in Eqn. J15 depends on both ω_2 and $\bar{r}$, again, similarly to the auto-activation system, acting together to determine wether bistability can be accessed or not.

For $\bar{K} = 1$ the two cooperativities play a symmetric role. Therefore necessary conditions for bistability are

$$\bar{r} > \frac{1}{p_{\max} - p_{\min} + \omega_2 p_{\max}/2} \quad \text{(J16)}$$

and

$$\bar{r} > \frac{1}{p_{\max} - p_{\min} + \omega_1 p_{\max}/2}. \quad \text{(J17)}$$

From Eqns. J17 and J16 a necessary condition for bistability for $K = 1$ is that

$$\bar{r} > \max(\frac{1}{p_{\max} - p_{\min} + \omega_2 p_{\max}/2}, \frac{1}{p_{\max} - p_{\min} + \omega_1 p_{\max}/2}). \quad \text{(J18)}$$

which simplifies to

$$\bar{r} > \frac{1}{p_{\max} - p_{\min} + \min(\omega_2, \omega_1) p_{\max}/2}. \quad \text{(J19)}$$

2. Effective Hill coefficient of the production terms

In the case of auto-activation, analyzing the effective Hill coefficient provided insight into how bistability can arise even in non-cooperative systems—$\omega > 1$ is not a necessary condition for bistability, but from our numerical sweeps displayed in Fig. 32, $n_{\rm eff} > 1$ is. Motivated by this, we now examine whether a similar criterion might help explain the restriction of bistability to specific zones of parameter space in mutual repression circuits.

We defined (Eqn. H15, Eqn. H17) and derived an analytical formula (Eqn. H14, Eqn. H13) for the effective Hill coefficient for a general production term

$$w(x) = \frac{\bar{r}_0 + 2\bar{r}_1 p_{\rm act} x + \omega \bar{r}_2 p_{\rm act} x^2}{1 + 2p_{\rm act} x + \omega p_{\rm act} x^2}, \tag{J20}$$

in Appendix H 1. In the mutual repression system, the production terms of interest are the production of R_1 driven by promoter 1 and regulated by R_2

$$f_1(\bar{R}_2) = \frac{\bar{r}}{1 + 2(p_{\rm act}(c_2)\bar{R}_2) + \omega_2 (p_{\rm act}(c_2)\bar{R}_2)^2} \tag{J21}$$

and the production of R_2 driven by promoter 2 and regulated by R_1

$$f_2(\bar{R}_1) = \frac{\bar{r}}{1 + 2(p_{\rm act}(c_1)\frac{\bar{R}_1}{\bar{K}}) + \omega_1 (p_{\rm act}(c_1)\frac{\bar{R}_1}{\bar{K}})^2}. \tag{J22}$$

With $\bar{r}_0 = \bar{r}$, $\bar{r}_1 = 0$, and $\bar{r}_2 = 0$, $p_{\rm act} \equiv p_{\rm act}(c_2)$ for $f_1(\bar{R}_2)$ and $p_{\rm act} \equiv p_{\rm act}(c_1)/K$ for $f_2(\bar{R}_1)$; we see that those two production terms fall in to the more general from $w(x)$ of Eqn. J20. We can therefore apply the reasoning and algebra derived in Appendix H 1. The corresponding effective Hill coefficients are given by

$$n_1 = 2 - \frac{\sqrt{1+4\omega_2} - 1}{2\omega_2}, \tag{J23}$$

for the production term $f_1(\bar{R}_2)$ and

$$n_2 = 2 - \frac{\sqrt{1+4\omega_1} - 1}{2\omega_1}, \tag{J24}$$

for the production term $f_2(\bar{R}_1)$. We see that the two expressions of the effective Hill coefficients have the same functional form. Therefore showing that $n_1 > 1$ for all $\omega_2 > 0$, implies that $n_2 > 1$ for all $\omega_1 > 0$. We therefore establish that $n_1 > 1$ for all $\omega_2 > 0$, and $n_2 > 1$ for all $\omega_1 > 0$ then follows. This amounts to showing

$$2\omega_2 \; > \; \sqrt{1+4\omega_2} - 1. \tag{J25}$$

Adding 1 to each side and squaring both sides gives

$$\big(2\omega_2 + 1\big)^2 \; > \; 1 + 4\omega_2. \tag{J26}$$

Expanding the square and canceling identical terms leaves

$$4\omega_2^2 \; > \; 0, \tag{J27}$$

which is true for every $\omega_2 > 0$.

We therefore showed that the effective Hill coefficients of the production terms are always greater than one in this system. As a result, while this observation is consistent with conventional expectations [83], it provides little discriminatory power for identifying regions of bistability, since the condition is satisfied across parameter space.

3. Effect of cooperativity on the bistability region

Fig. 34(A)(i) shows how the geometry of the bistable region evolves as the cooperativity of repressor R_2 (ω_2) is varied, while $\omega_1 = 7.5$, $\bar{K} = 1$, and $\bar{r} = 2$ are held fixed. This corresponds to a symmetric case where both repressors bind their respective promoters with equal affinity. At low ω_2, the system is monostable for all inducer concentrations, which is consistent with the known requirement for a minimal degree of nonlinearity to enable bistability. As ω_2 increases beyond a threshold, bistability emerges, but initially in a constrained region where the tunability is mostly limited by c_1. When ω_2 lies approximately between 2 and 5, a nonzero concentration of c_1 is required to inactivate a portion of the repressors R_1, thereby reducing their ability to bind DNA efficiently. In this regime, we are still in a setting where $\omega_1 > \omega_2$, meaning that R_1 binds more strongly to the DNA than R_2—as they have equal binding constants, the difference in binding arises solely from the cooperativity parameters. To support two distinct expression states—one with high R_1 and low R_2, and another with the reverse—the binding strength of R_1 must be reduced. This enables a more balanced competition between the two repressors, making it possible for both stable states to coexist. When ω_2 becomes large (e.g., $\omega_2 \gtrsim 20$), the situation reverses: the bistable region in the (c_1, c_2) phase space becomes constrained along the c_2 axis, as higher concentrations of inducer are required to counteract the strong DNA binding of R_2.

For intermediate values of cooperativity, approximately between 5 and 20, the (c_1, c_2) phase space is less constrained. In this regime, the concentrations of c_1 and c_2 need to be small enough to maintain repression by R_1 and R_2. If either inducer concentration becomes too high, the system is not repressed anymore and only has a unique steady state with high concentrations of both repressors.

While increasing ω_2 initially expands the bistable region and enhances its robustness, we find that beyond a certain threshold, further increases in cooperativity begin to shrink the bistable domain. This reflects a general principle also observed for the parameter $\bar{K}$: pushing the system too far in one direction strongly constrains bistability. In the case of ω_2, overly strong cooperativity amplifies the binding of R_2 and therefore the repression of R_1, so the system commits to one state, thereby reducing the range of inducer concentrations for which multiple steady states coexist.

Fig. 34(A)(ii) explores the impact of tuning ω_2 in an asymmetric setting, where $\bar{K} = 0.7$, $\omega_1 = 7.5$, and $r = 2$ are fixed. In this case, repressor R_1 binds more tightly to its promoter than R_2 does, breaking the symmetry observed in Fig. 34(A)(i). At low values of ω_2, the system is monostable, consistent with insufficient nonlinearity to support multiple steady states. As ω_1 increases, bistability appears, but the geometry of the bistable region is notably skewed. Compared to Fig. 34(A)(i), it is

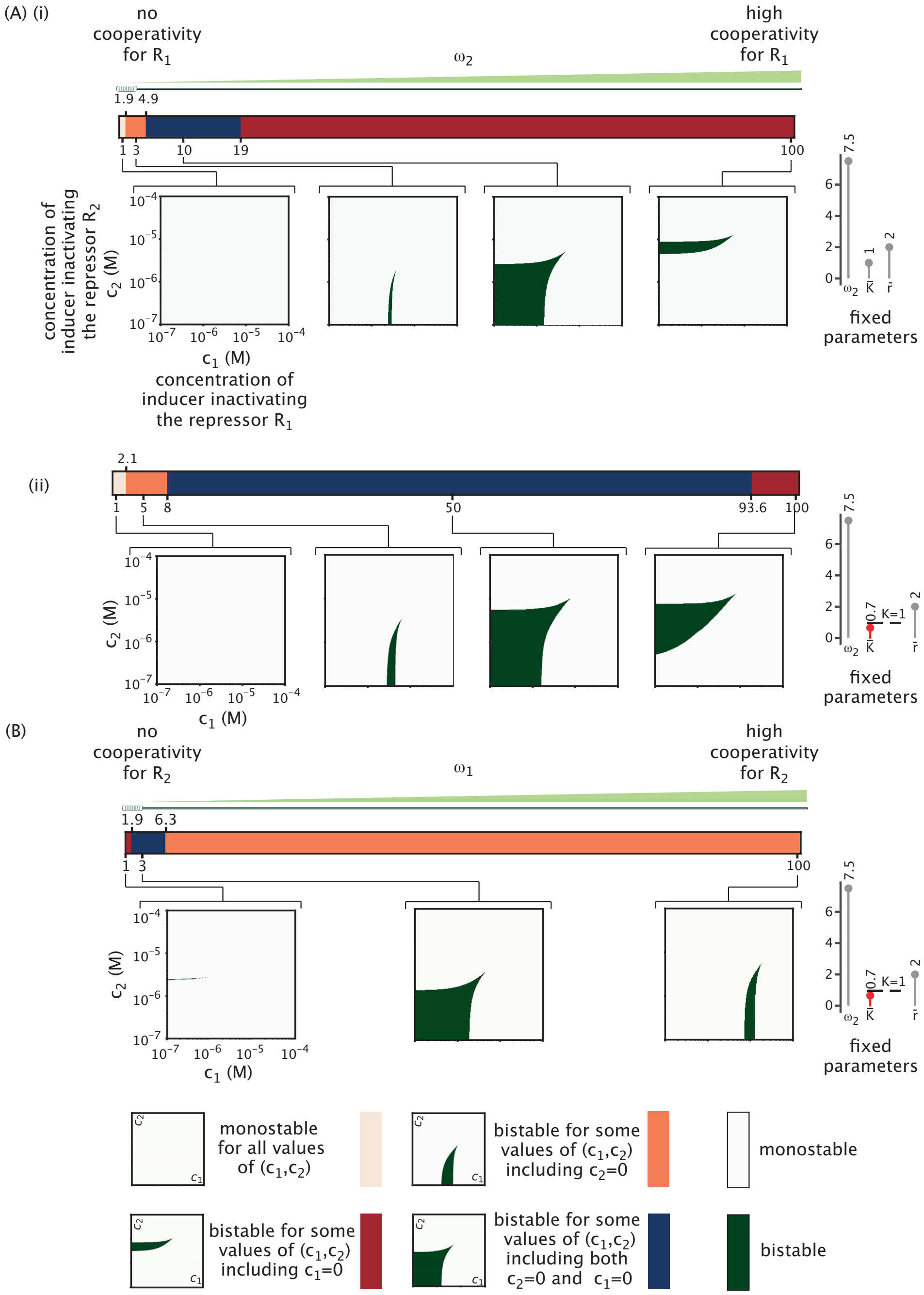

Figure 34: Bistability regimes in mutual repression as a function of cooperativity. Colored regions denote distinct bistable phase space geometries, defined by whether bistability occurs at very small c_1 ($c_1 = 10^{-7}$ M), very small c_2 ($c_2 = 10^{-7}$ M), both, or neither. (A) Evolution of the geometry of the bistability phase space, sweeping on inducer concentrations (c_1, c_2), for fixed $\omega_1 = 7.5$ and $\bar{r} = 2$ and respectively $\bar{K} = 1$ and $\bar{K} = 0.7$ for (i) and (ii), when the parameter ω_2 is varied. (B) Evolution of the geometry of the bistability phase space, sweeping on inducer concentrations (c_1, c_2), for fixed $\omega_2 = 7.5$, $\bar{r} = 2$ and $\bar{K} = 0.7$, when the parameter ω_1 is varied.

interesting to note that the bistability region at higher cooperativity is larger than in the symmetric case for the range of inducer concentrations considered. Finally in Fig. 34(B), we tune the cooperativity ω_1 instead of ω_2 as was done in the previous panel. Therefore the roles of c_1 and c_2 are mirrored.

Appendix K: Separatrix for mutual repression

We recall the differential equations governing the mutual repression system,

$$\frac{d\bar{R}_1}{d\bar{t}} = -\bar{R}_1 + \bar{r}\frac{1}{1 + 2p_{\mathrm{act}}(c_2)\bar{R}_2 + \omega_2\left[p_{\mathrm{act}}(c_2)\bar{R}_2\right]^2} = F(\bar{R}_1, \bar{R}_2), \quad \text{(K1)}$$

$$\frac{d\bar{R}_2}{d\bar{t}} = -\bar{R}_2 + \bar{r}\frac{1}{1 + 2p_{\text{act}}(c_1)\frac{\bar{R}_1}{\bar{K}} + \omega_1\left[p_{\text{act}}(c_1)\frac{\bar{R}_1}{\bar{K}}\right]^2}$$
$$= G(\bar{R}_1, \bar{R}_2). \quad \text{(K2)}$$

The separatrix is defined as the curve $\bar{R}_2(\bar{R}_1)$ that satisfies the differential equation

$$\frac{d\bar{R}_2}{d\bar{R}_1} = \frac{G(\bar{R}_1, \bar{R}_2)}{F(\bar{R}_1, \bar{R}_2)}, \quad \text{(K3)}$$

which tracks the trajectory along which the system transitions between the basins of attraction of the two stable steady states.

Appendix L: Coherent feed-forward loop response to a step function signal

1. Analytical solution for the output $\bar{Z}(\bar{t})$

We now rewrite the dynamical equations of the coherent feed-forward loop. In particular, we introduce a simplifying notation for the activation terms for gene products Y and Z. The reason such a definition is useful is that these terms are independent of Y and Z themselves and depend only upon X itself and the concentration of inducer. To that end, we write the dynamical equations for Y and Z as

$$\frac{d\bar{Y}}{d\bar{t}} = -\bar{Y} + f_Y(t) \quad \text{(L1)}$$
$$\frac{d\bar{Z}}{d\bar{t}} = -\bar{Z} + f_Z(t), \quad \text{(L2)}$$

with the simplifying notation

$$f_Y(\bar{t}) = \frac{\bar{r}_{0Y} + \bar{r}_{1Y}\, p^X_{\text{act}}(c_X(\bar{t}))\bar{X}}{1 + p^X_{\text{act}}(c_X(\bar{t}))\bar{X}}, \quad \text{(L3)}$$

and

$$f_Z(\bar{t}) = \frac{\bar{r}_{0Z} + \bar{r}_{1Z}(\mathcal{X}(\bar{t}) + \mathcal{Y}(\bar{t})) + \omega\bar{r}_{2Z}\mathcal{X}(\bar{t})\mathcal{Y}(\bar{t})}{1 + \mathcal{X}(\bar{t}) + \mathcal{Y}(\bar{t}) + \omega\mathcal{X}(\bar{t})\mathcal{Y}(\bar{t})}. \quad \text{(L4)}$$

We recall that the bar indicates quantities where time is measured in units of $1/\gamma$, and where concentration and dissociation constants are measured in units of K_{XY}. The rates are then in units of γK_{XY}. The notations $\mathcal{X}$ and $\mathcal{Y}$ and are defined as $\mathcal{X}(\bar{t}) = p^X_{\text{act}}(c_X(\bar{t}))\bar{X}/\bar{K}_{XZ}$ and $\mathcal{Y}(\bar{t}) = p^Y_{\text{act}}(c_Y)\bar{Y}(\bar{t})/\bar{K}_{YZ}$. We study the response of the coherent feed-forward loop to a step function in effector concentration acting on X, namely,

$$c_X(\bar{t}) = \begin{cases} c^i_X & \text{if} \quad \bar{t} \leq 0, \\ c^f_X & \text{if} \quad \bar{t} > 0. \end{cases} \quad \text{(L5)}$$

The step in the active concentration of X and the rescaled concentration $\mathcal{X}$ are themselves subject to a step and can be written as

$$\mathcal{X}(\bar{t}) = \begin{cases} \mathcal{X}_{\text{i}} = \dfrac{p^X_{\text{act}}(c^i_X)\bar{X}}{\bar{K}_{XZ}} & \text{if} \quad \bar{t} \leq 0, \\ \mathcal{X}_{\text{f}} = \dfrac{p^X_{\text{act}}(c^f_X)\bar{X}}{\bar{K}_{XZ}} & \text{if} \quad \bar{t} > 0. \end{cases} \quad \text{(L6)}$$

The concentration of effector acting on Y, $c_Y(\bar{t})$ is taken to be constant $c_Y(\bar{t}) = c^0_Y$. Our goal here is to solve for the feed-forward dynamics analytically and obtain insights into the system on the basis of such a solution. Such a solution is possible because Eqns. 25 and 26 have a simple form, the time derivative of a variable equals the negative of itself plus a function of time,

$$\frac{dG(t)}{dt} = -G(t) + f(t). \quad \text{(L7)}$$

Such equations can be solved in their most general form as

$$G(t) = e^{-t}\Big(G(0) + \int_0^t e^{t'} f(t')dt'\Big). \quad \text{(L8)}$$

In the cases of interest here, $G(t)$ is either $\bar{Y}(\bar{t})$ or $\bar{Z}(\bar{t})$, and $f(t)$ correspondingly is either $f_Y(\bar{t})$ or $f_Z(\bar{t})$, the activation terms for Y and Z, respectively, in Eqns. L2 and L1.

We first solve Eqn. L1, because its dynamics is not coupled to $\bar{Z}(\bar{t})$. Notice that for $\bar{t} > 0$, $f_Y(\bar{t})$ is constant. Referring to Eqn. L8, we see that $\bar{Y}$ evolves from initial to final state purely exponentially according to the time evolution

$$\bar{Y}(\bar{t}) = -\Delta\bar{Y}e^{-\bar{t}} + \bar{Y}_f. \quad \text{(L9)}$$

Here $\Delta\bar{Y} = \bar{Y}_f - \bar{Y}_i$ is the difference between the final and initial concentration of $\bar{Y}$. We write the explicit expression of those initial and final steady states hereafter,

$$\bar{Y}_i = \frac{\bar{r}_{0Y} + \bar{r}_{1Y}\, p^X_{\text{act}}(c^i_X)\bar{X}}{1 + p^X_{\text{act}}(c^i_X)\bar{X}} \quad \text{(L10)}$$
$$\bar{Y}_f = \frac{\bar{r}_{0Y} + \bar{r}_{1Y}\, p^X_{\text{act}}(c^f_X)\bar{X}}{1 + p^X_{\text{act}}(c^f_X)\bar{X}}. \quad \text{(L11)}$$

As $p^Y_{\text{act}}(c_Y(\bar{t})) = p^Y_{\text{act}}(c^0_Y)$ is constant, $\bar{Y} \to \mathcal{Y}$ is a proportional mapping. Therefore, like $\bar{Y}(t)$, $\mathcal{Y}(\bar{t})$ also evolves exponentially in time by the similar form

$$\mathcal{Y}(\bar{t}) = -\Delta\mathcal{Y}e^{-\bar{t}} + \mathcal{Y}_f, \quad \text{(L12)}$$

with $\mathcal{Y}_{f/i} = p^Y_{\text{act}}(c^0_Y)\bar{Y}_{f/i}/\bar{K}_{YZ}$ and $\Delta\mathcal{Y} = \mathcal{Y}_f - \mathcal{Y}_i$. Given this expression for $\mathcal{Y}(\bar{t})$, we now know the full time dependence of $f_Z(\bar{t})$ in Eqn. L2. Next, we can solve for the dynamics of $\bar{Z}$ by substituting $f_Z(\bar{t})$ into the general

solution Eqn. L8. By evaluating the integral, we find that

$$\begin{aligned}\bar{Z}(\bar{t}) &= \bar{Z}_i e^{-\bar{t}} + \Big(e^{-\bar{t}}\int_0^{\bar{t}} e^{t'} f_Z(t')dt'\Big) \\ &= \bar{Z}_i e^{-\bar{t}} + \Big(\bar{Z}_f(1-e^{-\bar{t}}) + \Theta(\bar{t})\Big) \\ &= \bar{Z}_{\text{simple}}(\bar{t}) + \Theta(\bar{t}),\end{aligned} \tag{L13}$$

with

$$\begin{aligned}\Theta(\bar{t}) &= -\frac{\Phi\Delta\mathcal{Y}}{S^2}e^{-\bar{t}}\log\left(\frac{Se^{\bar{t}} - \Delta\mathcal{Y}(1+\omega\mathcal{X}_f)}{S-\Delta\mathcal{Y}(1+\omega\mathcal{X}_f)}\right), \\ \Phi &= \omega\mathcal{X}_f^2(\bar{r}_{2Z}-\bar{r}_{1Z}) + \omega\mathcal{X}_f(\bar{r}_{2Z}-\bar{r}_{0Z}) \\ &\quad + (\bar{r}_{1Z}-\bar{r}_{0Z}), \\ S &= 1+\mathcal{X}_f+\mathcal{Y}_f+\omega\mathcal{X}_f\mathcal{Y}_f,\end{aligned} \tag{L14}$$

as shown in the main text. The solution of $\bar{Z}(\bar{t})$ cleanly splits into two parts. The first two terms describe the exponential behavior one expects from simple regulation

$$\bar{Z}_{\text{simple}}(\bar{t}) = \bar{Z}_i e^{-\bar{t}} + \bar{Z}_f(1-e^{-\bar{t}}) \tag{L15}$$

with $\bar{Z}_i$ and $\bar{Z}_f$, respectively the initial and final steady state concentration of the output $\bar{Z}$. Their explicit expression is given by

$$\bar{Z}_i = \frac{\bar{r}_{0Z} + \bar{r}_{1Z}(\mathcal{X}_i+\mathcal{Y}_i) + \omega\bar{r}_{2Z}\mathcal{X}_i\mathcal{Y}_i}{1+\mathcal{X}_i+\mathcal{Y}_i+\omega\mathcal{X}_i\mathcal{Y}_i} \tag{L16}$$

$$\bar{Z}_f = \frac{\bar{r}_{0Z} + \bar{r}_{1Z}(\mathcal{X}_f+\mathcal{Y}_f) + \omega\bar{r}_{2Z}\mathcal{X}_f\mathcal{Y}_f}{1+\mathcal{X}_f+\mathcal{Y}_f+\omega\mathcal{X}_f\mathcal{Y}_f}. \tag{L17}$$

We see that $\Theta(\bar{t})$ accounts for the difference between the feed-forward trajectory $\bar{Z}$ and the simple regulation trajectory $\bar{Z}_{\text{simple}}$. As a sanity check, we see $\Theta(\bar{t}) = 0$ when $\bar{t} = 0$ and $\bar{t} \to \infty$, confirming that the feed-forward loop and the simple regulation trajectory have the same initial and final state, as expected.

2. Derivation and sign of the average delay $\langle\Delta\bar{t}\rangle$

From the analytical expression of $\bar{Z}(\bar{t})$, we can then also derive the average time delay from the offset $\Theta(\bar{t})$

$$\langle\Delta\bar{t}\rangle = \frac{1}{\bar{Z}_f - \bar{Z}_i}\int_0^\infty \Theta(\bar{t})d\bar{t}. \tag{L18}$$

With Eqn. L14, we can analytically evaluate the integral and obtain the following expression for the average delay

$$\langle\Delta\bar{t}\rangle = \frac{\Phi(\bar{Z}_f-\bar{Z}_i)^{-1}}{S(1+\omega\mathcal{X}_f)}\log\Big(\frac{1+\mathcal{X}_f+\mathcal{Y}_i+\omega\mathcal{X}_f\mathcal{Y}_i}{S}\Big). \tag{L19}$$

As a reminder, $\langle\Delta\bar{t}\rangle$ signifies the average time difference between the feed-forward loop response and the simple regulation response. The sign of $\langle\Delta\bar{t}\rangle$ indicates whether the feed-forward loop delays ($\langle\Delta\bar{t}\rangle < 0$) or accelerates ($\langle\Delta\bar{t}\rangle > 0$). From Eqn. L19, we can analytically determine whether the feed-forward loop delays or accelerates by treating the contribution from each component. To begin with, we have $S > 0$ and $1+\omega\mathcal{X}_f > 0$ as concentrations are strictly non-negative. For the coherent feed-forward loop, we have $\Phi \geq 0$ as $\bar{r}_{2Z} \geq \bar{r}_{1Z} \geq \bar{r}_{0Z}$ since both X and Y activate Z. The term $(\bar{Z}_f - \bar{Z}_i)$ depends on the direction of the step. For an ON step, $(\bar{Z}_f - \bar{Z}_i) > 0$ and for an OFF step, $(\bar{Z}_f - \bar{Z}_i) < 0$. Finally, the logarithm also depends on the direction of the step. For an ON step, we have

$$1+\mathcal{X}_f+\mathcal{Y}_i+\omega\mathcal{X}_f\mathcal{Y}_i \leq 1+\mathcal{X}_f+\mathcal{Y}_f+\omega\mathcal{X}_f\mathcal{Y}_f = S \tag{L20}$$

$$\implies \log\Big(\frac{1+\mathcal{X}_f+\mathcal{Y}_i+\omega\mathcal{X}_f\mathcal{Y}_i}{S}\Big) \leq 0, \tag{L21}$$

since $\mathcal{X}_f \geq \mathcal{X}_i$ and $\mathcal{Y}_f \geq \mathcal{Y}_i$. Combined with the effect of other terms, we find $\langle\Delta\bar{t}\rangle \leq 0$ for an ON step. Similarly, for the OFF step, we have

$$1+\mathcal{X}_f+\mathcal{Y}_i+\omega\mathcal{X}_f\mathcal{Y}_i \geq 1+\mathcal{X}_f+\mathcal{Y}_f+\omega\mathcal{X}_f\mathcal{Y}_f = S \tag{L22}$$

$$\implies \log\Big(\frac{1+\mathcal{X}_f+\mathcal{Y}_i+\omega\mathcal{X}_f\mathcal{Y}_i}{S}\Big) \geq 0, \tag{L23}$$

and we thus find that $\langle\Delta\bar{t}\rangle \leq 0$ for the OFF step as well.

We have shown that the average time difference $\langle\Delta\bar{t}\rangle$ is negative for both the ON and OFF steps. To complete the analysis, we will further demonstrate that the time difference $\Delta\bar{t}(\bar{Z})$, defined in Section IV A, has the same sign for any concentration $\bar{Z}$. This amounts to saying that the feed-forward trajectory and the simple regulation trajectory never cross each other. We can show this by proving that $\Theta(\bar{t})$ has the same sign for any $\bar{t} > 0$. In Eqn. L14, we observe that the time dependence in $\Theta(\bar{t})$ appears in $e^{-\bar{t}}$ and in $Se^{\bar{t}}$ inside the logarithm. $e^{-\bar{t}} > 0$ for any $\bar{t}$, thus only the logarithm term might change its sign as time evolves. However, we observe that

$$\begin{aligned}S - \Delta\mathcal{Y}(1+\omega\mathcal{X}) = 1 &+ \mathcal{X}_f + \mathcal{Y}_i \\ &+\omega\mathcal{X}_f\mathcal{Y}_i > 0.\end{aligned} \tag{L24}$$

In other words, $S > \Delta\mathcal{Y}(1+\omega\mathcal{X})$. Since $e^{\bar{t}} > 1$ for any $\bar{t} > 0$, it is always true that $Se^{\bar{t}} > \Delta\mathcal{Y}(1+\omega\mathcal{X})$. Therefore, the sign of the logarithm does not change with time. Thus, we have proven that $\Delta\bar{t}(\bar{Z})$ has the same sign as $\langle\Delta\bar{t}\rangle$ for any concentration $\bar{Z}$.

3. Logic gate analysis in coherent feed-forward loop

In this appendix section, we will prove that for the XOR gate in the coherent feed-forward loop, the OFF

step delay is always greater than the ON step delay. In the XOR limit, $\omega = 0$. Eqn. 38 simplifies to

$$\langle \Delta \bar{t} \rangle = \frac{1}{(\bar{Z}_f - \bar{Z}_i)} \frac{\bar{r}_{1Z} - \bar{r}_{0Z}}{1 + \mathcal{X}_f + \mathcal{Y}_f} \log \Big(\frac{1 + \mathcal{X}_f + \mathcal{Y}_i}{1 + \mathcal{X}_f + \mathcal{Y}_f} \Big). \tag{L25}$$

The time delay $\langle \Delta \bar{t} \rangle$ is different for ON and OFF steps because $\mathcal{X}$ and $\mathcal{Y}$ values at $\bar{t} = 0$ and $\bar{t} = \infty$ are different for ON and OFF steps. Specifically, for ON step, $\mathcal{X}_f = \mathcal{X}_{\max}$ and $\mathcal{Y}_f = \mathcal{Y}_{\max}$; while for OFF step $\mathcal{X}_f = \mathcal{X}_{\min}$ and $\mathcal{Y}_f = \mathcal{Y}_{\min}$. Let's now compare the time delay $\langle \Delta \bar{t} \rangle$ for ON and OFF steps by taking their ratio

$$\begin{aligned} \left| \frac{\langle \Delta \bar{t} \rangle_{\text{OFF}}}{\langle \Delta \bar{t} \rangle_{\text{ON}}} \right| &= \left| \frac{1 + \mathcal{X}_{\max} + \mathcal{Y}_{\max}}{1 + \mathcal{X}_{\min} + \mathcal{Y}_{\min}} \frac{\log \Big(\frac{1 + \mathcal{X}_{\min} + \mathcal{Y}_{\max}}{1 + \mathcal{X}_{\min} + \mathcal{Y}_{\min}} \Big)}{\log \Big(\frac{1 + \mathcal{X}_{\max} + \mathcal{Y}_{\min}}{1 + \mathcal{X}_{\max} + \mathcal{Y}_{\max}} \Big)} \right| \\ &= \frac{1 + \mathcal{X}_{\max} + \mathcal{Y}_{\max}}{1 + \mathcal{X}_{\min} + \mathcal{Y}_{\min}} \frac{\log \Big(\frac{1 + \mathcal{X}_{\min} + \mathcal{Y}_{\max}}{1 + \mathcal{X}_{\min} + \mathcal{Y}_{\min}} \Big)}{\log \Big(\frac{1 + \mathcal{X}_{\max} + \mathcal{Y}_{\max}}{1 + \mathcal{X}_{\max} + \mathcal{Y}_{\min}} \Big)}. \end{aligned} \tag{L26}$$

Note that $1 + \mathcal{X}_{\max} + \mathcal{Y}_{\max} \geq 1 + \mathcal{X}_{\min} + \mathcal{Y}_{\min}$, and that

$$\frac{1 + \mathcal{X}_{\min} + \mathcal{Y}_{\max}}{1 + \mathcal{X}_{\min} + \mathcal{Y}_{\min}} \geq \frac{1 + \mathcal{X}_{\max} + \mathcal{Y}_{\max}}{1 + \mathcal{X}_{\max} + \mathcal{Y}_{\min}}, \tag{L27}$$

because for any fraction a/b where $a \geq b > 0$, $a/b \geq (a+c)/(b+c)$ for any $c \geq 0$. Here, $a = 1 + \mathcal{X}_{\min} + \mathcal{Y}_{\max}$, $b = 1 + \mathcal{X}_{\min} + \mathcal{Y}_{\min}$, and $c = \mathcal{X}_{\max} - \mathcal{X}_{\min}$. As logarithm is an increasing function, this means

$$\log \frac{1 + \mathcal{X}_{\min} + \mathcal{Y}_{\max}}{1 + \mathcal{X}_{\min} + \mathcal{Y}_{\min}} \geq \log \frac{1 + \mathcal{X}_{\max} + \mathcal{Y}_{\max}}{1 + \mathcal{X}_{\max} + \mathcal{Y}_{\min}}. \tag{L28}$$

Therefore, the ratio $|\langle \Delta \bar{t} \rangle_{\text{OFF}} / \langle \Delta \bar{t} \rangle_{\text{ON}}| \geq 1$. We have demonstrated that for the XOR gate, the OFF step delay is larger than the ON step delay. We cannot say much analytically about the magnitude of the ratio of delay. As we see in Fig. 22(A), this ratio can range anywhere from close to 1 to a large number.

From here, we might be tempted to argue that for the AND gate, the ratio always satisfies $|\langle \Delta \bar{t} \rangle_{\text{OFF}} / \langle \Delta \bar{t} \rangle_{\text{ON}}| \leq 1$. However, Fig. 22(B)(iii) provides a counter example. The statement for AND gate is thus false.

4. Analytic solutions of other feed-forward loop networks.

In any feed-forward loop, X regulates Y and Z, and Y regulates Z. As there are three regulatory interactions, there exist $2^3 = 8$ different regulatory logics. For the dynamical equation of $\bar{Y}$, if X activates Y, then

$$\frac{d\bar{Y}}{d\bar{t}} = -\bar{Y} + \frac{\bar{r}_{0Y} + \bar{r}_{1Y} p^X_{\text{act}}(c_X) \bar{X}}{1 + p^X_{\text{act}}(c_X) \bar{X}}. \tag{L29}$$

Otherwise, if X represses Y, then

$$\frac{d\bar{Y}}{d\bar{t}} = -\bar{Y} + \frac{\bar{r}_{0Y}}{1 + p^X_{\text{act}}(c_X) \bar{X}}. \tag{L30}$$

There exist 4 different possibilities for how X and Y regulate Z. In Section IV, we showed the case where X and Y both activate Z, and the case where X activates but Y represses Z. If X represses and Y activates Z, we have

$$\frac{d\bar{Z}}{d\bar{t}} = -\bar{Z} + \frac{\bar{r}_{0Z} + \bar{r}_{1Z} \mathcal{Y}}{1 + \mathcal{X} + \mathcal{Y} + \omega \mathcal{X} \mathcal{Y}}. \tag{L31}$$

If both X and Y repress Z, then

$$\frac{d\bar{Z}}{d\bar{t}} = -\bar{Z} + \frac{\bar{r}_{0Z}}{1 + \mathcal{X} + \mathcal{Y} + \omega \mathcal{X} \mathcal{Y}}. \tag{L32}$$

Using the procedures described in the main text, we can similarly find analytic solutions describing all the other regulatory logics of the feed-forward architectures. Note that for a step function signal in c_X and c_Y,

$$\mathcal{Y}(\bar{t}) = -\Delta \mathcal{Y} e^{-\bar{t}} + \mathcal{Y}_f. \tag{L33}$$

Thus, we can compute the analytic expression of $\bar{Z}$ without worrying about how Y is regulated. We find that the solutions all have the same form, except with a different Φ for each architecture of logic. Specifically, we have

$$\begin{aligned} \Phi_{XY} &= \omega \mathcal{X}^2 (\bar{r}_{2Z} - \bar{r}_{1Z}) + \omega \mathcal{X} (\bar{r}_{2Z} - \bar{r}_{0Z}) \\ &\quad + (\bar{r}_{1Z} - \bar{r}_{0Z}) \end{aligned} \tag{L34}$$

$$\Phi_X = -(\bar{r}_{1Z} \mathcal{X} + \bar{r}_{0Z})(1 + \omega \mathcal{X}) \tag{L35}$$

$$\Phi_Y = \bar{r}_{1Z}(1 + \mathcal{X}) - \bar{r}_{0Z}(1 + \omega \mathcal{X}) \tag{L36}$$

$$\Phi_0 = -\bar{r}_{0Z}(1 + \omega \mathcal{X}), \tag{L37}$$

where the subscript on Φ indicates which TF activates Z. $\mathcal{X} = \mathcal{X}_f$ is the value of $\mathcal{X}$ after the step function jump. Interestingly, we can write these expressions as

$$\Phi_{XY} = \omega \mathcal{X}(1 + \mathcal{X}) \bar{r}_{2Z} + \Phi_X + \Phi_Y - \Phi_0 \tag{L38}$$

$$\Phi_X = -\bar{r}_{1Z} \mathcal{X}(1 + \omega \mathcal{X}) + \Phi_0 \tag{L39}$$

$$\Phi_Y = \bar{r}_{1Z}(1 + \mathcal{X}) + \Phi_0 \tag{L40}$$

$$\Phi_0 = -\bar{r}_{0Z}(1 + \omega \mathcal{X}). \tag{L41}$$

A way to interpret this is that Φ gets a new term associated with a weight of a state when that state changes from no expression to expression.

Appendix M: Functionality condition comparison

In Section IV B, we mentioned that besides the time delay, another helpful criterion for a functional feed-forward loop is the existence of a large difference between

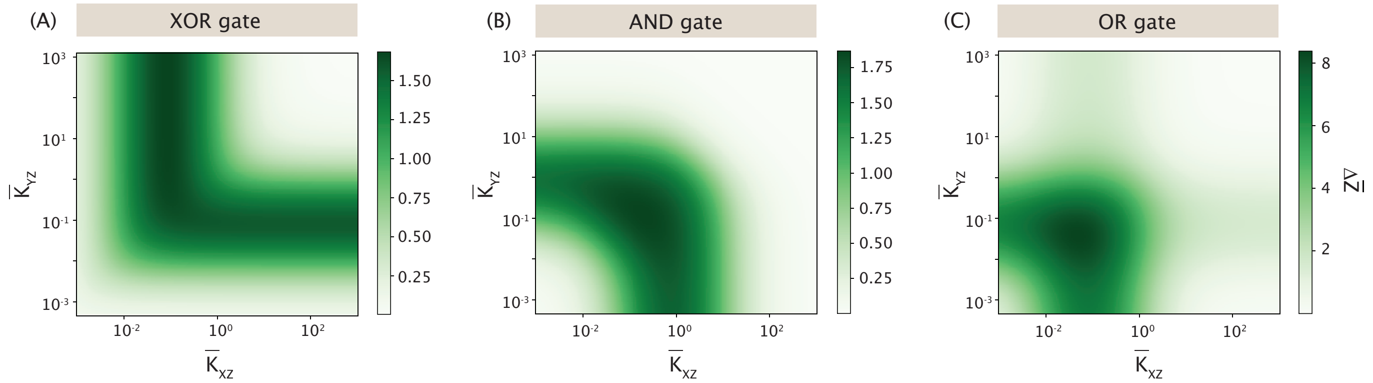

Figure 35: Systematic sweep to find region of large $\Delta\bar{Z} = \bar{Z}_{\max} - \bar{Z}_{\min}$. For every logic gate, we sweep across $(\bar{K}_{XZ}, \bar{K}_{YZ}) \in [10^{-3}, 10^3] \times [10^{-3}, 10^3]$ and inspect the region of large relative $\Delta\bar{Z}$. (A) XOR gate. (B) AND gate. (C) OR gate. The XOR gate parameters are $\bar{r}_{0Y} = \bar{r}_{0Z} = 0$, $\bar{r}_{1Y} = \bar{r}_{1Z} = 2$, and $\omega = 0$. The AND gate parameters are $\bar{r}_{0Y} = \bar{r}_{0Z} = \bar{r}_{1Z} = 0$, $\bar{r}_{1Y} = \bar{r}_{2Z} = 2$, and $\omega = 10$. The OR gate parameters are $\bar{r}_{0Y} = \bar{r}_{0Z} = 0$, $\bar{r}_{1Y} = \bar{r}_{1Z} = 2$, $\bar{r}_{2Z} = 10$, and $\omega = 1$. These are the same as in Fig. 22.

the maximum and minimum steady state values of Z. Let's define $\Delta\bar{Z} = |\bar{Z}_f - \bar{Z}_i|$. We want this to be big so that the dynamical change between low and high concentrations is meaningful. The steady state concentrations $\bar{Z}_f$ and $\bar{Z}_i$ have expressions given by

$$\bar{Z}_i = \frac{\bar{r}_{0Z} + \bar{r}_{1Z}(\mathcal{X}_i + \mathcal{Y}_i) + \omega\bar{r}_{2Z}\mathcal{X}_i\mathcal{Y}_i}{1 + \mathcal{X}_i + \mathcal{Y}_i + \omega\mathcal{X}_i\mathcal{Y}_i} \tag{M1}$$

$$\bar{Z}_f = \frac{\bar{r}_{0Z} + \bar{r}_{1Z}(\mathcal{X}_f + \mathcal{Y}_f) + \omega\bar{r}_{2Z}\mathcal{X}_f\mathcal{Y}_f}{1 + \mathcal{X}_f + \mathcal{Y}_f + \omega\mathcal{X}_f\mathcal{Y}_f}. \tag{M2}$$

We observe that both $\bar{Z}_i$ and $\bar{Z}_f$ scale linearly with the rate parameters $\bar{r}_{iZ}$ for $i = 0, 1, 2$. For this reason, in the theoretical setting, a large $\Delta\bar{Z}$ can always be obtained by tuning the rate parameters high. Therefore, we did not include the discussion of this criterion in the main text. Nevertheless, it might be worthwhile to demonstrate the dependence of $\Delta\bar{Z}$ on the dissociation constants $\bar{K}_{XZ}$ and $\bar{K}_{YZ}$. We perform similar sweeps as Fig. 22, except we plot $\Delta\bar{Z}$ for each choice of $(\bar{K}_{XZ}, \bar{K}_{YZ})$. The result is shown in Fig. 35. We employ the same logic gate parameters as in Fig. 22. We observe that the region of large relative $\Delta\bar{Z}$ tends to have an L-shape. The XOR and AND gate have regions of large $\Delta\bar{Z}$ that extend in opposite directions. While XOR gate tends to prefer weak binding, the AND gate benefits from strong binding. The OR gate shape is a superposition of the XOR and AND gate, which is perhaps not surprising since XOR and AND gates are in a sense the limit cases of OR gate. Note that the difference between the magnitude of $\Delta\bar{Z}$ across logic gates is artificial. The absolute magnitude of OR gate $\Delta\bar{Z}$ is large only because $\bar{r}_{2Z} = 10$. We verify the previous claim that $\Delta\bar{Z}$ scales linearly with production rates.

Regarding the average time delay $\langle\Delta\bar{t}\rangle$, we demonstrate computational evidence for the existence of an upper bound on $\langle\Delta\bar{t}\rangle$, given a step in $p^X_{\text{act}}(c_X)$ (fix the high and low values of p^X_{act}). The full set of tunable parameters of the system is $\bar{r}_{iY}, \bar{r}_{jZ}, \omega, \bar{X}, \bar{K}_{XZ}, \bar{K}_{YZ}$, where $i \in \{0, 1\}$ and $j \in \{0, 1, 2\}$. They span a semi-infinite 9-dimensional parameter space. To sweep across this entire space is computationally prohibitive. As a result, we instead take a few 2-dimensional slices to illustrate the existence of the upper bound on $\langle\Delta\bar{t}\rangle$. Specifically, we pair-wise tune 5 different parameters: $\bar{r}_0 = \bar{r}_{0Y} = \bar{r}_{0Z}$, $\bar{r}_1 = \bar{r}_{1Y} = \bar{r}_{1Z}$, $\bar{r}_{2Z}$, ω, and $\bar{X}$. For each parameter combination, we search in K-subspace (as in Fig. 22, $(\bar{K}_{XZ}, \bar{K}_{YZ}) \in [10^{-3}, 10^3] \times [10^{-3}, 10^3]$) and find the combination of $(\bar{K}_{XZ}, \bar{K}_{YZ})$ that generates the maximum $\langle\Delta\bar{t}\rangle$ and record the value of the largest time delay as $\langle\Delta\bar{t}\rangle_{\max}$. In Fig. 36, we plot $\langle\Delta\bar{t}\rangle_{\max}$ as a function of 10 different pair-wise parameters. We find that all parameter combinations yield $\langle\Delta\bar{t}\rangle < 5$. $\langle\Delta\bar{t}\rangle$ remains finite when any parameter (when possible) is tuned towards ∞.

Finally, we address the dependence of $\langle\Delta\bar{t}\rangle$ on the leakiness and saturation of $p^X_{\text{act}}(c_X)$. Here, we denote the minimal $p^X_{\text{act}}(c_X)$ in a step as $p_{\min}$ and the maximal $p^X_{\text{act}}(c_X)$ as $p_{\max}$. After many experimentation with the numerics, we find that the saturation $p_{\max}$ plays a smaller role in determining $\langle\Delta\bar{t}\rangle_{\max}$. The limit $p_{\max} = 1$ yields a similar $\langle\Delta\bar{t}\rangle_{\max}$ as $p_{\max} = 0.95$, and decreasing $p_{\max}$ only makes $\langle\Delta\bar{t}\rangle_{\max}$ smaller. Contrary to $p_{\max}$, $p_{\min}$ plays a much bigger role. We demonstrate this dependency in Fig. 37. Here, we examine the OFF step delay in the XOR coherent feed-forward loop, as from previous sweeps in Fig. 22 and Fig. 36, we see that this tends to be the setting that generates the largest amount of delay. For a given $p_{\min}$, we sweep across $\bar{r}_{1Y} = \bar{r}_{1Z} = \bar{r}_1$, $\bar{X}$. For each combination of $\bar{r}_1$ and $\bar{X}$, we again perform another sweep in $(\bar{K}_{XZ}, \bar{K}_{YZ}) \in [10^{-3}, 10^3] \times [10^{-3}, 10^3]$ to find largest $\langle\Delta\bar{t}\rangle$. We see from Fig. 37 that as $p_{\min}$ decreases, the maximal $\langle\Delta\bar{t}\rangle_{\max}$ increases. We note, however, that $\langle\Delta\bar{t}\rangle_{\max}$ still converges computationally for finite $p_{\min}$. Due to the constraint of the explicit effector function $p^X_{\text{act}}(c_X)$, for any biological parameter $p_{\min}$ is finite. For this reason, we demonstrate the maximum delay corre-

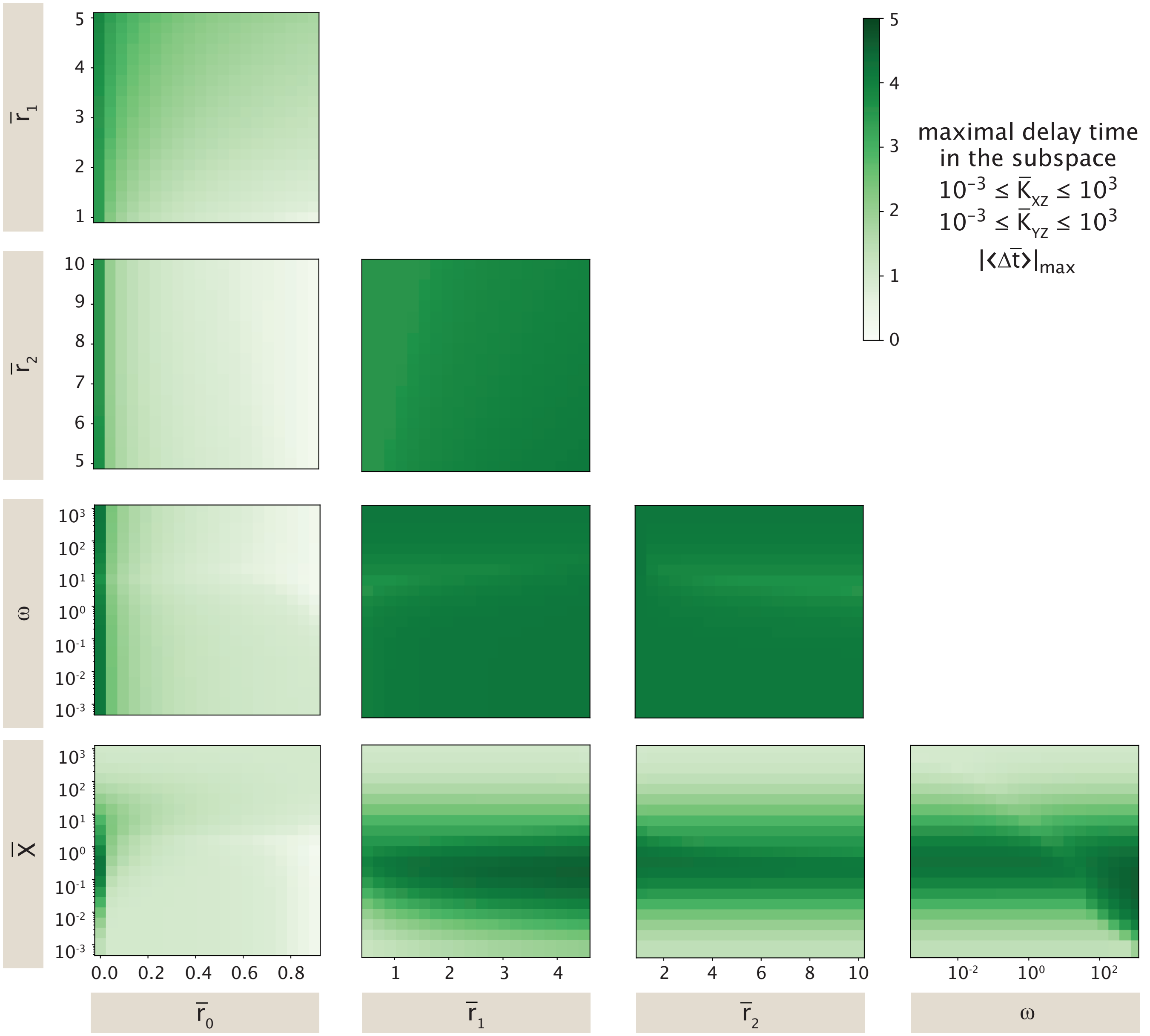

Figure 36: Systematically sweeping across all tunable parameters in coherent feed-forward loops to find the largest $\langle\Delta\bar{t}\rangle$ for the step in $p^X_{\text{act}}(c_X)$ used throughout the feed-forward loop section, where $c_X^{max} = 10^{-4}$ and $c_X^{min} = 10^{-7}$. We pick a default set of parameters: $\bar{r}_{0Y} = \bar{r}_{0Z} = \bar{r}_0 = 0$, $\bar{r}_{1Y} = \bar{r}_{1Z} = \bar{r}_1 = 1$, $\bar{r}_{2Z} = 5$, $\omega = 5$, $\bar{X} = 1$. Note that we set $\bar{r}_{0Y} = \bar{r}_{0Z}$ and $\bar{r}_{1Y} = \bar{r}_{1Z}$ to decrease the number of degrees of freedom in the parameter space without losing too much information. For every colormap, we select a pair of parameters, and perform sweeps on a grid of values, while the other parameters remain the default value. For each value pair, they are used to search in the space $(\bar{K}_{XZ}, \bar{K}_{YZ}) \in [10^{-3}, 10^3] \times [10^{-3}, 10^3]$, and the maximum $\langle\Delta\bar{t}\rangle$ is recorded.

sponding to a biologically sensible set of parameters, and investigate its significance in the main text.

Appendix N: Technical details in incoherent feed-forward loop

In this appendix, we will return to some technical details regarding pulses in the incoherent feed-forward loop, as presented in Section IV C. To begin with, we discuss the quantity $\langle\Delta\bar{t}\rangle$ in incoherent feed-forward loops. In the coherent feed-forward loop, the definition of $\langle\Delta\bar{t}\rangle$ in Eqn. 36 and Eqn. 37 are equivalent, allowing us to interpret it as the average time delay across concentrations. Here, when $\bar{Z}$ does not exhibit a pulse, there is no difference from the coherent case. However, Eqn. 36 becomes ill-defined when $\bar{Z}$ exhibits a pulse. This is because the feed-forward loop response can reach $\bar{Z}$ in a way that the

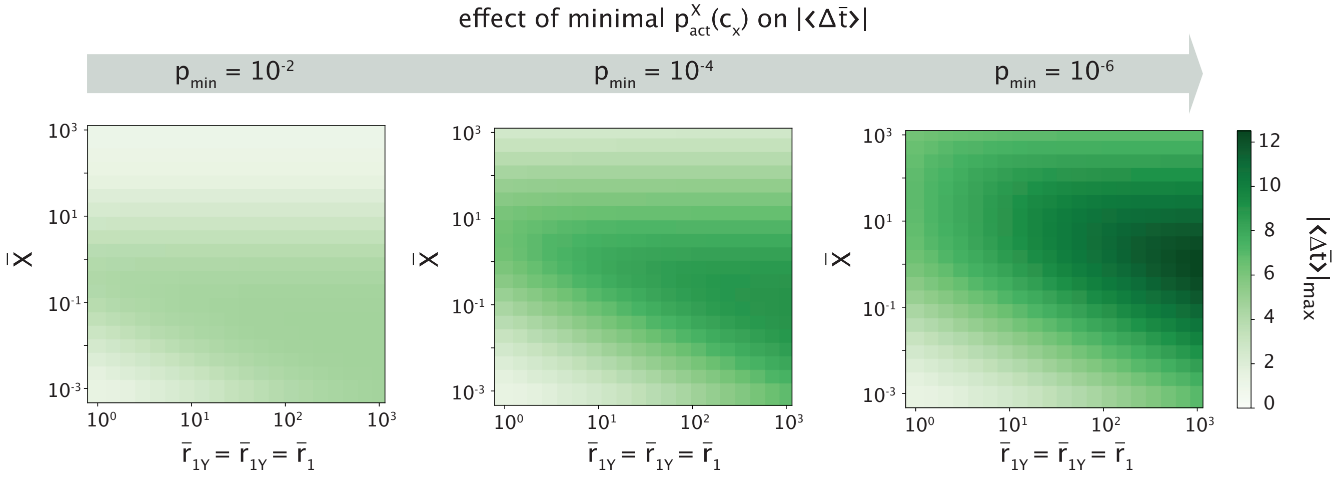

Figure 37: The effect of leakiness of $p^X_{\mathrm{act}}(c_X)$ on $\langle\Delta\bar{t}\rangle$. We examine the dependence $\langle\Delta\bar{t}\rangle$ on minimal $p^X_{\mathrm{act}}(c_X)$ in a step, $p_{\min}$, in the XOR gate setting. Parameters used are $\bar{r}_{0Y} = \bar{r}_{0Z} = 0$, $\omega = 0$. The value of $\bar{r}_{1Y} = \bar{r}_{1Z} = \bar{r}_1$ and $\bar{X}$ are tuned to find the largest $\langle\Delta\bar{t}\rangle$ in that parameter subspace. The detailed sweep process is identical to that in Fig. 36.

simple regulation curve cannot. A value can still be computed for $\langle\Delta\bar{t}\rangle$ using Eqn. 37, but it contains information about the relative size of the pulse rather than acceleration. Because it has unit of time, we regard the direct magnitude of the pulse $\bar{Z}_{\max} - \bar{Z}_f$ for an increasing $\bar{Z}$ response in the main text.

Next, let's derive the maximal average time acceleration $\langle\Delta\bar{t}\rangle_{\max}$ when a pulse does not exist. The green curve shown in Fig. 24(C) and (D) is the trajectory that has the largest acceleration; denote this trajectory to be $\bar{Z}_{\mathrm{step}}(\bar{t})$. Mathematically, $\bar{Z}_{\mathrm{step}}(\bar{t}) = \bar{Z}_f$ for $\bar{t} > 0$. The simple regulation curve is again

$$\bar{Z}_{\mathrm{simple}}(\bar{t}) = \bar{Z}_i e^{-\bar{t}} + \bar{Z}_f(1 - e^{-\bar{t}}). \tag{N1}$$

From Eqn. 37, we can then compute the maximal average time acceleration as

$$\begin{aligned}\langle\Delta\bar{t}\rangle_{\max} &= \frac{1}{\bar{Z}_f - \bar{Z}_i}\int_0^\infty d\bar{t}\,\left(\bar{Z}_{\mathrm{step}}(\bar{t}) - \bar{Z}_{\mathrm{simple}}(\bar{t})\right)\\ &= \frac{1}{\bar{Z}_f - \bar{Z}_i}\int_0^\infty d\bar{t}\,\Big(\bar{Z}_f - \bar{Z}_i e^{-\bar{t}} - \bar{Z}_f(1 - e^{-\bar{t}})\Big)\\ &= \frac{1}{\bar{Z}_f - \bar{Z}_i}\int_0^\infty d\bar{t}(\bar{Z}_f - \bar{Z}_i)e^{-\bar{t}}\\ &= \frac{\bar{Z}_f - \bar{Z}_i}{\bar{Z}_f - \bar{Z}_i} = 1.\end{aligned} \tag{N2}$$

Finally, we discuss the definition of strong pulse used in Fig. 24. Numerically, we characterize a pulse to be strong when the maximum concentration that the transient $\bar{Z}(\bar{t})$ reaches, $\bar{Z}_{\max}$, satisfies $(\bar{Z}_{\max} - \bar{Z}_f)/(\bar{Z}_f - \bar{Z}_i) > 0.05$ for an increasing $\bar{Z}$ response. The threshold of 0.05 is an arbitrary choice. Due to this threshold, there exists a region of trajectories that are technically pulses but are not strong enough to be considered. The trajectory in Fig. 24(C) in fact belongs to this region. It exhibits a pulse strictly speaking. Nevertheless, the magnitude of the pulse is vanishingly small. Functionally, it acts in the same manner as the trajectories that do not possess a pulse.

Appendix O: Effect of $\bar{Y}$ in continuous tuning

In Section IV D, we discussed the scenario where the signal $c_X(\bar{t})$ is no longer a step function, but a continuous function in time. We mentioned that the comparison with simple regulation in this case is subtle, as the arbitrary choice of value $\bar{Y}$ affects the shape of simple regulation response. Here, we expand on Fig. 25 where we repeat the numerical integration for three distinct values of $\bar{Y}$, as shown in Fig. 38. For the fast tuning case, different choices of $\bar{Y}$ have no effect on the simple regulation trajectory. This is expected since in the limit case of a step function signal in c_X, the value of $\bar{Y}$ strictly has no effect on the shape of the trajectory. As the rate of tuning c_X slows down, the effect of $\bar{Y}$ becomes more and more pronounced. Specifically, as $\bar{Y}$ increases, the rescaled simple regulation curve "shrinks". As a result, the magnitude of apparent delay/acceleration between the feed-forward loop and simple regulation response increases. Due to the artificial nature of the choice of $\bar{Y}$ in simple regulation, we cannot make a claim about the magnitude of delay/acceleration in the slow tuning limit. Nevertheless, our qualitative results stand. The choice of $\bar{Y}$ does not change the type of response to a step. For example, even though $\bar{Y}$ affects the magnitude of delay on the OFF step, the feed-forward

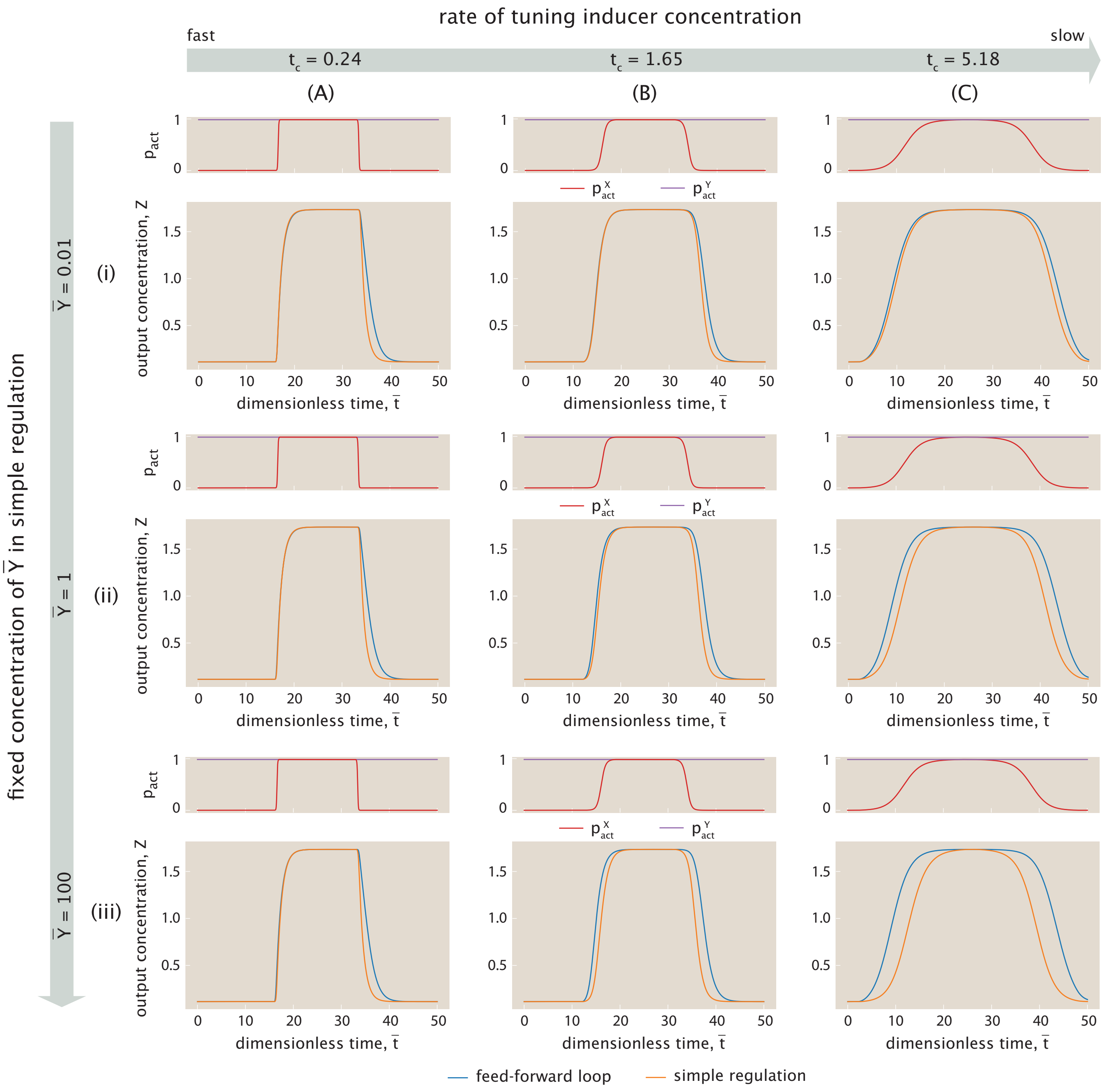

Figure 38: Demonstrating the how the arbitrary choice of $\bar{Y}$ affects the simple regulation dynamics. We replicate Fig. 25 with different choices of $\bar{Y}$. From top to bottom, $\bar{Y}$ is set to be 0.01 in (i), 1 in (ii), which matches the setting in Fig. 25, and 100 in (iii). From left to right, the rate of concentration is tuned the same way as in Fig. 25. System parameters are also identical to those in Fig. 25.

loop in this case always delays on the OFF step.

Appendix P: Code availability

All Jupyter notebooks used to generate graphs in figures throughout this paper are available [107].
